# Novel sources of variation in grain Zinc (Zn) concentration in bread wheat germplasm derived from Watkins landraces

**DOI:** 10.1371/journal.pone.0229107

**Published:** 2020-02-28

**Authors:** Jaswant S. Khokhar, Julie King, Ian P. King, Scott D. Young, Michael J. Foulkes, Jayalath De Silva, Minuka Weerasinghe, Abdul Mossa, Simon Griffiths, Andrew B. Riche, Malcolm Hawkesford, Peter Shewry, Martin R. Broadley

**Affiliations:** 1 School of Biosciences, University of Nottingham, Sutton Bonington Campus, Loughborough, United Kingdom; 2 Crop Genetics, John Innes Centre, Norwich, United Kingdom; 3 Plant Sciences, Rothamsted Research, Harpenden, United Kingdom; Institute of Genetics and Developmental Biology Chinese Academy of Sciences, CHINA

## Abstract

A diverse panel of 245 wheat genotypes, derived from crosses between landraces from the Watkins collection representing global diversity in the early 20^th^ century and the modern wheat cultivar Paragon, was grown at two field sites in the UK in 2015–16 and the concentrations of zinc and iron determined in wholegrain using inductively coupled plasma-mass spectrometry (ICP-MS). Zinc concentrations in wholegrain varied from 24–49 mg kg^-1^ and were correlated with iron concentration (r = 0.64) and grain protein content (r = 0.14). However, the correlation with yield was low (r = -0.16) indicating little yield dilution. A sub-set of 24 wheat lines were selected from 245 wheat genotypes and characterised for Zn and Fe concentrations in wholegrain and white flour over two sites and years. White flours from 24 selected lines contained 8–15 mg kg^-1^ of zinc, which was positively correlated with the wholegrain Zn concentration (r = 0.79, averaged across sites and years). This demonstrates the potential to exploit the diversity in landraces to increase the concentration of Zn in wholegrain and flour of modern high yielding bread wheat cultivars.

## Introduction

Zinc (Zn) and iron (Fe) are essential micronutrients for human health. Inadequate dietary intakes of Zn and Fe are linked to diarrhoea, stunting (low height for age in childrem) and anaemia, and to increased mortality [1–3]. Approximately, 20% of the global population has insufficient Zn in their diets, rising to 50% in parts of South Asia and sub-Saharan countries where diets are based on cereals crops including wheat [4]. In the UK, 11% children between 4 and 10 years of age have Zn intakes below than lower reference nutrient intake value and Fe deficiency has increased up to 9% in adolescent girls and 5% in women. After red meat and processed meats, bread wheat and products are the main sources of dietary Zn and Fe intake in the UK population [5, 6].

Bread wheat (*Triticum aestivum* L.) is an important cereal crop globally and provides up to 60% of the total energy intake in some countries (FAOSTAT, 2013; http://www.faostst.fao.org) and is also a major source of daily dietary Zn and Fe [7]. Modern wheat varieties typically have whole-grain Zn concentration less than the target of 40 mg kg^-1^ considered appropriate for human diets under field conditions in S. Asia [8]. For example, grain Zn concentration ranged from 25–35 mg kg^-1^ in a panel of 36 widely adapted Indian wheat genotypes under field conditions [9].

Grain Zn concertation in wheat can be increased by agronomic and genetic biofortification. Agronomic biofortification involves the use of Zn fertilisers, which can increase both crop yield and grain Zn concentration in wheat. However, it also increases the cost and may require to changes to current farming practices because Zn applied to soils at normal agronomic rates is unlikely to have large effects on grain Zn concentration [8, 10, 11]. Agronomical biofortified wheat flour using foliar applications was provided to children between 4 to 6 years of age for six months and showed 17% and 40% reduction in incidences of pneumonia and vomiting, respectively [12].

Genetic Zn biofortification of wheat is likely to be more cost-effective in the longer term than the use of fertilisers, but requires the existence of genetic variation in wheat germplasm. Wild wheat species and landraces have shown variation for grain Zn concentration in wheat and may therefore be used to achieve the breeding target of 40 mg kg^-1^ grain Zn in wheat [8, 11, 13]. The ‘HarvestPlus’ programme has been the primary driver of genetic biofortification in wheat. It has developed and deployed high-Zn wheat varieties in South Asia using synthetic wheat lines derived from wild wheat relatives, *Aegilops tauschii* (D genome donor of wheat), *Triticum spelta* and wild *T*. *dicoccon*. HarvestPlus has released locally adapted high-Zn wheat varieties in India and Pakistan with 30–40% higher Zn in grain than a notional baseline of 25 mg kg^-1^ grain Zn [14]. Cakmak *et al*. [15] reported grain Zn concentration ranging from 30–98 mg kg^-1^ in 518 accessions of *T*. *dicoccoides* grown in the field, while Guzman *et al*. [16] screened 93 advance lines, which were developed by the crossing of Mexican landraces, *Triticum dicoccoides* and *Ae*. *tauschii* with durum and wheat genotypes, grown under field condition in Mexico and reported grain Zn concentration from 27–53 mg kg^-1^. Similarly, Srinivasa *et al*. [17] reported grain Zn concentration from 25–60 mg kg^-1^ in 185 recombinant inbred lines derived from crossing between *T*. *spelta* and *T*. *aestivum* grown under field conditions.

Most studies of wild species, landraces and their derivatives report Zn concentration in whole grain not in wheat white flour (endosperm). Whereas the wheat flour (*atta*) used to prepare unleavened flatbreads (chapattis or rotis) in South Asia comprises about 95% of the whole grain, white wheat flour is commonly used to make leavened bread (yeast fermented) and other products in Europe, the USA and Oceania countries (Australia and New Zealand) [18]. This corresponds to the starchy endosperm tissue and is produced by milling to remove the aleurone, outer layers and bran. Because Zn accumulates in the aleurone layer and embryo [19–21], white flour may contain <10 mg kg^-1^ Zn which is not sufficient for human diet [20]. Furthermore, the bioavailability of the Zn in the aleurone and embryo is reduced by the presence of phytic acid, the major storage form of phosphorus which is present in the same tissues [22]. Phytate forms insoluble complexes with Zn and inhibits its absorption in monogastric animals [23]. Therefore, screening of wider germplasm for variation in Zn concentration in white flour should allow the development of wheat varieties with more bioavailable Zn in white flour.

The objectives of this study were to characterise a diverse panel of wheat genotypes, derived from landraces, and the UK cultivar Paragon, grown on two sites in the UK for variation in wholegrain Zn concentration. Based on these analyses a sub-set of genotypes were selected and analysed for Zn in both wholegrain and white flours using material grown in two years and on two sites. This identified lines which are suitable for exploitation in breeding to produce cultivars with high bioavailable Zn.

## Materials and methods

### Plant material

A panel of 245 wheat genotypes, derived from crosses between landraces from the Watkins collection and the UK Spring wheat cultivar Paragon, were grown at Bunny Farm site of the University of Nottingham in 2015–16 and 2016–17 and, at the Rothamsted Research farm in 2015–16 and 2017–18. The landraces were collected from 34 countries by A.E. Watkins in the early 1930s [24]. Marker analysis allowed a core set of 119 lines to be identified and 85 of these crossed with Paragon to develop F1 progeny, followed by four to six rounds of selfing (F4: F6) to develop populations of recombinant inbred lines [24, 25]. Each population comprises 94 lines, and 12 or 13 lines from 19 populations were selected for the present project to constitute a panel of 245 wheat genotypes (S1 Table). These lines were selected based on their performance (including yields) and adaptation to the UK (including flowering time).

### Experimental sites and management

The details of field experiments conducted at the University of Nottingham and Rothamsted Research, sites are described in Table 1.

**Table 1:** Field experiments details.

Site	Sowing	Harvesting	Year	Grid	Previous Crop
**Nottingham**	05.04.2016	01.09.2016	2016	52˚51’ 45” N	Oilseed rape
	27.03.2017	24.08.2017	2017	1˚7’ 30.6” W	Oilseed rape
**Rothamsted**	21.03.2016	1–2.09.2016	2016	51˚48’ 18” N	Wheat
	26.10.2017	21–22.08.2018	2018	0˚ 23’ 01” W	Winter Oat

The plots were arranged in a randomised factorial block design with three replications at both the sites. The plot size was 1 m^2^. To achieve a target population size of 200 plants m^-2^, seed rates were selected on 1000 grain weight (TGW) basis and, ~350 seeds m^-2^ were used per plot. Seeds were planted using a power drill after field preparation.

Nitrogen fertiliser at Nottingham was applied at the rate of 175 kg ha^-1^ as Nitram (34.5% N), one-third of total N was applied at sowing time and the remaining was applied at growth stage 31, onset of stem extension (GS31) in 2015–16 and 2016–17. At Rothamsted nitrogen fertilisers were applied in two doses; first dose at the rate of 222 kg ha^-1^ as double top (27% N) and 2^nd^ dose @ of 101 kg ha^-1^ as ammonium nitrate (34.5% N) in 2015–16 while in 2017–18 only a single dose of ammonium nitrate was applied at the rate of 370 kg ha^-1^. All the plots were managed and protected from pest, diseases, and weeds by using herbicides and pesticides to ensure to get the maximum yield. Crops were harvested at maturity by a small combine harvester.

### Grain sampling and digestion of 245 wheat genotypes

Ears were harvested from an area of 0.5-meter length from central rows of each plot, dried at 80°C for 48 hours in the oven and then threshed mechanically. Approximately 10 grains of all 245 wheat genotypes in three replicates from Nottingham (n = 735) and in one replicate from Rothamsted site (n = 245) in 2015–16 were weighed and the grain samples were soaked in this solution, 3 mL 70% Trace Analysis Grade (TAG) HNO_3_ and 2 mL hydrogen peroxide (H_2_O_2_), overnight at room temperature. Grain was digested using a microwave system as described previously [9, 26]. Briefly, this comprised a Multiwave 3000 platform with a 48-vessel MF50 rotor (Anton Paar Gmbh, Graz, Austria); digestion vessels were perfluoroalkoxy (PFA) tubes in polyethylethylketone (PEEK) pressure jackets (Anton Paar GmbH). Grains were digested in 3 mL 70% Trace Analysis Grade (TAG) HNO_3_ and 2 mL hydrogen peroxide (H_2_O_2_).

For total nitrogen analysis, wholegrain wheat subsamples were digested using the Kjeldahl method [27]. Briefly, the Kjeldahl system comprised a heating platform with a 42-vessel digestion unit, a scrubber unit with 20% sodium hydroxide neutraliser and a recirculating water pump (VELP, town, Germany). Wholegrain wheat subsampales (~0.1 g) were digested in 8 mL 95% sulphuric acid (Fisher Scientific UK Ltd, Loughborough, UK). The Kjeldahl settings were as follows: temp = 390°C, water pump pressure = 1.5 bar, time = 120 minutes. Three operational blanks were included in each digestion run and, duplicate samples of certified reference material (CRM: Wheat flour SRM 1567b, NIST, Gaithersburg, MD, USA) were used in every third run. After digestion, each tube was made up to a final volume of 50 mL by adding 11 mL de-ionsed water and transferred to falcon tubes. The nitrogen concentration of wholegrain wheat subsamples were determined by using a microtiter plate and the colorimetric salicylic acid method [28]. Nitrogen % was converted into grain protein content (GPC%) by multiplying the grain N% with conversion factor 5.7 (N% x 5.7), according to the AACCI Methods 46–30 [29].

### Milling and digestion of a sub-set 24 wheat genotypes

Based on the concentrations of grain Zn and Fe in the wholegrain samples of a panel of 245 wheat genotypes grown at Nottingham and Rothamsted in 2015–16, a sub-set of 24 genotypes was selected to capture the diversity present in the panel. Grain samples of these genotypes were resampled from three replicate plots per line from Nottingham in 2015–16, and from freshly harvested plots in 2016–17, and resampled from three replicate plots per line from Rothamsted in 2015–16, and from freshly harvested plots in 2017–18. Samples were dried at 40°C overnight before milling, 6 g subsamples of grain were then milled at 30 rpm for 30 seconds using a Laboratory Flour Mill AQC 806 (Agromatic AG, Laupen, Switzerland) to obtain wheat white flour. Wholegrain samples (n = 129) of these sub-sets of genotypes, resampled from the Nottingham and Rothamsted 2015–16 material, were digested in a similar manner as the 245 wheat genotypes, with a soaking step. The subsample (~0.300 g DW) of wheat white flour samples (n = 129) were digested without soaking step. The wholegrain (n = 114) and white flour (n = 114) samples of these sub-sets of genotypes from Nottingham in 2016–17 and from Rothamsted in 2017–18 were digested using a Multiwave Pro platform with 41-vessels, HVT56 rotor system where microwave settings were: power = 1500 W, temperature = 175°C, and time = 40 minutes. Wholegrain and white flour samples were digested in 6 mL 70% TAG HNO3 solution. The wholegrain samples were soaked in this solution for one hour at room temperature.

Two operational blanks were included in each digestion run. Duplicate samples of certified reference material (CRM: Wheat flour SRM 1567b, NIST, Gaithersburg, MD, USA) were included approximately every fourth digestion run. A laboratory reference material (LRM), Paragon was used for each digestion run. In 2015–16, following digestion, each tube was made up to a final volume of 15 mL by adding 11 mL Milli-Q water, then transferred to a 25 mL universal tube (Sarstedt Ltd., Nümbrecht, Germany) and stored at room temperature. Samples were further diluted 1:5 with Milli-Q water into 13 ml tubes (Sarstedt Ltd.) prior to analysis. In 2016–17, each tube was made up to a final volume of 24 mL by adding 18 mL Milli-Q water, then transferred to a 25 mL universal tube and stored at room temperature. Samples were further diluted 1:10 with Milli-Q water into 13 ml tubes prior to analysis.

### Wholegrain and white flour Zn and Fe analysis

Wholegrain and white flour concentration of Zn and Fe were determined by ICP-MS (Thermo Fisher Scientific iCAPQ, Thermo Fisher Scientific, Bremen, Germany). The other elements also measured were Ag, Al, As, B, Ba, Be, Ca, Cd, Cr, Co, Cs, Cu, K, Li, Mg, Mn, Mo, Na, Ni, P, Pb, Rb, S, Se, Sr, Ti, Tl, U, and V, but these are not reported in this study. The other operation details and setting of ICP-MS were described in Khokhar *et al*. [9]. In total 980 wholegrain samples of a panel of 245 wheat genotypes, 735 from Nottingham and 245 from Rothamsted harvested in 2015–16 were analysed in 23 runs of microwave digestion. In addition, 129 wholegrain and 129 wheat white flour samples of a sub-set of 24 genotypes were analysed from both the sites in 2015–16. In 2016–17, 114 wholegrain and 114 wheat white flour samples of sub-set genotypes were analysed from both the sites. Grain samples of five genotypes in sub-set of 24 genotypes were not available from Rothamsted in the first-year harvest and from both the sites in the second-year harvest. All the wholegrain and white flour sample numbers were excluding blanks, LRM and CRM samples. The respective Zn and Fe recovery from CRMs were 97.5% and 81% for samples from 2015–16, and 96.7% and 112.2% for samples from 2016–17.

Limits of detection (LODs) of all measured elements were reported as 3 times the standard deviation (SD) of all the operational bank concentrations and, an element concentration less than the LOD values, real values were replaced by half LOD value. Further details of LODs calculations and data normalisation are described in Khokhar *et al*. [9]. The respective LOD for Zn and Fe were 0.57 and 3.14 mg kg^-1^ for samples from 2015–16, and 1.71 and 4.88 mg kg^-1^ for samples from 2016–17.

### Statistical analysis

Variance components associated with Zn and Fe concentrations in wholegrain, GYD, TGW and GPC were calculated. Analyses of Variance (ANOVA) and Least Significant Difference (LSD) tests were used to test for differences in mean wholegrain elemental concentration between sites and wheat genotypes. Differences between sites and genotypes for wholegrain and white flour Zn and Fe concentrations traits were considered significant at P<0.001. Pearson correlation coefficients were calculated to study the relationships for Zn and Fe concentration and yield in the wholegrain and white flour. All analyses were conducted using GenStat 17^th^ Edition (VSN International Ltd, Hemel Hempstead, UK).

## Result

### Variation in wholegrain Zn, Fe concentration grain yield, TGW and GPC of 245 Watkins derived wheat genotypes

The mean wholegrain Zn concentration across all plots (n = 980) was 33.6 mg kg^-1^ and varied from 15.6 to 60.1 mg kg^-1^ in 2015–16 (Table 2). The mean wholegrain Fe concentration was 34.3 mg kg^-1^ and varied from 19.4 to 71.2 mg kg^-1^, across all plots. The mean GYD was 5.2 t ha^-1^ and varied from 0.31 to 9.76 t ha^-1^, across all plots. The mean TGW was 34.4 g and varied from 22.2 to 47.7 g, across all plots. The mean grain protein content (GPC) was 15.4% and varied from 5.62 to 27.3%, across all plots (Table 2). Primary data of 245 lines are provided in S2 Table.

**Table 2:** Wholegrain Zn, Fe concentrations, grain yield, TGW and GPC of 245 Watkins derived wheat genotypes. Wholegrain Zn and Fe concentration data are in mg kg^-1^, GYD is in t ha^-1^, TGW is in grams and grain protein content is in %, summarised across all plots (n = 980). Data are of 735 plots at Nottingham and, 245 plots at Rothamsted in 2015–16.

Element	N	Mean	Median	Range	SD	LOD
**Zn**	980	33.6	33.4	15.6–60.1	7.26	1.32
**Fe**	980	34.3	33.5	19.4–71.2	6.38	4.18
**GYD**	980	5.20	4.94	0.311–9.76	1.64	
TGW	980	34.4	34.3	22.2–47.7	3.85	
GPC	980	15.4	15.2	5.62–27.3	3.1	

Genotype accounted for 30% and 38% of the variation in wholegrain Zn and Fe concentrations, respectively (Table 3). Site accounted for a greater proportion of the variation in wholegrain Zn concentration (24%) than wholegrain Fe concentration (3.7%). The G*E interaction term was associated with 8% of the variation in wholegrain Zn and Fe concentrations. The residual term (R) was associated with 38% and 50% of the variation in wholegrain Zn and Fe concentration, respectively; this could be due to plot to plot variation and technical/measurement errors (Table 3). Genotype and site accounted for 23% and 56% of the variation in GYD, respectively. Genotype accounted for a greaer proportion of the variation in TGW (63%) than GPC (28%). Similarly, site accounted for a greater proportion of the variation in TGW (19%) than GPC (8%). The residual factor accounted for a greater proportion of the variation in GPC (52%) than in GYD (14%) and TGW (14%).

**Table 3:** Variation in wholegrain Zn and Fe concentrations, GYD, TGW and GPC due to G, E, G*E and, Residual variance component factors in 245 Watkins and Paragon derived wheat genotypes grown at Nottingham and Rothamsted in 2015–16.

	Variation %						
	G	E	G*E	Residual	G	E	G*E
**Zn**	29.5	24.3	8.15	37.9	< .001	< .001	1
**Fe**	38.3	3.72	8.41	49.6	< .001	< .001	1
**GYD**	23.1	56.1	6.50	14.3	< .001	< .001	0.9
**TGW**	63.0	19.1	5.04	14.3	< .001	< .001	0.8
**GPC**	28.1	8.4	11.13	52.3	0.54	< .001	1
***df***	***244***	***1***	***233***	***471***			

### Variation in wholegrain Zn, Fe concentrations, grain yield, TGW and GPC between sites

**Fig 1:**
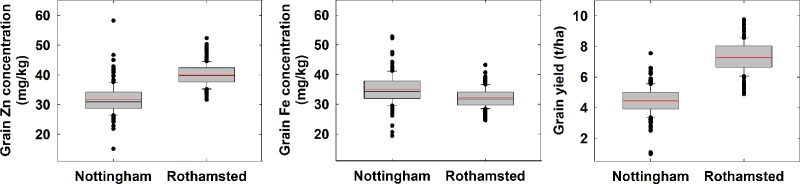
Wholegrain Zn and Fe concentration and grain yield of 245 Watkins x Paragon derived wheat genotypes grown at Nottingham and Rothamsted sites in 2015–16. Data are means of three replicate plots per genotype at Nottingham and one replicate plot per genotypes at Rothamsted in 2015–16. Boxes represent the two mid-quartile with the median (black line) and mean (red line) drawn; whiskers are the 95% confidence limits; circle is outliers.

Grain yield and wholegrain Zn concentration were lesser at Nottingham than at Rothamsted (P<0.001) (Fig 1). The mean grain yield, averaged across 245 wheat genotypes in 2015–16, varied from 4.46 (Nottingham) to 7.29 (Rothamsted) t ha^-1^ (LSD = 1.38). The mean wholegrain Zn concentration averaged across 245 wheat genotypes in 2015–16, varied from 31.6 (Nottingham) to 40 (Rothamsted) mg kg^-1^, (LSD = 7.07). The wholegrain Fe concentration varied from 35.1 mg kg^-1^ (Nottingham) to 32.2 mg kg^-1^ (Rothamsted) (LSD = 6.21) (Fig 1). The mean TGW, averaged across 245 wheat genotypes in 2015–16, varied from 33.4 g (Nottingham) to 37.4 g (Rothamsted) (LSD = 2.79). The mean GPC varied from 15.8% (Nottingham) to 13.9% (Rothamsted) (LSD = 4.66).

The wholegrain Zn concentration of 245 wheat genotypes, averaged across three replicates in 2015–16, was positively correlated with wholegrain Fe concentration at Nottingham (r = 0.64; P<0.001) and at Rothamsted (r = 0.65; P<0.001). The wholegrain Zn concentration showed a significant weak negative relationship with grain yield at Nottingham (r = -0.21; P<0.001) and Rothamsted (r = -0.27; P<0.001) sites in 2015–16. The wholegrain Zn concentration of 245 wheat genotypes was significantly positively correlated with GPC at Nottingham (r = 0.14; P<0.05) and at Rothamsted (r = 0.52; P<0.001), however, wholegrain Zn concentration showed a non-significat weak correlation with TGW at Nottingham (r = 0.11; P = 0.086) and a non-significant negative weak correlation at Rothamsted (r = -0.12; P = 0.065). The TGW showed positive significant correlation with GYD at Nottingham (r = 0.14; P<0.05) and at Rothamsted (r = 0.27; P<0.001), but not correlated with GPC at both, Nottingham and Rothamsted sites. The grain protein content (GPC) showed significant negative correlation with GYD at Nottingham (r = -0.14; P<0.05) and at Rothamsted (r = -0.28; P<0.001).

### Variation in wholegrain Zn, Fe concentrations, GYD, TGW and GPC between 245 wheat genotypes

**Fig 2:**
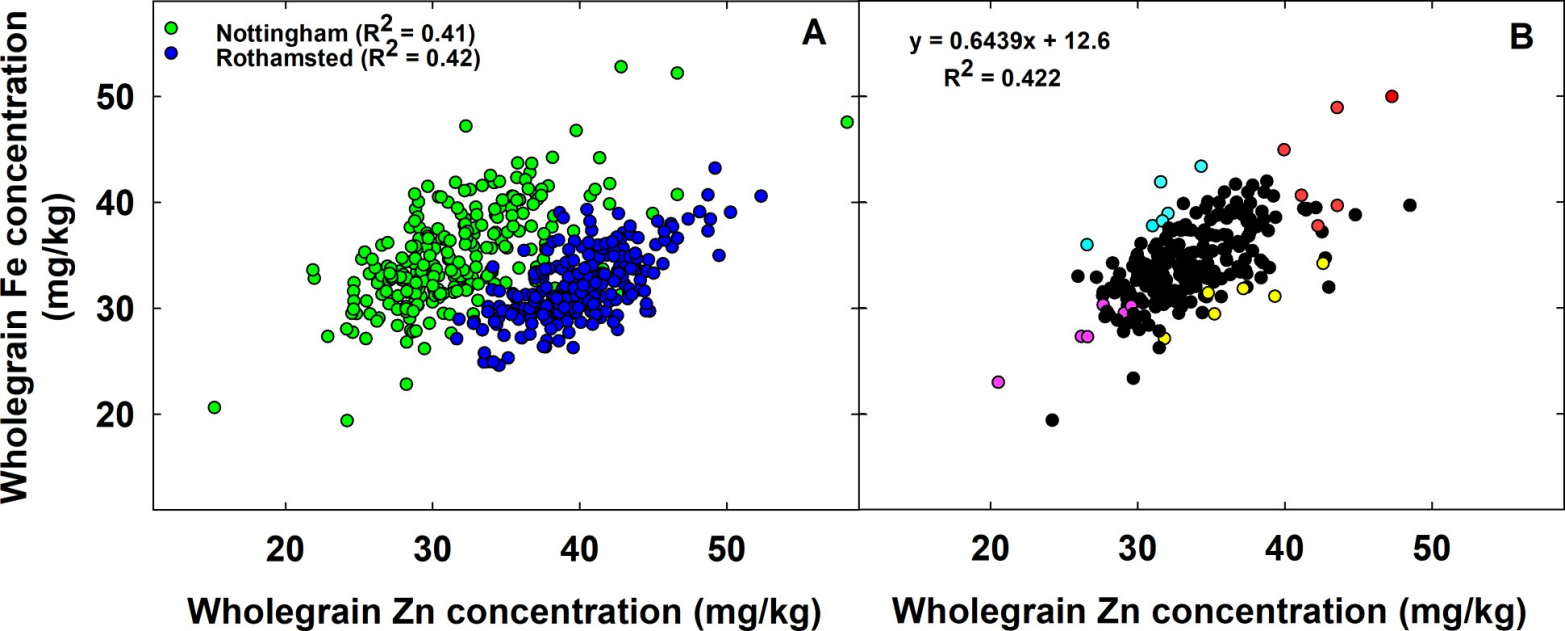
**(A)** The correlation between wholegrain Zn and Fe concentration in a panel of 245 wheat genotypes at the Nottingham (Green circle) and Rothamsted (Blue circle) sites in 2015–16. **(B)** Wholegrain Zn and Fe concentrations in a panel of 245 Watkins and Paragon derived wheat genotypes, averaged across two sites in 2015–16. Data are the means of three replicate plots per genotype at Nottingham and one replicate plot per genotype at Rothamsted in 2015–16. Out of 245, a sub-set of 24 genotypes in different Zn and Fe level combinations were selected and divided into four groups. Group 1: High Zn-High Fe (H Zn-H Fe; Red circle); Group 2: High Zn-Low Fe (H Zn-L Fe; Yellow circle); Group 3: Low Zn-High Fe (L Zn-H Fe; Cyan circle) and Group 4: Low Zn Low Fe (L Zn-L Fe; Pink circle).

The Zn concentration in wholegrain of 245 wheat genotypes varied from 24.2 (PxW42-60) to 48.5 (PxW349-46) mg kg^-1^ in 2015–16 (mean of both sites, Fig 2B) and the Fe concentration varied 19.4 (PxW42-60) to 47.7 (PxW7-76) mg kg^-1^ in 2015–16 (mean of both sites, Fig 2B). Grain yield of 245 wheat genotypes varied from 2.9 (PxW349-46) to 8.3 (PxW546-3) t ha^-1^ in 2015–16 (mean of both sites). The TGW of 245 wheat genotypes varied from 27.5 (PxW223-80) to 44.1 (PxW546-27) g in 2015–16. Grain Proetin content (GPC) varied from 12.3 (PxW685-41) to 18.6 (PxW546-25) % in 2015–16 (mean of both sites). The wholegrain Zn and Fe concentrations of Paragon was 34.4 and 29.6 mg kg^-1^, averaged across both sites in 2015–16, respectively.

The wholegrain Zn concentration of 245 wheat genotypes correlated positively with wholegrain Fe concentration at both the Nottingham (r = 0.65; P<0.001) and Rothamsted (r = 0.65; P<0.001) sites in 2015–16 (Fig 2A). The wholegrain Zn concentration of 245 wheat genotypes was positively correlated with wholegrain Fe concentration (r = 0.65; P<0.001), averaged across both sites in 2015–16 (Fig 2B).

A sub-set of 24 wheat genotypes were selected for further analysis of white flour Zn and Fe concentration, representing six genotypes from each of four Zn and Fe level combinations (Fig 2B): 1) High Zn-High Fe (H Zn-H Fe); 2) High Zn-Low Fe (H Zn-L Fe); 3) Low Zn-High Fe (L Zn-H Fe) and 4) Low Zn-Low Fe (L Zn-L Fe).

### Relationships between Zn and Fe concentrations in wholegrain and white flour of a sub-set of 24 genotypes

There were positive correlations between the Zn concentrations in wholegrain of the 24 genotypes grown at Nottingham and Rothamsted in 2015–16 (r = 0.52; P<0.05) and in 2016–17 (r = 0.44; P = 0.06). Within the sites, the mean wholegrain Zn concentrations of 24 genotypes correlated between 2015–16 and 2016–17 at Nottingham (r = 0.77; P<0.001) and Rothamsted (r = 0.46; P<0.05). The genotypes showed consistent differences in wholegrain Zn concentration between the years at each site. For example, the PxW546-20 genotype showed largest wholegrain Zn concentration in 2015–16 and 4^th^ largest in 2016–17 at Nottingham site and PxW396-56 genotype showed largest wholegrain Zn concentration in 2015–16 and 3^rd^ largest in 2017–18 at Rothamsted (Table 4). The primary data of 24 wheat lines are provided in S3 Table.

**Table 4:** Zinc and Iron concentrations in wholegrain and white flour in sub-set of 24 Watkins derived wheat genotypes grown at Nottingham and Rothamsted sites over two years, 2015–18. Data are means of three replicate plots per genotypes at each site and year. Data is in mg kg^-1^.

		Nottingham-2016				Nottingham-2017				Rothamsted-2016				Rothamsted-2018			
Genotype	Zn & Fe Levels	Zn		Fe		Zn		Fe		Zn		Fe		Zn		Fe	
		Wholegrain	White flour	Wholegrain	White flour	Wholegrain	White flour	Wholegrain	White flour	Wholegrain	White flour	Wholegrain	White flour	Wholegrain	White flour	Wholegrain	White flour
**PxW216–88**	H Zn-L Fe	27.99	11.88	36.41	20.56	23.01	7.76	43.97	14.04	42.45	13.16	39.23	23.31	40.35	12.94	52.47	13.26
**PxW223–80**	H Fe-L Zn	27.18	10.47	35.01	20.13												
**PxW254–2**	H Zn-L Fe	32.72	11.61	29.54	19.27	24.52	9.33	37.20	13.80	40.23	13.76	27.25	16.22	32.52	11.01	37.74	8.13
**PxW264–17**	H Zn-L Fe	28.29	8.92	27.42	14.18	22.72	6.91	31.97	9.13	40.13	13.28	25.88	9.60	53.52	17.26	49.46	12.35
**PxW264–50**	H Fe-L Zn	28.68	7.77	30.96	10.34	19.75	5.30	35.40	8.94	41.99	12.17	32.12	9.65	38.80	11.19	44.13	9.27
**PxW273–21**	L Fe-L Zn	22.84	7.71	27.65	26.30	18.20	5.48	31.36	9.83	33.32	11.98	25.22	21.2	43.49	14.29	49.96	11.08
**PxW273–71**	H Zn-L Fe	28.17	9.42	27.27	14.95	24.22	7.68	33.42	9.06	37.73	13.26	24.18	16.20	38.78	11.40	45.34	9.76
**PxW291–23**	L Fe-L Zn	24.90	10.76	24.58	15.60	22.67	7.41	38.56	10.19	28.35	10.07	28.80	11.98	37.94	13.71	43.28	10.30
**PxW291–39**	H Fe-L Zn	27.87	8.53	29.02	9.93												
**PxW291–75**	L Fe-L Zn	23.97	8.05	24.36	10.85	16.35	4.74	30.03	8.62	30.30	7.98	24.77	12.45	35.55	11.59	38.50	10.29
**PxW299–87**	L Fe-L Zn	23.08	7.55	27.75	13.12	18.84	5.57	32.91	10.32	30.11	8.46	25.58	10.87	28.93	9.53	35.03	10.43
**PxW396–56**	H Zn-L Fe	32.30	12.80	34.52	23.84	26.64	9.98	39.63	15.93	53.27	15.44	33.40	14.22	48.24	16.75	50.95	12.94
**PxW398–18**	H Fe-L Zn	25.04	10.09	36.16	22.42												
**PxW546–20**	H Zn-L Fe	44.39	16.45	35.89	17.02	25.42	10.53	34.77	15.40	38.25	17.05	27.00	15.43	46.09	14.76	47.19	11.06
**PxW546–24**	H Zn-L Fe	32.97	14.33	27.82	19.52	25.25	9.62	33.71	11.42	41.41	12.90	27.06	12.26	52.12	15.99	44.75	12.26
**PxW546–25**	H Fe-L Zn	29.90	13.27	33.05	20.81												
**PxW566–20**	L Fe-L Zn	26.81	10.19	27.93	20.21	21.32	7.38	27.97	10.33	31.04	10.90	22.53	18.49	40.14	12.89	40.75	10.98
**PxW685–36**	H Zn-H Fe	36.45	13.30	35.93	14.13	30.19	11.38	41.22	11.80	39.20	14.12	30.77	16.38	40.96	12.84	47.35	9.97
**PxW7–2**	H Zn-H Fe	32.52	13.77	38.38	22.76	24.60	8.10	41.63	12.56	52.99	16.99	37.91	14.50	41.80	14.32	46.92	10.26
**PxW7–60**	L Fe-L Zn	23.69	9.41	29.60	23.33	17.87	6.08	37.89	12.62	32.04	10.29	32.99	16.53	32.49	8.88	45.95	9.48
**PxW7-76**	H Zn-H Fe	39.65	12.85	40.56	15.42	24.37	7.45	42.22	10.40	43.24	12.74	39.61	15.44	38.27	12.62	50.98	10.99
**PxW811–10**	H Fe-L Zn	29.75	8.56	31.53	9.12												
**PxW811–30**	H Zn-H Fe	37.57	11.09	35.01	10.80	29.58	7.88	40.38	9.20	42.22	13.20	32.51	14.55	46.57	14.84	44.92	9.55
**PxW811–83**	H Zn-H Fe	36.84	10.20	33.52	12.22	24.29	7.52	33.07	11.06	38.72	11.89	30.58	13.87	33.68	10.13	36.44	9.06

There were positive correlations between the concentration of Fe in wholegrain of the 24 genotypes between Nottingham and Rothamsted sites in 2015–16 (r = 0.80; P<0.001) and 2016–18 (r = 0.51; P<0.05). Within the sites, the mean wholegrain Fe concentrations of 24 genotypes were correlated between 2015–16 and 2016–17 at Nottingham (r = 0.73; P<0.001) and at Rothamsted (r = 0.51; P<0.05). The genotype PxW-76 had the largest wholegrain Fe concentration in 2015–16 at both the Nottingham and Rothamsted sites and the second largest wholegrain Fe concentration in 2016–17 at Nottingham and in 2017–18 at Rothamsted (Table 4).

There were positive correlations between the Zn concentrations in wholegrain and white flour of the 24 genotypes in 2015–16 (r = 0.78; P<0.001) and 2016–17 (r = 0.85; P<0.001) at Nottingham and in 2015–16 (r = 0.79; P<0.001) and 2017–18 (r = 0.94; P<0.001) at Rothamsted. There was also a positive correlation between Zn concentrations in white flour in 2015–16 and 2016–17 at Nottingham (r = 0.86; P<0.0.001) and at Rothamsted (r = 0.54; P<0.05). There were no significant correlations between the Fe concentrations in wholegrain and white flour of the 24 genotypes grown in 2015–16 and 2016–17 at the Nottingham and Rothamsted sites. However, there was a weak positive correlation in the Fe concentrations in white flour in 2015–16 and 2016–17 at Nottingham (r = 0.56; P<0.05) and not at Rothamsted.

Twenty four genotypes showed greater variation in white flour for Zn and Fe concentrations than that in wholegrain at both the Nottingham and Rothamsted sites (Table 4). In particular, the mean Zn concentration of the 24 genotypes, averaged across years, the Zn concentration of high-Zn genotypes was 64% higher in wholegrain and 68% higher in white flour compared to Zn concentration in wholegrain and white flour of the low-Zn genotypes when grown at Nottingham. Similarly, the mean Zn concentration of high-Zn genotypes was 42% higher in wholegrain and 44% higher in white flour compared to Zn concentration in wholegrain and white flour samples of low-Zn genotypes when grown at Rothamsted.

The mean concentration of Fe in the 24 genotypes, averaged across years, the Fe concentration of high-Fe genotypes was 66% higher in wholegrain samples and 77% higher in white flour compared to Fe concentration in wholegrain and white flour of low-Fe genotypes when grown at Nottingham. Similarly, the Fe concentration of the high-Fe genotypes was 34% higher in wholegrain and 49% higher in white flour samples when compared to Fe concentration in wholegrain and white flour samples of low-Fe genotypes grown at Rothamsted (Table 4).

Table 5 shows the concentration of minerals in the wholegrain and white flour of the four groups of genotypes (called groups 1–4) between sites and years. Groups 1, 2 and 4 showed significant differences in Zn concentrations in wholegrain and white flour between sites. Group 3 showed significant differences only in wholegrain Zn concentration between sites not in white flour Zn concentration. There were no significant differences in the Zn concentration in wholegrain and white flour of four different groups between the years (Table 5).

**Table 5:** One way analysis of variance (ANOVA) to study the performance of four groups with different Zn and Fe level combinations in wholegrain and white flour between the sites and years. Each group consist of 6 genotypes and data are 3 replicate plots per genotypes at each site and year.

Levels	Groups	Zn concentration				Fe concentration			
		Site		Year		Site		Year	
		Wholegrain	White flour	Wholegrain	White flour	Wholegrain	White flour	Wholegrain	White flour
**H Zn-H Fe**	1	< .001	< .001	0.003	0.003	0.178	0.714	< .001	< .001
**H Zn-L Fe**	2	< .001	< .001	0.367	0.068	0.046	0.007	< .001	< .001
**L Zn-H Fe**	3	< .001	0.021	0.816	0.096	0.034	0.002	0.081	0.055
**L Zn-L Fe**	4	< .001	< .001	0.09	0.003	< .001	0.008	< .001	< .001

There were no significant differences in the Fe concentrations in wholegrain and white flour of groups 1, 2 and 3 between sites, while group 4 showed significant differences in wholegrain Fe concentration between sites, not in white flour Fe concentration. However, there were significant differences in the Fe concentration of wholegrain and white flour of all groups between the years except group 3 (Table 5).

## Discussion

Despite the presence of high Zn and Fe concentrations in the grain of wheat landraces, only a few studies have exploited the potential of wheat landraces to increase the Zn and Fe levels of modern wheat cultivars [7, 16, 30]. Therefore, our first aim was to screen a panel of 245 wheat genotypes derived from crosses between Watkins landraces and the UK spring wheat cultivar Paragon, for variation in wholegrain Zn and Fe concentrations. The mean wholegrain Zn and Fe concentration in 245 wheat genotypes varied from 24.2 to 48.5 mg kg^-1^ and from 19.4 to 47.7 mg kg^-1^, averaged across sites, respectively. Our results are in line with Guzman *et al*. [16] who reported grain Zn concentration from 19 to 53 mg kg^-1^ and grain Fe concentration 24 to 31 mg kg^-1^ in 35 wheat lines derived from Mexican landraces and grown under field condition. Wholegrain Zn concentration was positively correlated with Fe concentration which is in agreement with Velu *et al*; Khokhar *et al* and Pandey *et al*. [7, 9, 31]. Velu *et al*. [7] reported co-localisation of major QTLs for grain Zn and Fe concentration on 2B chromosomes which may help to simultaneous improvement of both the micronutrients. Grain yield of 245 wheat genotypes showd positive correlation wth TGW which are in agreement with Khokhar *et al*. [32]. Grain protein content of 245 wheat genotypes varied from 12.3 to 18.6% which is in agreement with Dotlacil *et al*. [33] who reported grain protein content from 13 to 18% in 67 wheat lines derived from landraces when evaluated under field conditions in Czech Republic. Grain protein content (GPC) correlated with, both wholegrain Zn and Fe concentrations. Morgounov *et al*. [34] reported positive correlation between grain protein content and grain Zn and Fe concnetrations in 66 wheat varieties evaluated under field conditions in Kazakhstan. High protein content grains accumulate higher Zn and Fe concentrations, it could be due to the co-localisation of protein with grain Zn and Fe concentrations in embryo and aleurone layer of grain [20]. There was a negative correlation between wholegrain Zn concentration and grain yield and grain protein content. Cakmak *et al*. and Oury *et al*. [20, 35] reported lower grain Zn concentration and GPC in high yielding genotypes which may have been due to yield dilution. It is therefore important to continue to monitor grain Zn concentration in the wider context of yield and yield component traits in breeding programmes, especially given that grain yields in the region are relatively low.

A sub-set of 24 genotypes showed strong relationships between the concentrations of Zn and Fe in wholegrain between the years at each site and between the sites. The subset, therefore, performed consistently for wholegrain Zn and Fe concentration between years and sites, indicating the stability of these traits under different growing conditions. However, the concentrations of Zn in wholegrain samples were higher at Rothamsted than at Nottingham in both the years. Grain Zn and Fe concentration are complex traits controlled by many genes and it is therefore not surprising that they are affected by soil and environmental factors. Khokhar *et al* [9] and Velu *et al*. [36] also reported significant effects of the environment on variation in grain Zn and Fe concentrations. Bread wheat is an important dietary source of Zn and Fe globally. Although most wheat products consumed in South Asia are made from high extraction flours (approx. 95% wholegrain), most wheat-based foods consumed in other parts of the world are made from white flour, which corresponds to the starchy endosperm and accounts for about 75–85% of wholegrain [4, 18]. A further aim of the project was therefore to determine the variation in Zn and Fe concentration in wholegrain and white flour of a sub-set of 24 wheat genotypes selected to vary in their levels of both minerals. The contents of Zn in white flours of these genotypes were 56 to 72% lower than in wholegrain, with the concentrations of Fe showing similar differences. This is consistent with both minerals being concentrated in the embryo and aleurone layer of the grain [19, 37, 38], which are removed when milling to produce white flour. Similar differences in the mineral contents of wholegrain and white flours have been reported by Zhang *et al*. [39], Szira *et al*. [40] and Eagling *et al*. [41]. We also showed correlations between the concentrations of Zn in white flour and wholegrain samples, but not between the contents of Fe. This contrasts with Eagling et al. [41] who showed correlations between the contents of Fe in wholegrain and white flour of 6 commercial European genotypes. We have no explanation for this difference.

In conclusion, the present study shows that there is substantial genetic diversity for wholegrain Zn and Fe concentration in a panel of 245 wheat genotypes derived from Watkins landraces and the UK elite cultivar Paragon. This should be available by exploitation by plant breeders, allowing the development of new biofortified cultivars with increased Zn and Fe concentrations in wholegrain and white flour, high grain yields, good agronomic performance, and good processing quality.

## Supporting information

S1 Table Passport data of a panel of 245 wheat genotypes, derived from crosses between landraces from the Watkins collection and the UK cultivar Paragon. (PDF)

S2 Table Primary data of Zn, Fe, GYD, TGW and grain protein content in 245 wheat lines grown at Nottingham and Rothamsted sites in 2015–16. (PDF)

S3 Table Primary data of Zn and Fe concentrations (mg/kg) in wholegrain and white flour of 24 wheat lines grown at Nottingham and Rothamsted sites over two years, 2015–19. (PDF)

## References

[1] Stein AJ, Nestel P, Meenakshi JV, Qaim M, Sachdev HP, Bhutta ZA. Plant breeding to control zinc deficiency in India: how cost-effective is biofortification? Public Health Nutrition. 2007;10(5):492–501. Epub 2007/04/07. 10.1017/S1368980007223857 . 17411470

[2] Hess SY, King JC. Effects of maternal zinc supplementation on pregnancy and lactation outcomes. Food Nutr Bull. 2009;30(1 Suppl):S60–78. 10.1177/15648265090301S105 . 19472602

[3] Black RE, Victora CG, Walker SP, Bhutta ZA, Christian P, de Onis M, et al Maternal and child undernutrition and overweight in low-income and middle-income countries. Lancet. 2013;382(9890):427–51. 10.1016/S0140-6736(13)60937-X PubMed PMID: WOS:000322638500036. 23746772

[4] Kumssa DB, Joy EJ, Ander EL, Watts MJ, Young SD, Walker S, et al Dietary calcium and zinc deficiency risks are decreasing but remain prevalent. Sci Rep. 2015;5:10974 10.1038/srep10974 26098577 PMC4476434

[5] Lockyer S, White A, Walton J, Buttriss JL. Proceedings of the ‘Working together to consider the role of biofortification in the global food chain’ workshop. Nutrition Bulletin. 2018;43(4):416–27. 10.1111/nbu.12348

[6] Roberts C, Steer T, Maplethorpe N, Cox L, Meadows S, Nicholson S, et al National Diet and Nutrotion Survey Results from Years 7 and 8 (combined) of the Rolling Programme (2014/2015–2015/2016). 2018.

[7] Velu G, Tutus Y, Gomez-Becerra HF, Hao YF, Demir L, Kara R, et al QTL mapping for grain zinc and iron concentrations and zinc efficiency in a tetraploid and hexaploid wheat mapping populations. Plant Soil. 2017;411(1–2):81–99. 10.1007/s11104-016-3025-8 PubMed PMID: WOS:000394142900007.

[8] Chen XP, Zhang YQ, Tong YP, Xue YF, Liu DY, Zhang W, et al Harvesting more grain zinc of wheat for human health. Sci Rep. 2017;7(1):7016 10.1038/s41598-017-07484-2 28765540 PMC5539200

[9] Khokhar JS, Sareen S, Tyagi BS, Singh G, Wilson L, King IP, et al Variation in grain Zn concentration, and the grain ionome, in field-grown Indian wheat. PLoS One. 2018;13(1):e0192026 10.1371/journal.pone.0192026 29381740 PMC5790267

[10] Joy EJM, Stein AJ, Young SD, Ander EL, Watts MJ, Broadley MR. Zinc-enriched fertilisers as a potential public health intervention in Africa. Plant and Soil. 2015;389(1–2):1–24. 10.1007/s11104-015-2430-8 PubMed PMID: WOS:000352153600001.

[11] Cakmak I, Kutman UB. Agronomic biofortification of cereals with zinc: a review. Eur J Soil Sci. 2018;69(1):172–80. 10.1111/ejss.12437 PubMed PMID: WOS:000427881900021.

[12] Sazawal S, Dhingra U, Dhingra P, Dutta A, Deb S, Kumar J, et al Efficacy of high zinc biofortified wheat in improvement of micronutrient status, and prevention of morbidity among preschool children and women—a double masked, randomized, controlled trial. Nutrition Journal. 2018;17(1):86 10.1186/s12937-018-0391-5 30219062 PMC6139156

[13] Bouis HE, Saltzman A. Improving nutrition through biofortification: A review of evidence from HarvestPlus, 2003 through 2016. Global Food Security. 2017;12(Supplement C):49–58. 10.1016/j.gfs.2017.01.009 28580239 PMC5439484

[14] Singh R, Velu G. Zinc-biofortified wheat: harnessing genetic diversity for improved nutritional quality. CIMMYT, HarvestPlus, and the Global Crop Diversity Trust 2017 May, 2017. Report No.

[15] Cakmak I, Torun A, Millet E, Feldman M, Fahima T, Korol A, et al Triticum dicoccoides: An important genetic resource for increasing zinc and iron concentration in modern cultivated wheat. Soil Science and Plant Nutrition. 2004;50(7):1047–54. 10.1080/00380768.2004.10408573 PubMed PMID: WOS:000225652800015.

[16] Guzman C, Medina-Larque AS, Velu G, Gonzalez-Santoyo H, Singh RP, Huerta-Espino J, et al Use of wheat genetic resources to develop biofortified wheat with enhanced grain zinc and iron concentrations and desirable processing quality. J Cereal Sci. 2014;60(3):617–22. 10.1016/j.jcs.2014.07.006 PubMed PMID: WOS:000347592700023.

[17] Srinivasa J, Arun B, Mishra VK, Singh GP, Velu G, Babu R, et al Zinc and iron concentration QTL mapped in a Triticum spelta x T. aestivum cross. Theor Appl Genet. 2014;127(7):1643–51. Epub 2014/05/29. 10.1007/s00122-014-2327-6 . 24865507

[18] Pena-Bautista RJ, Hernandez-Espinosa N, Jones JM, Guzman C, Braun HJ. CIMMYT Series on Carbohydrates, Wheat, Grains, and Health Wheat-Based Foods: Their Global and Regional Importance in the Food Supply, Nutrition, and Health. Cereal Foods World. 2017;62(5):231–49. 10.1094/Cfw-62-5-0231 PubMed PMID: WOS:000413758800009.

[19] Ozturk L, Yazici MA, Yucel C, Torun A, Cekic C, Bagci A, et al Concentration and localization of zinc during seed development and germination in wheat. Physiol Plant. 2006;128(1):144–52. 10.1111/j.1399-3054.2006.00737.x PubMed PMID: WOS:000240335000015.

[20] Cakmak I, Kalayci M, Kaya Y, Torun AA, Aydin N, Wang Y, et al Biofortification and localization of zinc in wheat grain. J Agric Food Chem. 2010;58(16):9092–102. 10.1021/jf101197h . 23654236

[21] Ajiboye B, Cakmak I, Paterson D, de Jonge MD, Howard DL, Stacey SP, et al X-ray fluorescence microscopy of zinc localization in wheat grains biofortified through foliar zinc applications at different growth stages under field conditions. Plant Soil. 2015;392(1–2):357–70. 10.1007/s11104-015-2467-8 PubMed PMID: WOS:000355152300030.

[22] Gibson RS, Bailey KB, Gibbs M, Ferguson EL. A review of phytate, iron, zinc, and calcium concentrations in plant-based complementary foods used in low-income countries and implications for bioavailability. Food Nutr Bull. 2010;31(2 Suppl):S134–46. 10.1177/15648265100312S206 . 20715598

[23] Gupta RK, Gangoliya SS, Singh NK. Reduction of phytic acid and enhancement of bioavailable micronutrients in food grains. J Food Sci Technol. 2015;52(2):676–84. 10.1007/s13197-013-0978-y 25694676 PMC4325021

[24] Wingen LU, Orford S, Goram R, Leverington-Waite M, Bilham L, Patsiou TS, et al Establishing the A. E. Watkins landrace cultivar collection as a resource for systematic gene discovery in bread wheat. Theor Appl Genet. 2014;127(8):1831–42. 10.1007/s00122-014-2344-5 24985064 PMC4110413

[25] Wingen LU, West C, Leverington-Waite M, Collier S, Orford S, Goram R, et al Wheat Landrace Genome Diversity. Genetics. 2017;205(4):1657–76. 10.1534/genetics.116.194688 28213475 PMC5378120

[26] Thomas CL, Alcock TD, Graham NS, Hayden R, Matterson S, Wilson L, et al Root morphology and seed and leaf ionomic traits in a Brassica napus L. diversity panel show wide phenotypic variation and are characteristic of crop habit. BMC Plant Biol. 2016;16(1):214 10.1186/s12870-016-0902-5 27716103 PMC5050600

[27] Kjeldahl J. New methods for the determination of nitrogen. Chem News 1240. 1883;101–102.

[28] Cataldo DA, Haroon M, Schrader LE, Youngs VL. Rapid colorimetric determination of nitrate in plant-tissue by nitration of salicylic acid. Commun Soil Sci Plant Analy. 1975: 6:71–80.

[29] AACCI. Approved methods of the American association of cereal chemists. Amer Assn Cereal Chem. 2000;1.

[30] Badakhshan H, Moradi N, Mohammadzadeh H, Zakeri MR. Genetic variability analysis of grains Fe, Zn and beta-carotene concentration of prevalent wheat varieties in Iran. International Journal of Agriculture and Crop Sciences. 2013;6(2):57.

[31] Pandey A, Khan MK, Hakki EE, Thomas G, Hamurcu M, Gezgin S, et al Assessment of genetic variability for grain nutrients from diverse regions: potential for wheat improvement. Springerplus. 2016;5(1):1912 10.1186/s40064-016-3586-2 27867819 PMC5095102

[32] Khokhar JS, Sareen S, Tyagi BS, Singh G, Chowdhury AK, Dhar T, et al Characterising variation in wheat traits under hostile soil conditions in India. PLoS One. 2017;12(6): 10.1371/journal.pone.0179208 PMC5467898 28604800

[33] DoTLačiL L, HermuTH J. STeHno Z, DVořáček V, BraDoVá J and LeišoVa L. How can wheat landraces contribute to present breeding?. Czech J. Genet. Plant Breed. 2010; 46, (Special Issue): S70–S74.

[34] Morgounov A, Gomez-Becerra HF, Abugalieva A, Dzhunusova M, Yessimbekova M, Muminjanov H. et al Iron and zinc grain density in common wheat grown in Central Asia. Euphytica. 155, 193–203

[35] Oury FX, Leenhardt F, Remesy C, Chanliaud E, Duperrier B, Balfourier F, et al Genetic variability and stability of grain magnesium, zinc and iron concentrations in bread wheat. Eur J Agron. 2006;25(2):177–85. 10.1016/j.eja.2006.04.011 PubMed PMID: WOS:000239951700012.

[36] Velu G, Singh RP, Huerta-Espino J, Pena RJ, Arun B, Mahendru-Singh A, et al Performance of biofortified spring wheat genotypes in target environments for grain zinc and iron concentrations. Field Crop Res. 2012;137:261–7. 10.1016/j.fcr.2012.07.018 PubMed PMID: WOS:000312465600030.

[37] Borg S, Brinch-Pedersen H, Tauris B, Holm PB. Iron transport, deposition and bioavailability in the wheat and barley grain. Plant Soil. 2009;325(1–2):15–24. 10.1007/s11104-009-0046-6 PubMed PMID: WOS:000272383900003.

[38] Stomph TJ, Choi EY, Stangoulis JC. Temporal dynamics in wheat grain zinc distribution: is sink limitation the key? Ann Bot. 2011;107(6):927–37. Epub 2011/03/10. 10.1093/aob/mcr040 21385780 PMC3080623

[39] Zhang Y, Shi R, Rezaul KM, Zhang F, Zou C. Iron and zinc concentrations in grain and flour of winter wheat as affected by foliar application. J Agric Food Chem. 2010;58(23):12268–74. Epub 2010/11/16. 10.1021/jf103039k . 21073194

[40] Szira F, Monostori I, Galiba G, Rakszegi M, Bálint AF. Micronutrient contents and nutritional values of commercial wheat flours and flours of field-grown wheat varieties—A survey in Hungary. Cereal Res Commun. 2014;42(2):293–302. 10.1556/crc.2013.0059

[41] Eagling T, Neal AL, McGrath SP, Fairweather-Tait S, Shewry PR, Zhao FJ. Distribution and speciation of iron and zinc in grain of two wheat genotypes. J Agric Food Chem. 2014;62(3):708–16. 10.1021/jf403331p . 24382168

