# Supplementary material for: Novel sources of variation in grain Zinc (Zn) concentration in bread wheat germplasm derived from Watkins landraces
Source: PLoS One. 2020 Feb 28;15(2):e0229107. doi: 10.1371/journal.pone.0229107 (PMC7048275; doi:10.1371/journal.pone.0229107)
Supplement: S1 Table — (PDF) [file pone.0229107.s001.pdf]

Supplementary Table 1. Passport data of 245 wheat lines derived from Watkins landraces and

Sr No	Genotype	Watkin_accession_No	Watkin_Country_Origin
1	PxW7 - 2	1190007	Australia
2	PxW7 - 3	1190007	Australia
3	PxW7 - 15	1190007	Australia
4	PxW7 - 18	1190007	Australia
5	PxW7 - 29	1190007	Australia
6	PxW7 - 32	1190007	Australia
7	PxW7 - 47	1190007	Australia
8	PxW7 - 60	1190007	Australia
9	PxW7 - 71	1190007	Australia
10	PxW7 - 76	1190007	Australia
11	PxW7 - 77	1190007	Australia
12	PxW7 - 87	1190007	Australia
13	PxW32 - 8	1190032	India
14	PxW32 - 27	1190032	India
15	PxW32 - 29	1190032	India
16	PxW32 - 50	1190032	India
17	PxW32 - 56	1190032	India
18	PxW32 - 57	1190032	India
19	PxW32 - 60	1190032	India
20	PxW32 - 61	1190032	India
21	PxW32 - 62	1190032	India
22	PxW32 - 66	1190032	India
23	PxW32 - 76	1190032	India
24	PxW32 - 81	1190032	India
25	PxW42 - 14	1190042	France
26	PxW42 - 17	1190042	France
27	PxW42 - 18	1190042	France
28	PxW42 - 22	1190042	France
29	PxW42 - 33	1190042	France
30	PxW42 - 34	1190042	France
31	PxW42 - 45	1190042	France
32	PxW42 - 60	1190042	France
33	PxW42 - 68	1190042	France
34	PxW42 - 75	1190042	France
35	PxW42 - 90	1190042	France
36	PxW42 - 91	1190042	France
37	PxW42 - 93	1190042	France
38	PxW216 - 3	1190216	Morocco
39	PxW216 - 4	1190216	Morocco
40	PxW216 - 30	1190216	Morocco
41	PxW216 - 45	1190216	Morocco
42	PxW216 - 48	1190216	Morocco
43	PxW216 - 74	1190216	Morocco
44	PxW216 - 76	1190216	Morocco
45	PxW216 - 81	1190216	Morocco
46	PxW216 - 85	1190216	Morocco
47	PxW216 - 88	1190216	Morocco
48	PxW216 - 89	1190216	Morocco

49	PxW216 - 92	1190216	Morocco
50	PxW216 - 94	1190216	Morocco
51	PxW223 - 1	1190223	Burma
52	PxW223 - 2	1190223	Burma
53	PxW223 - 3	1190223	Burma
54	PxW223 - 25	1190223	Burma
55	PxW223 - 80	1190223	Burma
56	PxW223 - 83	1190223	Burma
57	PxW223 - 85	1190223	Burma
58	PxW223 - 86	1190223	Burma
59	PxW223 - 89	1190223	Burma
60	PxW223 - 90	1190223	Burma
61	PxW223 - 91	1190223	Burma
62	PxW223 - 92	1190223	Burma
63	PxW223 - 94	1190223	Burma
64	PxW254 - 2	1190254	Morocco
65	PxW254 - 3	1190254	Morocco
66	PxW254 - 24	1190254	Morocco
67	PxW254 - 39	1190254	Morocco
68	PxW254 - 40	1190254	Morocco
69	PxW254 - 52	1190254	Morocco
70	PxW254 - 55	1190254	Morocco
71	PxW254 - 59	1190254	Morocco
72	PxW254 - 69	1190254	Morocco
73	PxW254 - 74	1190254	Morocco
74	PxW254 - 76	1190254	Morocco
75	PxW254 - 84	1190254	Morocco
76	PxW254 - 87	1190254	Morocco
77	PxW264 - 9	1190264	Canary Islands
78	PxW264 - 10	1190264	Canary Islands
79	PxW264 - 12	1190264	Canary Islands
80	PxW264 - 16	1190264	Canary Islands
81	PxW264 - 17	1190264	Canary Islands
82	PxW264 - 31	1190264	Canary Islands
83	PxW264 - 33	1190264	Canary Islands
84	PxW264 - 41	1190264	Canary Islands
85	PxW264 - 47	1190264	Canary Islands
86	PxW264 - 50	1190264	Canary Islands
87	PxW264 - 51	1190264	Canary Islands
88	PxW264 - 52	1190264	Canary Islands
89	PxW264 - 86	1190264	Canary Islands
90	PxW273 - 11	1190273	Spain
91	PxW273 - 15	1190273	Spain
92	PxW273 - 19	1190273	Spain
93	PxW273 - 21	1190273	Spain
94	PxW273 - 26	1190273	Spain
95	PxW273 - 35	1190273	Spain
96	PxW273 - 45	1190273	Spain
97	PxW273 - 52	1190273	Spain
98	PxW273 - 58	1190273	Spain

99	PxW273 - 71	1190273	Spain
100	PxW273 - 79	1190273	Spain
101	PxW273 - 81	1190273	Spain
102	PxW273 - 87	1190273	Spain
103	PxW291 - 8	1190291	Cyprus
104	PxW291 - 12	1190291	Cyprus
105	PxW291 - 13	1190291	Cyprus
106	PxW291 - 23	1190291	Cyprus
107	PxW291 - 25	1190291	Cyprus
108	PxW291 - 35	1190291	Cyprus
109	PxW291 - 39	1190291	Cyprus
110	PxW291 - 45	1190291	Cyprus
111	PxW291 - 47	1190291	Cyprus
112	PxW291 - 50	1190291	Cyprus
113	PxW291 - 51	1190291	Cyprus
114	PxW291 - 74	1190291	Cyprus
115	PxW291 - 75	1190291	Cyprus
116	PxW299 - 14	1190299	Turkey
117	PxW299 - 17	1190299	Turkey
118	PxW299 - 20	1190299	Turkey
119	PxW299 - 31	1190299	Turkey
120	PxW299 - 34	1190299	Turkey
121	PxW299 - 40	1190299	Turkey
122	PxW299 - 47	1190299	Turkey
123	PxW299 - 51	1190299	Turkey
124	PxW299 - 63	1190299	Turkey
125	PxW299 - 68	1190299	Turkey
126	PxW299 - 69	1190299	Turkey
127	PxW299 - 78	1190299	Turkey
128	PxW299 - 87	1190299	Turkey
129	PxW349 - 7	1190349	Bulgaria
130	PxW349 - 10	1190349	Bulgaria
131	PxW349 - 16	1190349	Bulgaria
132	PxW349 - 19	1190349	Bulgaria
133	PxW349 - 22	1190349	Bulgaria
134	PxW349 - 28	1190349	Bulgaria
135	PxW349 - 31	1190349	Bulgaria
136	PxW349 - 42	1190349	Bulgaria
137	PxW349 - 46	1190349	Bulgaria
138	PxW349 - 58	1190349	Bulgaria
139	PxW349 - 65	1190349	Bulgaria
140	PxW349 - 66	1190349	Bulgaria
141	PxW349 - 72	1190349	Bulgaria
142	PxW396 - 5	1190396	Portugal
143	PxW396 - 12	1190396	Portugal
144	PxW396 - 19	1190396	Portugal
145	PxW396 - 30	1190396	Portugal
146	PxW396 - 37	1190396	Portugal
147	PxW396 - 46	1190396	Portugal
148	PxW396 - 48	1190396	Portugal

149	PxW396 - 49	1190396	Portugal
150	PxW396 - 51	1190396	Portugal
151	PxW396 - 56	1190396	Portugal
152	PxW396 - 60	1190396	Portugal
153	PxW396 - 62	1190396	Portugal
154	PxW396 - 73	1190396	Portugal
155	PxW397 - 20	1190397	Portugal
156	PxW397 - 26	1190397	Portugal
157	PxW397 - 33	1190397	Portugal
158	PxW397 - 48	1190397	Portugal
159	PxW397 - 50	1190397	Portugal
160	PxW397 - 51	1190397	Portugal
161	PxW397 - 61	1190397	Portugal
162	PxW397 - 76	1190397	Portugal
163	PxW397 - 80	1190397	Portugal
164	PxW397 - 82	1190397	Portugal
165	PxW397 - 83	1190397	Portugal
166	PxW397 - 88	1190397	Portugal
167	PxW397 - 89	1190397	Portugal
168	PxW398 - 18	1190398	Palestine
169	PxW398 - 21	1190398	Palestine
170	PxW398 - 41	1190398	Palestine
171	PxW398 - 42	1190398	Palestine
172	PxW398 - 44	1190398	Palestine
173	PxW398 - 49	1190398	Palestine
174	PxW398 - 55	1190398	Palestine
175	PxW398 - 56	1190398	Palestine
176	PxW398 - 60	1190398	Palestine
177	PxW398 - 63	1190398	Palestine
178	PxW398 - 74	1190398	Palestine
179	PxW398 - 81	1190398	Palestine
180	PxW398 - 85	1190398	Palestine
181	PxW420 - 1	1190420	India
182	PxW420 - 3	1190420	India
183	PxW420 - 8	1190420	India
184	PxW420 - 10	1190420	India
185	PxW420 - 11	1190420	India
186	PxW420 - 21	1190420	India
187	PxW420 - 22	1190420	India
188	PxW420 - 25	1190420	India
189	PxW420 - 31	1190420	India
190	PxW420 - 32	1190420	India
191	PxW420 - 37	1190420	India
192	PxW420 - 67	1190420	India
193	PxW420 - 94	1190420	India
194	PxW546 - 3	1190546	Spain
195	PxW546 - 8	1190546	Spain
196	PxW546 - 12	1190546	Spain
197	PxW546 - 15	1190546	Spain
198	PxW546 - 16	1190546	Spain

199	PxW546 - 20	1190546	Spain
200	PxW546 - 24	1190546	Spain
201	PxW546 - 25	1190546	Spain
202	PxW546 - 27	1190546	Spain
203	PxW546 - 29	1190546	Spain
204	PxW546 - 32	1190546	Spain
205	PxW546 - 38	1190546	Spain
206	PxW546 - 47	1190546	Spain
207	PxW566 - 7	1190566	Greece
208	PxW566 - 12	1190566	Greece
209	PxW566 - 14	1190566	Greece
210	PxW566 - 17	1190566	Greece
211	PxW566 - 20	1190566	Greece
212	PxW566 - 21	1190566	Greece
213	PxW566 - 23	1190566	Greece
214	PxW566 - 24	1190566	Greece
215	PxW566 - 35	1190566	Greece
216	PxW566 - 50	1190566	Greece
217	PxW566 - 52	1190566	Greece
218	PxW566 - 72	1190566	Greece
219	PxW566 - 92	1190566	Greece
220	PxW685 - 1	1190685	Spain
221	PxW685 - 6	1190685	Spain
222	PxW685 - 7	1190685	Spain
223	PxW685 - 9	1190685	Spain
224	PxW685 - 12	1190685	Spain
225	PxW685 - 16	1190685	Spain
226	PxW685 - 22	1190685	Spain
227	PxW685 - 36	1190685	Spain
228	PxW685 - 41	1190685	Spain
229	PxW685 - 44	1190685	Spain
230	PxW685 - 55	1190685	Spain
231	PxW685 - 80	1190685	Spain
232	PxW685 - 88	1190685	Spain
233	PxW811 - 10	1190811	Tunisia
234	PxW811 - 26	1190811	Tunisia
235	PxW811 - 28	1190811	Tunisia
236	PxW811 - 30	1190811	Tunisia
237	PxW811 - 42	1190811	Tunisia
238	PxW811 - 50	1190811	Tunisia
239	PxW811 - 56	1190811	Tunisia
240	PxW811 - 59	1190811	Tunisia
241	PxW811 - 60	1190811	Tunisia
242	PxW811 - 83	1190811	Tunisia
243	PxW811 - 84	1190811	Tunisia
244	PxW811 - 90	1190811	Tunisia
245	PxW811 - 96	1190811	Tunisia

ıd a wheat cultivar "Paragon'
