# Supplementary material for: Novel sources of variation in grain Zinc (Zn) concentration in bread wheat germplasm derived from Watkins landraces
Source: PLoS One. 2020 Feb 28;15(2):e0229107. doi: 10.1371/journal.pone.0229107 (PMC7048275; doi:10.1371/journal.pone.0229107)
Supplement: S2 Table — (PDF) [file pone.0229107.s002.pdf]

Supplementary Table 2. Primary data of 245 Watkins and Paragon derived wheat lines for grain Zn, Fe, GYD, TGW and grain protein conten, grown at Nottingham ar

Sr No!	Plot No!	Replication!	Treat!	Genotype!	Watkin_accession_No!	Watkin_Country_Origin!	Site!
247	247	3	115	PxW7 - 2	1190007	Australia	UoN
684	684	2	115	PxW7 - 2	1190007	Australia	UoN
1013	1013	1	115	PxW7 - 2	1190007	Australia	UoN
5	5	3	116	PxW7 - 3	1190007	Australia	UoN
544	544	1	116	PxW7 - 3	1190007	Australia	UoN
744	744	2	116	PxW7 - 3	1190007	Australia	UoN
241	241	3	117	PxW7 - 15	1190007	Australia	UoN
668	668	1	117	PxW7 - 15	1190007	Australia	UoN
1042	1042	2	117	PxW7 - 15	1190007	Australia	UoN
115	115	3	118	PxW7 - 18	1190007	Australia	UoN
647	647	1	118	PxW7 - 18	1190007	Australia	UoN
818	818	2	118	PxW7 - 18	1190007	Australia	UoN
112	112	3	119	PxW7 - 29	1190007	Australia	UoN
460	460	2	119	PxW7 - 29	1190007	Australia	UoN
1017	1017	1	119	PxW7 - 29	1190007	Australia	UoN
708	708	3	120	PxW7 - 32	1190007	Australia	UoN
765	765	1	120	PxW7 - 32	1190007	Australia	UoN
1176	1176	2	120	PxW7 - 32	1190007	Australia	UoN
221	221	3	121	PxW7 - 47	1190007	Australia	UoN
537	537	1	121	PxW7 - 47	1190007	Australia	UoN
1105	1105	2	121	PxW7 - 47	1190007	Australia	UoN
225	225	3	122	PxW7 - 60	1190007	Australia	UoN
633	633	2	122	PxW7 - 60	1190007	Australia	UoN
915	915	1	122	PxW7 - 60	1190007	Australia	UoN
376	376	3	123	PxW7 - 71	1190007	Australia	UoN
432	432	1	123	PxW7 - 71	1190007	Australia	UoN
867	867	2	123	PxW7 - 71	1190007	Australia	UoN
425	425	1	124	PxW7 - 76	1190007	Australia	UoN
822	822	3	124	PxW7 - 76	1190007	Australia	UoN
869	869	2	124	PxW7 - 76	1190007	Australia	UoN
231	231	3	125	PxW7 - 77	1190007	Australia	UoN

690	690	2	125	PxW7 - 77	1190007	Australia	UoN
1152	1152	1	125	PxW7 - 77	1190007	Australia	UoN
449	449	2	126	PxW7 - 87	1190007	Australia	UoN
583	583	3	126	PxW7 - 87	1190007	Australia	UoN
1003	1003	1	126	PxW7 - 87	1190007	Australia	UoN
249	249	3	127	PxW32 - 8	1190032	India	UoN
863	863	2	127	PxW32 - 8	1190032	India	UoN
897	897	1	127	PxW32 - 8	1190032	India	UoN
101	101	3	128	PxW32 - 27	1190032	India	UoN
526	526	1	128	PxW32 - 27	1190032	India	UoN
880	880	2	128	PxW32 - 27	1190032	India	UoN
12	12	3	129	PxW32 - 29	1190032	India	UoN
548	548	1	129	PxW32 - 29	1190032	India	UoN
997	997	2	129	PxW32 - 29	1190032	India	UoN
407	407	1	130	PxW32 - 50	1190032	India	UoN
581	581	3	130	PxW32 - 50	1190032	India	UoN
923	923	2	130	PxW32 - 50	1190032	India	UoN
525	525	1	131	PxW32 - 56	1190032	India	UoN
692	692	2	131	PxW32 - 56	1190032	India	UoN
858	858	3	131	PxW32 - 56	1190032	India	UoN
19	19	3	132	PxW32 - 57	1190032	India	UoN
627	627	2	132	PxW32 - 57	1190032	India	UoN
642	642	1	132	PxW32 - 57	1190032	India	UoN
464	464	3	133	PxW32 - 60	1190032	India	UoN
533	533	1	133	PxW32 - 60	1190032	India	UoN
929	929	2	133	PxW32 - 60	1190032	India	UoN
392	392	2	134	PxW32 - 61	1190032	India	UoN
466	466	3	134	PxW32 - 61	1190032	India	UoN
523	523	1	134	PxW32 - 61	1190032	India	UoN
104	104	3	135	PxW32 - 62	1190032	India	UoN
756	756	2	135	PxW32 - 62	1190032	India	UoN
919	919	1	135	PxW32 - 62	1190032	India	UoN
255	255	3	136	PxW32 - 66	1190032	India	UoN

799	799	1	136	PxW32 - 66	1190032	India	UoN
998	998	2	136	PxW32 - 66	1190032	India	UoN
117	117	3	137	PxW32 - 76	1190032	India	UoN
334	334	2	137	PxW32 - 76	1190032	India	UoN
552	552	1	137	PxW32 - 76	1190032	India	UoN
387	387	2	138	PxW32 - 81	1190032	India	UoN
405	405	1	138	PxW32 - 81	1190032	India	UoN
707	707	3	138	PxW32 - 81	1190032	India	UoN
480	480	3	139	PxW42 - 14	1190042	France	UoN
1010	1010	1	139	PxW42 - 14	1190042	France	UoN
1174	1174	2	139	PxW42 - 14	1190042	France	UoN
107	107	3	140	PxW42 - 17	1190042	France	UoN
427	427	1	140	PxW42 - 17	1190042	France	UoN
926	926	2	140	PxW42 - 17	1190042	France	UoN
234	234	3	141	PxW42 - 18	1190042	France	UoN
866	866	2	141	PxW42 - 18	1190042	France	UoN
1036	1036	1	141	PxW42 - 18	1190042	France	UoN
484	484	3	142	PxW42 - 22	1190042	France	UoN
539	539	1	142	PxW42 - 22	1190042	France	UoN
874	874	2	142	PxW42 - 22	1190042	France	UoN
398	398	2	143	PxW42 - 33	1190042	France	UoN
592	592	3	143	PxW42 - 33	1190042	France	UoN
1134	1134	1	143	PxW42 - 33	1190042	France	UoN
462	462	3	144	PxW42 - 34	1190042	France	UoN
1121	1121	1	144	PxW42 - 34	1190042	France	UoN
1171	1171	2	144	PxW42 - 34	1190042	France	UoN
472	472	3	145	PxW42 - 45	1190042	France	UoN
546	546	1	145	PxW42 - 45	1190042	France	UoN
1053	1053	2	145	PxW42 - 45	1190042	France	UoN
124	124	3	146	PxW42 - 60	1190042	France	UoN
901	901	1	146	PxW42 - 60	1190042	France	UoN
1161	1161	2	146	PxW42 - 60	1190042	France	UoN
423	423	1	147	PxW42 - 68	1190042	France	UoN

609	609	3	147	PxW42 - 68	1190042	France	UoN
1059	1059	2	147	PxW42 - 68	1190042	France	UoN
120	120	3	148	PxW42 - 75	1190042	France	UoN
670	670	1	148	PxW42 - 75	1190042	France	UoN
689	689	2	148	PxW42 - 75	1190042	France	UoN
122	122	3	149	PxW42 - 90	1190042	France	UoN
515	515	2	149	PxW42 - 90	1190042	France	UoN
1032	1032	1	149	PxW42 - 90	1190042	France	UoN
498	498	3	150	PxW42 - 91	1190042	France	UoN
504	504	2	150	PxW42 - 91	1190042	France	UoN
781	781	1	150	PxW42 - 91	1190042	France	UoN
13	13	3	151	PxW42 - 93	1190042	France	UoN
576	576	2	151	PxW42 - 93	1190042	France	UoN
658	658	1	151	PxW42 - 93	1190042	France	UoN
379	379	3	152	PxW216 - 3	1190216	Morocco	UoN
982	982	2	152	PxW216 - 3	1190216	Morocco	UoN
1020	1020	1	152	PxW216 - 3	1190216	Morocco	UoN
377	377	3	153	PxW216 - 4	1190216	Morocco	UoN
446	446	2	153	PxW216 - 4	1190216	Morocco	UoN
917	917	1	153	PxW216 - 4	1190216	Morocco	UoN
304	304	1	154	PxW216 - 30	1190216	Morocco	UoN
691	691	2	154	PxW216 - 30	1190216	Morocco	UoN
832	832	3	154	PxW216 - 30	1190216	Morocco	UoN
440	440	1	155	PxW216 - 45	1190216	Morocco	UoN
451	451	2	155	PxW216 - 45	1190216	Morocco	UoN
729	729	3	155	PxW216 - 45	1190216	Morocco	UoN
589	589	3	156	PxW216 - 48	1190216	Morocco	UoN
648	648	1	156	PxW216 - 48	1190216	Morocco	UoN
1060	1060	2	156	PxW216 - 48	1190216	Morocco	UoN
243	243	3	157	PxW216 - 74	1190216	Morocco	UoN
764	764	1	157	PxW216 - 74	1190216	Morocco	UoN
1050	1050	2	157	PxW216 - 74	1190216	Morocco	UoN
728	728	3	158	PxW216 - 76	1190216	Morocco	UoN

748	748	2	158	PxW216 - 76	1190216	Morocco	UoN
1040	1040	1	158	PxW216 - 76	1190216	Morocco	UoN
227	227	3	159	PxW216 - 81	1190216	Morocco	UoN
775	775	1	159	PxW216 - 81	1190216	Morocco	UoN
925	925	2	159	PxW216 - 81	1190216	Morocco	UoN
371	371	3	160	PxW216 - 85	1190216	Morocco	UoN
791	791	1	160	PxW216 - 85	1190216	Morocco	UoN
1179	1179	2	160	PxW216 - 85	1190216	Morocco	UoN
402	402	1	161	PxW216 - 88	1190216	Morocco	UoN
754	754	2	161	PxW216 - 88	1190216	Morocco	UoN
843	843	3	161	PxW216 - 88	1190216	Morocco	UoN
252	252	3	162	PxW216 - 89	1190216	Morocco	UoN
542	542	1	162	PxW216 - 89	1190216	Morocco	UoN
1168	1168	2	162	PxW216 - 89	1190216	Morocco	UoN
108	108	3	163	PxW216 - 92	1190216	Morocco	UoN
773	773	1	163	PxW216 - 92	1190216	Morocco	UoN
817	817	2	163	PxW216 - 92	1190216	Morocco	UoN
245	245	3	164	PxW216 - 94	1190216	Morocco	UoN
646	646	1	164	PxW216 - 94	1190216	Morocco	UoN
1108	1108	2	164	PxW216 - 94	1190216	Morocco	UoN
132	132	3	165	PxW223 - 1	1190223	Burma	UoN
699	699	2	165	PxW223 - 1	1190223	Burma	UoN
766	766	1	165	PxW223 - 1	1190223	Burma	UoN
796	796	1	166	PxW223 - 2	1190223	Burma	UoN
838	838	3	166	PxW223 - 2	1190223	Burma	UoN
1167	1167	2	166	PxW223 - 2	1190223	Burma	UoN
410	410	1	167	PxW223 - 3	1190223	Burma	UoN
597	597	3	167	PxW223 - 3	1190223	Burma	UoN
983	983	2	167	PxW223 - 3	1190223	Burma	UoN
332	332	2	168	PxW223 - 25	1190223	Burma	UoN
474	474	3	168	PxW223 - 25	1190223	Burma	UoN
913	913	1	168	PxW223 - 25	1190223	Burma	UoN
411	411	1	169	PxW223 - 80	1190223	Burma	UoN

562	562	2	169	PxW223 - 80	1190223	Burma	UoN
727	727	3	169	PxW223 - 80	1190223	Burma	UoN
381	381	2	170	PxW223 - 83	1190223	Burma	UoN
478	478	3	170	PxW223 - 83	1190223	Burma	UoN
798	798	1	170	PxW223 - 83	1190223	Burma	UoN
314	314	1	171	PxW223 - 85	1190223	Burma	UoN
620	620	3	171	PxW223 - 85	1190223	Burma	UoN
1175	1175	2	171	PxW223 - 85	1190223	Burma	UoN
312	312	1	172	PxW223 - 86	1190223	Burma	UoN
626	626	2	172	PxW223 - 86	1190223	Burma	UoN
739	739	3	172	PxW223 - 86	1190223	Burma	UoN
362	362	3	173	PxW223 - 89	1190223	Burma	UoN
760	760	2	173	PxW223 - 89	1190223	Burma	UoN
1130	1130	1	173	PxW223 - 89	1190223	Burma	UoN
125	125	3	174	PxW223 - 90	1190223	Burma	UoN
400	400	2	174	PxW223 - 90	1190223	Burma	UoN
651	651	1	174	PxW223 - 90	1190223	Burma	UoN
348	348	3	175	PxW223 - 91	1190223	Burma	UoN
669	669	1	175	PxW223 - 91	1190223	Burma	UoN
1163	1163	2	175	PxW223 - 91	1190223	Burma	UoN
375	375	3	176	PxW223 - 92	1190223	Burma	UoN
899	899	1	176	PxW223 - 92	1190223	Burma	UoN
1177	1177	2	176	PxW223 - 92	1190223	Burma	UoN
250	250	3	177	PxW223 - 94	1190223	Burma	UoN
635	635	2	177	PxW223 - 94	1190223	Burma	UoN
1016	1016	1	177	PxW223 - 94	1190223	Burma	UoN
18	18	3	178	PxW254 - 2	1190254	Morocco	UoN
667	667	1	178	PxW254 - 2	1190254	Morocco	UoN
747	747	2	178	PxW254 - 2	1190254	Morocco	UoN
536	536	1	179	PxW254 - 3	1190254	Morocco	UoN
574	574	2	179	PxW254 - 3	1190254	Morocco	UoN
740	740	3	179	PxW254 - 3	1190254	Morocco	UoN
343	343	3	180	PxW254 - 24	1190254	Morocco	UoN

903	903	1	180	PxW254 - 24	1190254	Morocco	UoN
928	928	2	180	PxW254 - 24	1190254	Morocco	UoN
347	347	3	181	PxW254 - 39	1190254	Morocco	UoN
785	785	1	181	PxW254 - 39	1190254	Morocco	UoN
1118	1118	2	181	PxW254 - 39	1190254	Morocco	UoN
569	569	2	182	PxW254 - 40	1190254	Morocco	UoN
717	717	3	182	PxW254 - 40	1190254	Morocco	UoN
1138	1138	1	182	PxW254 - 40	1190254	Morocco	UoN
137	137	3	183	PxW254 - 52	1190254	Morocco	UoN
988	988	2	183	PxW254 - 52	1190254	Morocco	UoN
1128	1128	1	183	PxW254 - 52	1190254	Morocco	UoN
704	704	3	184	PxW254 - 55	1190254	Morocco	UoN
934	934	2	184	PxW254 - 55	1190254	Morocco	UoN
1156	1156	1	184	PxW254 - 55	1190254	Morocco	UoN
355	355	3	185	PxW254 - 59	1190254	Morocco	UoN
419	419	1	185	PxW254 - 59	1190254	Morocco	UoN
1180	1180	2	185	PxW254 - 59	1190254	Morocco	UoN
242	242	3	186	PxW254 - 69	1190254	Morocco	UoN
507	507	2	186	PxW254 - 69	1190254	Morocco	UoN
763	763	1	186	PxW254 - 69	1190254	Morocco	UoN
237	237	3	187	PxW254 - 74	1190254	Morocco	UoN
677	677	1	187	PxW254 - 74	1190254	Morocco	UoN
993	993	2	187	PxW254 - 74	1190254	Morocco	UoN
486	486	3	188	PxW254 - 76	1190254	Morocco	UoN
547	547	1	188	PxW254 - 76	1190254	Morocco	UoN
1000	1000	2	188	PxW254 - 76	1190254	Morocco	UoN
454	454	2	189	PxW254 - 84	1190254	Morocco	UoN
482	482	3	189	PxW254 - 84	1190254	Morocco	UoN
541	541	1	189	PxW254 - 84	1190254	Morocco	UoN
758	758	2	190	PxW254 - 87	1190254	Morocco	UoN
823	823	3	190	PxW254 - 87	1190254	Morocco	UoN
887	887	1	190	PxW254 - 87	1190254	Morocco	UoN
571	571	2	191	PxW264 - 9	1190264	Canary Islands	UoN

761	761	1	191	PxW264 - 9	1190264	Canary Islands	UoN
833	833	3	191	PxW264 - 9	1190264	Canary Islands	UoN
431	431	1	192	PxW264 - 10	1190264	Canary Islands	UoN
496	496	3	192	PxW264 - 10	1190264	Canary Islands	UoN
1173	1173	2	192	PxW264 - 10	1190264	Canary Islands	UoN
430	430	1	193	PxW264 - 12	1190264	Canary Islands	UoN
844	844	3	193	PxW264 - 12	1190264	Canary Islands	UoN
1103	1103	2	193	PxW264 - 12	1190264	Canary Islands	UoN
455	455	2	194	PxW264 - 16	1190264	Canary Islands	UoN
585	585	3	194	PxW264 - 16	1190264	Canary Islands	UoN
1024	1024	1	194	PxW264 - 16	1190264	Canary Islands	UoN
463	463	3	195	PxW264 - 17	1190264	Canary Islands	UoN
518	518	2	195	PxW264 - 17	1190264	Canary Islands	UoN
1007	1007	1	195	PxW264 - 17	1190264	Canary Islands	UoN
305	305	1	196	PxW264 - 31	1190264	Canary Islands	UoN
490	490	3	196	PxW264 - 31	1190264	Canary Islands	UoN
924	924	2	196	PxW264 - 31	1190264	Canary Islands	UoN
477	477	3	197	PxW264 - 33	1190264	Canary Islands	UoN
813	813	2	197	PxW264 - 33	1190264	Canary Islands	UoN
1142	1142	1	197	PxW264 - 33	1190264	Canary Islands	UoN
127	127	3	198	PxW264 - 41	1190264	Canary Islands	UoN
530	530	1	198	PxW264 - 41	1190264	Canary Islands	UoN
984	984	2	198	PxW264 - 41	1190264	Canary Islands	UoN
346	346	3	199	PxW264 - 47	1190264	Canary Islands	UoN
686	686	2	199	PxW264 - 47	1190264	Canary Islands	UoN
1039	1039	1	199	PxW264 - 47	1190264	Canary Islands	UoN
118	118	3	200	PxW264 - 50	1190264	Canary Islands	UoN
656	656	1	200	PxW264 - 50	1190264	Canary Islands	UoN
1047	1047	2	200	PxW264 - 50	1190264	Canary Islands	UoN
109	109	3	201	PxW264 - 51	1190264	Canary Islands	UoN
560	560	1	201	PxW264 - 51	1190264	Canary Islands	UoN
861	861	2	201	PxW264 - 51	1190264	Canary Islands	UoN
588	588	3	202	PxW264 - 52	1190264	Canary Islands	UoN

1112	1112	2	202	PxW264 - 52	1190264	Canary Islands	UoN
1146	1146	1	202	PxW264 - 52	1190264	Canary Islands	UoN
232	232	3	203	PxW264 - 86	1190264	Canary Islands	UoN
1044	1044	2	203	PxW264 - 86	1190264	Canary Islands	UoN
1157	1157	1	203	PxW264 - 86	1190264	Canary Islands	UoN
561	561	2	204	PxW273 - 11	1190273	Spain	UoN
601	601	3	204	PxW273 - 11	1190273	Spain	UoN
673	673	1	204	PxW273 - 11	1190273	Spain	UoN
259	259	3	205	PxW273 - 15	1190273	Spain	UoN
933	933	2	205	PxW273 - 15	1190273	Spain	UoN
1127	1127	1	205	PxW273 - 15	1190273	Spain	UoN
479	479	3	206	PxW273 - 19	1190273	Spain	UoN
875	875	2	206	PxW273 - 19	1190273	Spain	UoN
1148	1148	1	206	PxW273 - 19	1190273	Spain	UoN
391	391	2	207	PxW273 - 21	1190273	Spain	UoN
553	553	1	207	PxW273 - 21	1190273	Spain	UoN
603	603	3	207	PxW273 - 21	1190273	Spain	UoN
445	445	2	208	PxW273 - 26	1190273	Spain	UoN
500	500	3	208	PxW273 - 26	1190273	Spain	UoN
885	885	1	208	PxW273 - 26	1190273	Spain	UoN
465	465	3	209	PxW273 - 35	1190273	Spain	UoN
1031	1031	1	209	PxW273 - 35	1190273	Spain	UoN
1043	1043	2	209	PxW273 - 35	1190273	Spain	UoN
534	534	1	210	PxW273 - 45	1190273	Spain	UoN
716	716	3	210	PxW273 - 45	1190273	Spain	UoN
1107	1107	2	210	PxW273 - 45	1190273	Spain	UoN
226	226	3	211	PxW273 - 52	1190273	Spain	UoN
572	572	2	211	PxW273 - 52	1190273	Spain	UoN
1124	1124	1	211	PxW273 - 52	1190273	Spain	UoN
363	363	3	212	PxW273 - 58	1190273	Spain	UoN
920	920	1	212	PxW273 - 58	1190273	Spain	UoN
1115	1115	2	212	PxW273 - 58	1190273	Spain	UoN
645	645	1	213	PxW273 - 71	1190273	Spain	UoN

839	839	3	213	PxW273 - 71	1190273	Spain	UoN
1164	1164	2	213	PxW273 - 71	1190273	Spain	UoN
358	358	3	214	PxW273 - 79	1190273	Spain	UoN
882	882	1	214	PxW273 - 79	1190273	Spain	UoN
1046	1046	2	214	PxW273 - 79	1190273	Spain	UoN
308	308	1	215	PxW273 - 81	1190273	Spain	UoN
737	737	3	215	PxW273 - 81	1190273	Spain	UoN
991	991	2	215	PxW273 - 81	1190273	Spain	UoN
111	111	3	216	PxW273 - 87	1190273	Spain	UoN
447	447	2	216	PxW273 - 87	1190273	Spain	UoN
786	786	1	216	PxW273 - 87	1190273	Spain	UoN
7	7	3	217	PxW291 - 8	1190291	Cyprus	UoN
458	458	2	217	PxW291 - 8	1190291	Cyprus	UoN
772	772	1	217	PxW291 - 8	1190291	Cyprus	UoN
519	519	2	218	PxW291 - 12	1190291	Cyprus	UoN
834	834	3	218	PxW291 - 12	1190291	Cyprus	UoN
907	907	1	218	PxW291 - 12	1190291	Cyprus	UoN
14	14	3	219	PxW291 - 13	1190291	Cyprus	UoN
871	871	2	219	PxW291 - 13	1190291	Cyprus	UoN
1002	1002	1	219	PxW291 - 13	1190291	Cyprus	UoN
102	102	3	220	PxW291 - 23	1190291	Cyprus	UoN
695	695	2	220	PxW291 - 23	1190291	Cyprus	UoN
1136	1136	1	220	PxW291 - 23	1190291	Cyprus	UoN
357	357	3	221	PxW291 - 25	1190291	Cyprus	UoN
996	996	2	221	PxW291 - 25	1190291	Cyprus	UoN
1158	1158	1	221	PxW291 - 25	1190291	Cyprus	UoN
555	555	1	222	PxW291 - 35	1190291	Cyprus	UoN
584	584	3	222	PxW291 - 35	1190291	Cyprus	UoN
922	922	2	222	PxW291 - 35	1190291	Cyprus	UoN
439	439	1	223	PxW291 - 39	1190291	Cyprus	UoN
629	629	2	223	PxW291 - 39	1190291	Cyprus	UoN
824	824	3	223	PxW291 - 39	1190291	Cyprus	UoN
238	238	3	224	PxW291 - 45	1190291	Cyprus	UoN

448	448	2	224	PxW291 - 45	1190291	Cyprus	UoN
678	678	1	224	PxW291 - 45	1190291	Cyprus	UoN
129	129	3	225	PxW291 - 47	1190291	Cyprus	UoN
752	752	2	225	PxW291 - 47	1190291	Cyprus	UoN
1159	1159	1	225	PxW291 - 47	1190291	Cyprus	UoN
367	367	3	226	PxW291 - 50	1190291	Cyprus	UoN
416	416	1	226	PxW291 - 50	1190291	Cyprus	UoN
759	759	2	226	PxW291 - 50	1190291	Cyprus	UoN
368	368	3	227	PxW291 - 51	1190291	Cyprus	UoN
521	521	1	227	PxW291 - 51	1190291	Cyprus	UoN
995	995	2	227	PxW291 - 51	1190291	Cyprus	UoN
328	328	2	228	PxW291 - 74	1190291	Cyprus	UoN
420	420	1	228	PxW291 - 74	1190291	Cyprus	UoN
701	701	3	228	PxW291 - 74	1190291	Cyprus	UoN
356	356	3	229	PxW291 - 75	1190291	Cyprus	UoN
1004	1004	1	229	PxW291 - 75	1190291	Cyprus	UoN
1058	1058	2	229	PxW291 - 75	1190291	Cyprus	UoN
116	116	3	230	PxW299 - 14	1190299	Turkey	UoN
406	406	1	230	PxW299 - 14	1190299	Turkey	UoN
986	986	2	230	PxW299 - 14	1190299	Turkey	UoN
139	139	3	231	PxW299 - 17	1190299	Turkey	UoN
666	666	1	231	PxW299 - 17	1190299	Turkey	UoN
1106	1106	2	231	PxW299 - 17	1190299	Turkey	UoN
488	488	3	232	PxW299 - 20	1190299	Turkey	UoN
1052	1052	2	232	PxW299 - 20	1190299	Turkey	UoN
1140	1140	1	232	PxW299 - 20	1190299	Turkey	UoN
228	228	3	233	PxW299 - 31	1190299	Turkey	UoN
550	550	1	233	PxW299 - 31	1190299	Turkey	UoN
1056	1056	2	233	PxW299 - 31	1190299	Turkey	UoN
622	622	2	234	PxW299 - 34	1190299	Turkey	UoN
735	735	3	234	PxW299 - 34	1190299	Turkey	UoN
1144	1144	1	234	PxW299 - 34	1190299	Turkey	UoN
240	240	3	235	PxW299 - 40	1190299	Turkey	UoN

660	660	1	235	PxW299 - 40	1190299	Turkey	UoN
985	985	2	235	PxW299 - 40	1190299	Turkey	UoN
558	558	1	236	PxW299 - 47	1190299	Turkey	UoN
730	730	3	236	PxW299 - 47	1190299	Turkey	UoN
1109	1109	2	236	PxW299 - 47	1190299	Turkey	UoN
565	565	2	237	PxW299 - 51	1190299	Turkey	UoN
610	610	3	237	PxW299 - 51	1190299	Turkey	UoN
1008	1008	1	237	PxW299 - 51	1190299	Turkey	UoN
258	258	3	238	PxW299 - 63	1190299	Turkey	UoN
911	911	1	238	PxW299 - 63	1190299	Turkey	UoN
994	994	2	238	PxW299 - 63	1190299	Turkey	UoN
395	395	2	239	PxW299 - 68	1190299	Turkey	UoN
676	676	1	239	PxW299 - 68	1190299	Turkey	UoN
846	846	3	239	PxW299 - 68	1190299	Turkey	UoN
322	322	2	240	PxW299 - 69	1190299	Turkey	UoN
489	489	3	240	PxW299 - 69	1190299	Turkey	UoN
1022	1022	1	240	PxW299 - 69	1190299	Turkey	UoN
11	11	3	241	PxW299 - 78	1190299	Turkey	UoN
393	393	2	241	PxW299 - 78	1190299	Turkey	UoN
767	767	1	241	PxW299 - 78	1190299	Turkey	UoN
664	664	1	242	PxW299 - 87	1190299	Turkey	UoN
715	715	3	242	PxW299 - 87	1190299	Turkey	UoN
1051	1051	2	242	PxW299 - 87	1190299	Turkey	UoN
374	374	3	243	PxW349 - 7	1190349	Bulgaria	UoN
1021	1021	1	243	PxW349 - 7	1190349	Bulgaria	UoN
1113	1113	2	243	PxW349 - 7	1190349	Bulgaria	UoN
556	556	1	244	PxW349 - 10	1190349	Bulgaria	UoN
734	734	3	244	PxW349 - 10	1190349	Bulgaria	UoN
1172	1172	2	244	PxW349 - 10	1190349	Bulgaria	UoN
114	114	3	245	PxW349 - 16	1190349	Bulgaria	UoN
511	511	2	245	PxW349 - 16	1190349	Bulgaria	UoN
790	790	1	245	PxW349 - 16	1190349	Bulgaria	UoN
499	499	3	246	PxW349 - 19	1190349	Bulgaria	UoN

1119	1119	2	246	PxW349 - 19	1190349	Bulgaria	UoN
1131	1131	1	246	PxW349 - 19	1190349	Bulgaria	UoN
595	595	3	247	PxW349 - 22	1190349	Bulgaria	UoN
1153	1153	1	247	PxW349 - 22	1190349	Bulgaria	UoN
1170	1170	2	247	PxW349 - 22	1190349	Bulgaria	UoN
316	316	1	248	PxW349 - 28	1190349	Bulgaria	UoN
492	492	3	248	PxW349 - 28	1190349	Bulgaria	UoN
878	878	2	248	PxW349 - 28	1190349	Bulgaria	UoN
339	339	2	249	PxW349 - 31	1190349	Bulgaria	UoN
661	661	1	249	PxW349 - 31	1190349	Bulgaria	UoN
857	857	3	249	PxW349 - 31	1190349	Bulgaria	UoN
106	106	3	250	PxW349 - 42	1190349	Bulgaria	UoN
625	625	2	250	PxW349 - 42	1190349	Bulgaria	UoN
674	674	1	250	PxW349 - 42	1190349	Bulgaria	UoN
224	224	3	251	PxW349 - 46	1190349	Bulgaria	UoN
742	742	2	251	PxW349 - 46	1190349	Bulgaria	UoN
782	782	1	251	PxW349 - 46	1190349	Bulgaria	UoN
327	327	2	252	PxW349 - 58	1190349	Bulgaria	UoN
848	848	3	252	PxW349 - 58	1190349	Bulgaria	UoN
895	895	1	252	PxW349 - 58	1190349	Bulgaria	UoN
378	378	3	253	PxW349 - 65	1190349	Bulgaria	UoN
921	921	2	253	PxW349 - 65	1190349	Bulgaria	UoN
1012	1012	1	253	PxW349 - 65	1190349	Bulgaria	UoN
441	441	2	254	PxW349 - 66	1190349	Bulgaria	UoN
529	529	1	254	PxW349 - 66	1190349	Bulgaria	UoN
826	826	3	254	PxW349 - 66	1190349	Bulgaria	UoN
608	608	3	255	PxW349 - 72	1190349	Bulgaria	UoN
650	650	1	255	PxW349 - 72	1190349	Bulgaria	UoN
989	989	2	255	PxW349 - 72	1190349	Bulgaria	UoN
736	736	3	256	PxW396 - 5	1190396	Portugal	UoN
777	777	1	256	PxW396 - 5	1190396	Portugal	UoN
1116	1116	2	256	PxW396 - 5	1190396	Portugal	UoN
619	619	3	257	PxW396 - 12	1190396	Portugal	UoN

693	693	2	257	PxW396 - 12	1190396	Portugal	UoN
1151	1151	1	257	PxW396 - 12	1190396	Portugal	UoN
244	244	3	258	PxW396 - 19	1190396	Portugal	UoN
318	318	1	258	PxW396 - 19	1190396	Portugal	UoN
741	741	2	258	PxW396 - 19	1190396	Portugal	UoN
17	17	3	259	PxW396 - 30	1190396	Portugal	UoN
317	317	1	259	PxW396 - 30	1190396	Portugal	UoN
927	927	2	259	PxW396 - 30	1190396	Portugal	UoN
473	473	3	260	PxW396 - 37	1190396	Portugal	UoN
981	981	2	260	PxW396 - 37	1190396	Portugal	UoN
1035	1035	1	260	PxW396 - 37	1190396	Portugal	UoN
246	246	3	261	PxW396 - 46	1190396	Portugal	UoN
808	808	2	261	PxW396 - 46	1190396	Portugal	UoN
889	889	1	261	PxW396 - 46	1190396	Portugal	UoN
467	467	3	262	PxW396 - 48	1190396	Portugal	UoN
688	688	2	262	PxW396 - 48	1190396	Portugal	UoN
914	914	1	262	PxW396 - 48	1190396	Portugal	UoN
590	590	3	263	PxW396 - 49	1190396	Portugal	UoN
806	806	2	263	PxW396 - 49	1190396	Portugal	UoN
1037	1037	1	263	PxW396 - 49	1190396	Portugal	UoN
323	323	2	264	PxW396 - 51	1190396	Portugal	UoN
370	370	3	264	PxW396 - 51	1190396	Portugal	UoN
422	422	1	264	PxW396 - 51	1190396	Portugal	UoN
229	229	3	265	PxW396 - 56	1190396	Portugal	UoN
789	789	1	265	PxW396 - 56	1190396	Portugal	UoN
812	812	2	265	PxW396 - 56	1190396	Portugal	UoN
636	636	2	266	PxW396 - 60	1190396	Portugal	UoN
705	705	3	266	PxW396 - 60	1190396	Portugal	UoN
1026	1026	1	266	PxW396 - 60	1190396	Portugal	UoN
612	612	3	267	PxW396 - 62	1190396	Portugal	UoN
865	865	2	267	PxW396 - 62	1190396	Portugal	UoN
1129	1129	1	267	PxW396 - 62	1190396	Portugal	UoN
637	637	2	268	PxW396 - 73	1190396	Portugal	UoN

718	718	3	268	PxW396 - 73	1190396	Portugal	UoN
771	771	1	268	PxW396 - 73	1190396	Portugal	UoN
803	803	2	269	PxW397 - 20	1190397	Portugal	UoN
849	849	3	269	PxW397 - 20	1190397	Portugal	UoN
918	918	1	269	PxW397 - 20	1190397	Portugal	UoN
119	119	3	270	PxW397 - 26	1190397	Portugal	UoN
936	936	2	270	PxW397 - 26	1190397	Portugal	UoN
1038	1038	1	270	PxW397 - 26	1190397	Portugal	UoN
6	6	3	271	PxW397 - 33	1190397	Portugal	UoN
382	382	2	271	PxW397 - 33	1190397	Portugal	UoN
905	905	1	271	PxW397 - 33	1190397	Portugal	UoN
133	133	3	272	PxW397 - 48	1190397	Portugal	UoN
820	820	2	272	PxW397 - 48	1190397	Portugal	UoN
1018	1018	1	272	PxW397 - 48	1190397	Portugal	UoN
630	630	2	273	PxW397 - 50	1190397	Portugal	UoN
794	794	1	273	PxW397 - 50	1190397	Portugal	UoN
847	847	3	273	PxW397 - 50	1190397	Portugal	UoN
136	136	3	274	PxW397 - 51	1190397	Portugal	UoN
787	787	1	274	PxW397 - 51	1190397	Portugal	UoN
864	864	2	274	PxW397 - 51	1190397	Portugal	UoN
331	331	2	275	PxW397 - 61	1190397	Portugal	UoN
724	724	3	275	PxW397 - 61	1190397	Portugal	UoN
784	784	1	275	PxW397 - 61	1190397	Portugal	UoN
134	134	3	276	PxW397 - 76	1190397	Portugal	UoN
570	570	2	276	PxW397 - 76	1190397	Portugal	UoN
881	881	1	276	PxW397 - 76	1190397	Portugal	UoN
506	506	2	277	PxW397 - 80	1190397	Portugal	UoN
591	591	3	277	PxW397 - 80	1190397	Portugal	UoN
909	909	1	277	PxW397 - 80	1190397	Portugal	UoN
310	310	1	278	PxW397 - 82	1190397	Portugal	UoN
369	369	3	278	PxW397 - 82	1190397	Portugal	UoN
1048	1048	2	278	PxW397 - 82	1190397	Portugal	UoN
452	452	2	279	PxW397 - 83	1190397	Portugal	UoN

653	653	1	279	PxW397 - 83	1190397	Portugal	UoN
702	702	3	279	PxW397 - 83	1190397	Portugal	UoN
415	415	1	280	PxW397 - 88	1190397	Portugal	UoN
450	450	2	280	PxW397 - 88	1190397	Portugal	UoN
613	613	3	280	PxW397 - 88	1190397	Portugal	UoN
239	239	3	281	PxW397 - 89	1190397	Portugal	UoN
564	564	2	281	PxW397 - 89	1190397	Portugal	UoN
908	908	1	281	PxW397 - 89	1190397	Portugal	UoN
319	319	1	282	PxW398 - 18	1190398	Palestine	UoN
388	388	2	282	PxW398 - 18	1190398	Palestine	UoN
594	594	3	282	PxW398 - 18	1190398	Palestine	UoN
235	235	3	283	PxW398 - 21	1190398	Palestine	UoN
883	883	1	283	PxW398 - 21	1190398	Palestine	UoN
930	930	2	283	PxW398 - 21	1190398	Palestine	UoN
9	9	3	284	PxW398 - 41	1190398	Palestine	UoN
414	414	1	284	PxW398 - 41	1190398	Palestine	UoN
873	873	2	284	PxW398 - 41	1190398	Palestine	UoN
4	4	3	285	PxW398 - 42	1190398	Palestine	UoN
433	433	1	285	PxW398 - 42	1190398	Palestine	UoN
804	804	2	285	PxW398 - 42	1190398	Palestine	UoN
687	687	2	286	PxW398 - 44	1190398	Palestine	UoN
731	731	3	286	PxW398 - 44	1190398	Palestine	UoN
1123	1123	1	286	PxW398 - 44	1190398	Palestine	UoN
380	380	3	287	PxW398 - 49	1190398	Palestine	UoN
802	802	2	287	PxW398 - 49	1190398	Palestine	UoN
1139	1139	1	287	PxW398 - 49	1190398	Palestine	UoN
138	138	3	288	PxW398 - 55	1190398	Palestine	UoN
438	438	1	288	PxW398 - 55	1190398	Palestine	UoN
1057	1057	2	288	PxW398 - 55	1190398	Palestine	UoN
409	409	1	289	PxW398 - 56	1190398	Palestine	UoN
733	733	3	289	PxW398 - 56	1190398	Palestine	UoN
819	819	2	289	PxW398 - 56	1190398	Palestine	UoN
599	599	3	290	PxW398 - 60	1190398	Palestine	UoN

679	679	1	290	PxW398 - 60	1190398	Palestine	UoN
1169	1169	2	290	PxW398 - 60	1190398	Palestine	UoN
110	110	3	291	PxW398 - 63	1190398	Palestine	UoN
888	888	1	291	PxW398 - 63	1190398	Palestine	UoN
1111	1111	2	291	PxW398 - 63	1190398	Palestine	UoN
505	505	2	292	PxW398 - 74	1190398	Palestine	UoN
611	611	3	292	PxW398 - 74	1190398	Palestine	UoN
1030	1030	1	292	PxW398 - 74	1190398	Palestine	UoN
354	354	3	293	PxW398 - 81	1190398	Palestine	UoN
563	563	2	293	PxW398 - 81	1190398	Palestine	UoN
788	788	1	293	PxW398 - 81	1190398	Palestine	UoN
222	222	3	294	PxW398 - 85	1190398	Palestine	UoN
333	333	2	294	PxW398 - 85	1190398	Palestine	UoN
1009	1009	1	294	PxW398 - 85	1190398	Palestine	UoN
682	682	2	295	PxW420 - 1	1190420	India	UoN
725	725	3	295	PxW420 - 1	1190420	India	UoN
792	792	1	295	PxW420 - 1	1190420	India	UoN
404	404	1	296	PxW420 - 3	1190420	India	UoN
831	831	3	296	PxW420 - 3	1190420	India	UoN
1045	1045	2	296	PxW420 - 3	1190420	India	UoN
399	399	2	297	PxW420 - 8	1190420	India	UoN
494	494	3	297	PxW420 - 8	1190420	India	UoN
916	916	1	297	PxW420 - 8	1190420	India	UoN
424	424	1	298	PxW420 - 10	1190420	India	UoN
746	746	2	298	PxW420 - 10	1190420	India	UoN
850	850	3	298	PxW420 - 10	1190420	India	UoN
260	260	3	299	PxW420 - 11	1190420	India	UoN
302	302	1	299	PxW420 - 11	1190420	India	UoN
1101	1101	2	299	PxW420 - 11	1190420	India	UoN
20	20	3	300	PxW420 - 21	1190420	India	UoN
694	694	2	300	PxW420 - 21	1190420	India	UoN
891	891	1	300	PxW420 - 21	1190420	India	UoN
253	253	3	301	PxW420 - 22	1190420	India	UoN

385	385	2	301	PxW420 - 22	1190420	India	UoN
1023	1023	1	301	PxW420 - 22	1190420	India	UoN
841	841	3	302	PxW420 - 25	1190420	India	UoN
868	868	2	302	PxW420 - 25	1190420	India	UoN
1150	1150	1	302	PxW420 - 25	1190420	India	UoN
15	15	3	303	PxW420 - 31	1190420	India	UoN
527	527	1	303	PxW420 - 31	1190420	India	UoN
1178	1178	2	303	PxW420 - 31	1190420	India	UoN
345	345	3	304	PxW420 - 32	1190420	India	UoN
762	762	1	304	PxW420 - 32	1190420	India	UoN
1102	1102	2	304	PxW420 - 32	1190420	India	UoN
390	390	2	305	PxW420 - 37	1190420	India	UoN
830	830	3	305	PxW420 - 37	1190420	India	UoN
893	893	1	305	PxW420 - 37	1190420	India	UoN
365	365	3	306	PxW420 - 67	1190420	India	UoN
384	384	2	306	PxW420 - 67	1190420	India	UoN
672	672	1	306	PxW420 - 67	1190420	India	UoN
697	697	2	307	PxW420 - 94	1190420	India	UoN
712	712	3	307	PxW420 - 94	1190420	India	UoN
1006	1006	1	307	PxW420 - 94	1190420	India	UoN
301	301	1	308	PxW546 - 3	1190546	Spain	UoN
344	344	3	308	PxW546 - 3	1190546	Spain	UoN
700	700	2	308	PxW546 - 3	1190546	Spain	UoN
436	436	1	309	PxW546 - 8	1190546	Spain	UoN
475	475	3	309	PxW546 - 8	1190546	Spain	UoN
811	811	2	309	PxW546 - 8	1190546	Spain	UoN
359	359	3	310	PxW546 - 12	1190546	Spain	UoN
396	396	2	310	PxW546 - 12	1190546	Spain	UoN
898	898	1	310	PxW546 - 12	1190546	Spain	UoN
329	329	2	311	PxW546 - 15	1190546	Spain	UoN
497	497	3	311	PxW546 - 15	1190546	Spain	UoN
1141	1141	1	311	PxW546 - 15	1190546	Spain	UoN
566	566	2	312	PxW546 - 16	1190546	Spain	UoN

593	593	3	312	PxW546 - 16	1190546	Spain	UoN
884	884	1	312	PxW546 - 16	1190546	Spain	UoN
313	313	1	313	PxW546 - 20	1190546	Spain	UoN
720	720	3	313	PxW546 - 20	1190546	Spain	UoN
743	743	2	313	PxW546 - 20	1190546	Spain	UoN
320	320	1	314	PxW546 - 24	1190546	Spain	UoN
326	326	2	314	PxW546 - 24	1190546	Spain	UoN
618	618	3	314	PxW546 - 24	1190546	Spain	UoN
524	524	1	315	PxW546 - 25	1190546	Spain	UoN
852	852	3	315	PxW546 - 25	1190546	Spain	UoN
931	931	2	315	PxW546 - 25	1190546	Spain	UoN
383	383	2	316	PxW546 - 27	1190546	Spain	UoN
428	428	1	316	PxW546 - 27	1190546	Spain	UoN
726	726	3	316	PxW546 - 27	1190546	Spain	UoN
495	495	3	317	PxW546 - 29	1190546	Spain	UoN
575	575	2	317	PxW546 - 29	1190546	Spain	UoN
1027	1027	1	317	PxW546 - 29	1190546	Spain	UoN
248	248	3	318	PxW546 - 32	1190546	Spain	UoN
321	321	2	318	PxW546 - 32	1190546	Spain	UoN
800	800	1	318	PxW546 - 32	1190546	Spain	UoN
503	503	2	319	PxW546 - 38	1190546	Spain	UoN
522	522	1	319	PxW546 - 38	1190546	Spain	UoN
829	829	3	319	PxW546 - 38	1190546	Spain	UoN
616	616	3	320	PxW546 - 47	1190546	Spain	UoN
675	675	1	320	PxW546 - 47	1190546	Spain	UoN
1104	1104	2	320	PxW546 - 47	1190546	Spain	UoN
128	128	3	321	PxW566 - 7	1190566	Greece	UoN
624	624	2	321	PxW566 - 7	1190566	Greece	UoN
1137	1137	1	321	PxW566 - 7	1190566	Greece	UoN
508	508	2	322	PxW566 - 12	1190566	Greece	UoN
615	615	3	322	PxW566 - 12	1190566	Greece	UoN
1133	1133	1	322	PxW566 - 12	1190566	Greece	UoN
443	443	2	323	PxW566 - 14	1190566	Greece	UoN

602	602	3	323	PxW566 - 14	1190566	Greece	UoN
1149	1149	1	323	PxW566 - 14	1190566	Greece	UoN
598	598	3	324	PxW566 - 17	1190566	Greece	UoN
628	628	2	324	PxW566 - 17	1190566	Greece	UoN
1135	1135	1	324	PxW566 - 17	1190566	Greece	UoN
412	412	1	325	PxW566 - 20	1190566	Greece	UoN
681	681	2	325	PxW566 - 20	1190566	Greece	UoN
827	827	3	325	PxW566 - 20	1190566	Greece	UoN
254	254	3	326	PxW566 - 21	1190566	Greece	UoN
638	638	2	326	PxW566 - 21	1190566	Greece	UoN
1147	1147	1	326	PxW566 - 21	1190566	Greece	UoN
103	103	3	327	PxW566 - 23	1190566	Greece	UoN
577	577	2	327	PxW566 - 23	1190566	Greece	UoN
1034	1034	1	327	PxW566 - 23	1190566	Greece	UoN
340	340	2	328	PxW566 - 24	1190566	Greece	UoN
649	649	1	328	PxW566 - 24	1190566	Greece	UoN
828	828	3	328	PxW566 - 24	1190566	Greece	UoN
483	483	3	329	PxW566 - 35	1190566	Greece	UoN
755	755	2	329	PxW566 - 35	1190566	Greece	UoN
1014	1014	1	329	PxW566 - 35	1190566	Greece	UoN
350	350	3	330	PxW566 - 50	1190566	Greece	UoN
745	745	2	330	PxW566 - 50	1190566	Greece	UoN
1033	1033	1	330	PxW566 - 50	1190566	Greece	UoN
373	373	3	331	PxW566 - 52	1190566	Greece	UoN
631	631	2	331	PxW566 - 52	1190566	Greece	UoN
896	896	1	331	PxW566 - 52	1190566	Greece	UoN
337	337	2	332	PxW566 - 72	1190566	Greece	UoN
476	476	3	332	PxW566 - 72	1190566	Greece	UoN
890	890	1	332	PxW566 - 72	1190566	Greece	UoN
517	517	2	333	PxW566 - 92	1190566	Greece	UoN
596	596	3	333	PxW566 - 92	1190566	Greece	UoN
641	641	1	333	PxW566 - 92	1190566	Greece	UoN
121	121	3	334	PxW685 - 1	1190685	Spain	UoN

513	513	2	334	PxW685 - 1	1190685	Spain	UoN
892	892	1	334	PxW685 - 1	1190685	Spain	UoN
617	617	3	335	PxW685 - 6	1190685	Spain	UoN
640	640	2	335	PxW685 - 6	1190685	Spain	UoN
910	910	1	335	PxW685 - 6	1190685	Spain	UoN
418	418	1	336	PxW685 - 7	1190685	Spain	UoN
471	471	3	336	PxW685 - 7	1190685	Spain	UoN
1117	1117	2	336	PxW685 - 7	1190685	Spain	UoN
330	330	2	337	PxW685 - 9	1190685	Spain	UoN
540	540	1	337	PxW685 - 9	1190685	Spain	UoN
586	586	3	337	PxW685 - 9	1190685	Spain	UoN
815	815	2	338	PxW685 - 12	1190685	Spain	UoN
825	825	3	338	PxW685 - 12	1190685	Spain	UoN
1122	1122	1	338	PxW685 - 12	1190685	Spain	UoN
568	568	2	339	PxW685 - 16	1190685	Spain	UoN
710	710	3	339	PxW685 - 16	1190685	Spain	UoN
1155	1155	1	339	PxW685 - 16	1190685	Spain	UoN
514	514	2	340	PxW685 - 22	1190685	Spain	UoN
671	671	1	340	PxW685 - 22	1190685	Spain	UoN
723	723	3	340	PxW685 - 22	1190685	Spain	UoN
126	126	3	341	PxW685 - 36	1190685	Spain	UoN
654	654	1	341	PxW685 - 36	1190685	Spain	UoN
932	932	2	341	PxW685 - 36	1190685	Spain	UoN
573	573	2	342	PxW685 - 41	1190685	Spain	UoN
855	855	3	342	PxW685 - 41	1190685	Spain	UoN
1015	1015	1	342	PxW685 - 41	1190685	Spain	UoN
3	3	3	343	PxW685 - 44	1190685	Spain	UoN
528	528	1	343	PxW685 - 44	1190685	Spain	UoN
816	816	2	343	PxW685 - 44	1190685	Spain	UoN
230	230	3	344	PxW685 - 55	1190685	Spain	UoN
309	309	1	344	PxW685 - 55	1190685	Spain	UoN
814	814	2	344	PxW685 - 55	1190685	Spain	UoN
360	360	3	345	PxW685 - 80	1190685	Spain	UoN

862	862	2	345	PxW685 - 80	1190685	Spain	UoN
902	902	1	345	PxW685 - 80	1190685	Spain	UoN
342	342	3	346	PxW685 - 88	1190685	Spain	UoN
434	434	1	346	PxW685 - 88	1190685	Spain	UoN
937	937	2	346	PxW685 - 88	1190685	Spain	UoN
130	130	3	347	PxW811 - 10	1190811	Tunisia	UoN
751	751	2	347	PxW811 - 10	1190811	Tunisia	UoN
779	779	1	347	PxW811 - 10	1190811	Tunisia	UoN
487	487	3	348	PxW811 - 26	1190811	Tunisia	UoN
652	652	1	348	PxW811 - 26	1190811	Tunisia	UoN
1114	1114	2	348	PxW811 - 26	1190811	Tunisia	UoN
257	257	3	349	PxW811 - 28	1190811	Tunisia	UoN
413	413	1	349	PxW811 - 28	1190811	Tunisia	UoN
879	879	2	349	PxW811 - 28	1190811	Tunisia	UoN
140	140	3	350	PxW811 - 30	1190811	Tunisia	UoN
557	557	1	350	PxW811 - 30	1190811	Tunisia	UoN
940	940	2	350	PxW811 - 30	1190811	Tunisia	UoN
233	233	3	351	PxW811 - 42	1190811	Tunisia	UoN
769	769	1	351	PxW811 - 42	1190811	Tunisia	UoN
1162	1162	2	351	PxW811 - 42	1190811	Tunisia	UoN
551	551	1	352	PxW811 - 50	1190811	Tunisia	UoN
634	634	2	352	PxW811 - 50	1190811	Tunisia	UoN
821	821	3	352	PxW811 - 50	1190811	Tunisia	UoN
303	303	1	353	PxW811 - 56	1190811	Tunisia	UoN
509	509	2	353	PxW811 - 56	1190811	Tunisia	UoN
719	719	3	353	PxW811 - 56	1190811	Tunisia	UoN
397	397	2	354	PxW811 - 59	1190811	Tunisia	UoN
491	491	3	354	PxW811 - 59	1190811	Tunisia	UoN
1005	1005	1	354	PxW811 - 59	1190811	Tunisia	UoN
311	311	1	355	PxW811 - 60	1190811	Tunisia	UoN
805	805	2	355	PxW811 - 60	1190811	Tunisia	UoN
854	854	3	355	PxW811 - 60	1190811	Tunisia	UoN
749	749	2	356	PxW811 - 83	1190811	Tunisia	UoN

840	840	3	356	PxW811 - 83	1190811	Tunisia	UoN
1145	1145	1	356	PxW811 - 83	1190811	Tunisia	UoN
2	2	3	357	PxW811 - 84	1190811	Tunisia	UoN
632	632	2	357	PxW811 - 84	1190811	Tunisia	UoN
1028	1028	1	357	PxW811 - 84	1190811	Tunisia	UoN
335	335	2	358	PxW811 - 90	1190811	Tunisia	UoN
607	607	3	358	PxW811 - 90	1190811	Tunisia	UoN
778	778	1	358	PxW811 - 90	1190811	Tunisia	UoN
437	437	1	359	PxW811 - 96	1190811	Tunisia	UoN
605	605	3	359	PxW811 - 96	1190811	Tunisia	UoN
987	987	2	359	PxW811 - 96	1190811	Tunisia	UoN
				PxW216 - 3			Rres
				PxW216 - 30			Rres
				PxW216 - 4			Rres
				PxW216 - 45			Rres
				PxW216 - 48			Rres
				PxW216 - 74			Rres
				PxW216 - 76			Rres
				PxW216 - 81			Rres
				PxW216 - 85			Rres
				PxW216 - 88			Rres
				PxW216 - 89			Rres
				PxW216 - 92			Rres
				PxW216 - 94			Rres
				PxW223 - 1			Rres
				PxW223 - 2			Rres
				PxW223 - 25			Rres
				PxW223 - 3			Rres
				PxW223 - 80			Rres
				PxW223 - 83			Rres
				PxW223 - 85			Rres
				PxW223 - 86			Rres
				PxW223 - 89			Rres

PxW223 - 90	Rres
PxW223 - 91	Rres
PxW223 - 92	Rres
PxW223 - 94	Rres
PxW254 - 2	Rres
PxW254 - 24	Rres
PxW254 - 3	Rres
PxW254 - 39	Rres
PxW254 - 40	Rres
PxW254 - 52	Rres
PxW254 - 55	Rres
PxW254 - 59	Rres
PxW254 - 69	Rres
PxW254 - 74	Rres
PxW254 - 76	Rres
PxW254 - 84	Rres
PxW254 - 87	Rres
PxW264 - 10	Rres
PxW264 - 12	Rres
PxW264 - 16	Rres
PxW264 - 17	Rres
PxW264 - 31	Rres
PxW264 - 33	Rres
PxW264 - 41	Rres
PxW264 - 47	Rres
PxW264 - 50	Rres
PxW264 - 51	Rres
PxW264 - 52	Rres
PxW264 - 86	Rres
PxW264 - 9	Rres
PxW273 - 11	Rres
PxW273 - 15	Rres
PxW273 - 19	Rres

PxW273 - 21	Rres
PxW273 - 26	Rres
PxW273 - 35	Rres
PxW273 - 45	Rres
PxW273 - 52	Rres
PxW273 - 58	Rres
PxW273 - 71	Rres
PxW273 - 79	Rres
PxW273 - 81	Rres
PxW273 - 87	Rres
PxW291 - 12	Rres
PxW291 - 13	Rres
PxW291 - 23	Rres
PxW291 - 25	Rres
PxW291 - 35	Rres
PxW291 - 39	Rres
PxW291 - 45	Rres
PxW291 - 47	Rres
PxW291 - 50	Rres
PxW291 - 51	Rres
PxW291 - 74	Rres
PxW291 - 75	Rres
PxW291 - 8	Rres
PxW299 - 14	Rres
PxW299 - 17	Rres
PxW299 - 20	Rres
PxW299 - 31	Rres
PxW299 - 34	Rres
PxW299 - 40	Rres
PxW299 - 47	Rres
PxW299 - 51	Rres
PxW299 - 63	Rres
PxW299 - 68	Rres

PxW299 - 69	Rres
PxW299 - 78	Rres
PxW299 - 87	Rres
PxW32 - 27	Rres
PxW32 - 29	Rres
PxW32 - 50	Rres
PxW32 - 56	Rres
PxW32 - 57	Rres
PxW32 - 60	Rres
PxW32 - 61	Rres
PxW32 - 62	Rres
PxW32 - 66	Rres
PxW32 - 76	Rres
PxW32 - 8	Rres
PxW32 - 81	Rres
PxW349 - 10	Rres
PxW349 - 16	Rres
PxW349 - 19	Rres
PxW349 - 22	Rres
PxW349 - 28	Rres
PxW349 - 31	Rres
PxW349 - 42	Rres
PxW349 - 46	Rres
PxW349 - 58	Rres
PxW349 - 65	Rres
PxW349 - 66	Rres
PxW349 - 7	Rres
PxW349 - 72	Rres
PxW396 - 12	Rres
PxW396 - 19	Rres
PxW396 - 30	Rres
PxW396 - 37	Rres
PxW396 - 46	Rres

PxW396 - 48	Rres
PxW396 - 49	Rres
PxW396 - 5	Rres
PxW396 - 51	Rres
PxW396 - 56	Rres
PxW396 - 60	Rres
PxW396 - 62	Rres
PxW396 - 73	Rres
PxW397 - 20	Rres
PxW397 - 26	Rres
PxW397 - 33	Rres
PxW397 - 48	Rres
PxW397 - 50	Rres
PxW397 - 51	Rres
PxW397 - 61	Rres
PxW397 - 76	Rres
PxW397 - 80	Rres
PxW397 - 82	Rres
PxW397 - 83	Rres
PxW397 - 88	Rres
PxW397 - 89	Rres
PxW398 - 18	Rres
PxW398 - 21	Rres
PxW398 - 41	Rres
PxW398 - 42	Rres
PxW398 - 44	Rres
PxW398 - 49	Rres
PxW398 - 55	Rres
PxW398 - 56	Rres
PxW398 - 60	Rres
PxW398 - 63	Rres
PxW398 - 74	Rres
PxW398 - 81	Rres

PxW398 - 85	Rres
PxW42 - 14	Rres
PxW42 - 17	Rres
PxW42 - 18	Rres
PxW42 - 22	Rres
PxW42 - 33	Rres
PxW42 - 34	Rres
PxW42 - 45	Rres
PxW42 - 60	Rres
PxW42 - 68	Rres
PxW42 - 75	Rres
PxW42 - 90	Rres
PxW42 - 91	Rres
PxW42 - 93	Rres
PxW420 - 1	Rres
PxW420 - 10	Rres
PxW420 - 11	Rres
PxW420 - 21	Rres
PxW420 - 22	Rres
PxW420 - 25	Rres
PxW420 - 3	Rres
PxW420 - 31	Rres
PxW420 - 32	Rres
PxW420 - 37	Rres
PxW420 - 67	Rres
PxW420 - 8	Rres
PxW420 - 94	Rres
PxW546 - 12	Rres
PxW546 - 15	Rres
PxW546 - 16	Rres
PxW546 - 20	Rres
PxW546 - 24	Rres
PxW546 - 25	Rres

PxW546 - 27	Rres
PxW546 - 29	Rres
PxW546 - 3	Rres
PxW546 - 32	Rres
PxW546 - 38	Rres
PxW546 - 47	Rres
PxW546 - 8	Rres
PxW566 - 12	Rres
PxW566 - 14	Rres
PxW566 - 17	Rres
PxW566 - 20	Rres
PxW566 - 21	Rres
PxW566 - 23	Rres
PxW566 - 24	Rres
PxW566 - 35	Rres
PxW566 - 50	Rres
PxW566 - 52	Rres
PxW566 - 7	Rres
PxW566 - 72	Rres
PxW566 - 92	Rres
PxW685 - 1	Rres
PxW685 - 12	Rres
PxW685 - 16	Rres
PxW685 - 22	Rres
PxW685 - 36	Rres
PxW685 - 41	Rres
PxW685 - 44	Rres
PxW685 - 55	Rres
PxW685 - 6	Rres
PxW685 - 7	Rres
PxW685 - 80	Rres
PxW685 - 88	Rres
PxW685 - 9	Rres

PxW7 - 15	Rres
PxW7 - 18	Rres
PxW7 - 2	Rres
PxW7 - 29	Rres
PxW7 - 3	Rres
PxW7 - 32	Rres
PxW7 - 47	Rres
PxW7 - 60	Rres
PxW7 - 71	Rres
PxW7 - 76	Rres
PxW7 - 77	Rres
PxW7 - 87	Rres
PxW811 - 10	Rres
PxW811 - 26	Rres
PxW811 - 28	Rres
PxW811 - 30	Rres
PxW811 - 42	Rres
PxW811 - 50	Rres
PxW811 - 56	Rres
PxW811 - 59	Rres
PxW811 - 60	Rres
PxW811 - 83	Rres
PxW811 - 84	Rres
PxW811 - 90	Rres
PxW811 - 96	Rres

nd Rothamsted sites in 2015-16.

Year!	Fe_mg_kg⁻¹	Zn_mg_kg⁻¹	Straw_N%	Grain_N%	Gprot%	TGW_g	GYD_t ha⁻¹_85% DM
2016	40.45277297	33.50241283	1.148612866	2.71724434	15.48829274	29.3	4.747717647
2016	40.72299305	40.35900601	1.145420048	2.278334557	12.98650697	30.8	5.325105882
2016	40.80268207	38.30220382	1.645166865	3.331963602	18.99219253	28.95	3.407352941
2016	30.05306265	22.42331648	1.451820523	3.055289567	17.41515053	37.55	5.904811765
2016	37.20404747	23.11605976	1.868054306	2.866368805	16.33830219	31.8	4.498023529
2016	36.67044798	32.12836234	1.279685411	3.504888427	19.97786403	38.85	4.172235294
2016	37.20480506	27.66443987	1.3719857	2.035860105	11.6044026	35.8	5.719929412
2016	43.68787172	43.08664601	1.062203531			34.65	5.553035294
2016	43.91571391	29.36570169	1.869649447	3.016741355	17.19542572	36.95	
2016	34.94585503	28.36714685	1.106561621	3.093753596	17.6343955	38.9	5.686588235
2016	44.17630308	31.62923201	1.895848694	2.351745462	13.40494914	35.65	4.668188235
2016	31.32804775	28.99096868	1.709414776	2.71660503	15.48464867	37.35	6.032282353
2016	36.42059395	27.80467796	1.124251913	2.450193203	13.96610126	39.9	5.114352941
2016	28.5158976	27.40232372	1.0239401	2.621137987	14.94048653	39.3	4.951623529
2016	39.3448135	28.55157297	1.607223581	1.987085954	11.32638994	35.45	5.340447059
2016	38.99240706	43.48562061	1.057055591	2.703812837	15.41173317	37.5	3.983823529
2016	47.28687155	46.27500198	1.466865906	3.243124395	18.48580905	34.4	3.778823529
2016	35.94444124	50.15397527	1.863951323	3.200181345	18.24103367	36.8	3.693717647
2016	37.62033275	34.5023377	1.231864964	3.073328139	17.51797039	33.7	5.279835294
2016	42.88009998	32.9288662	1.447197798	3.125406548	17.81481732	33.3	4.410352941
2016	32.80200931	42.71117004	1.669622139	2.72724481	15.54529542		4.759411765
2016	28.36273929	25.85153871	1.643933154	2.475347685	14.1094818	35.55	4.762352941
2016	33.99033439	27.33382143	1.440767108	2.427490207	13.83669418	34.34	5.974023529
2016	29.71155697	23.31260522	1.878552865	2.853318254	16.26391405	33.15	3.306
2016	30.87851787	31.97126135	1.498807976	3.028331327	17.26148856	34.85	6.035294118
2016	38.46817122	33.27332534	1.021009999	0.697620303	3.976435725	30.5	3.944470588
2016	37.72201022	36.74990739	1.587406927	3.017406524	17.19921719	34.9	5.691505882
2016	54.04465631	38.60471014	1.676037928	3.137432868	17.88336735	36.86	4.926
2016	48.36297453	43.98433619	2.55183072	2.921831466	16.65443936	37.45	3.582117647
2016	54.19679135	57.34070016	1.552416743	2.86837023	16.34971031		3.644235294
2016	39.49363049	33.87158652	1.326349102	3.137220785	17.88215847	35.15	4.952941176

2016	40.22212029	41.43533387	1.138471093	2.642951851	15.06482555	33.68	4.629141176
2016	40.96611098	28.95247152	1.475581009	2.848282099	16.23520797	32.4	6.708
2016	29.69206035	23.19548767	1.564617838	2.731401219	15.56898695	31.9	5.072941176
2016	35.81250562	31.98161957	1.364474385	2.84563595	16.22012491	35.45	4.761858824
2016	44.32422369	33.9190386	1.334618243	2.705102149	15.41908225	32.45	4.420705882
2016	22.82786018	25.64946531	1.820499194	3.2865998	18.73361886	31.3	4.267670588
2016	30.69578218	29.07722517	1.205705176	2.763312045	15.75087866	31.6	3.447529412
2016	40.0671584	35.73610251	1.486374626	2.354873282	13.4227777	29.1	2.970823529
2016	32.10268113	30.51165183	1.098455309	2.760269922	15.73353856	30.54	3.627294118
2016	36.88794576	33.00128536	1.483663499	3.103838435	17.69187908	26.4	
2016	31.82309539	38.39310669	2.176462259	3.051160248	17.39161341	30.15	2.536470588
2016	30.38331285	25.03930201	0.873758123	2.660203004	15.16315712	34.6	5.103294118
2016	32.17136469	26.32217822	1.631779074	2.719704439	15.5023153	30.7	3.434352941
2016	44.84843233	37.36501082	0.850132532	2.579008378	14.70034776	34.75	3.83
2016	54.2055401	41.64629346	1.391703716	3.390926226	19.32827949	32.95	4.298176471
2016	30.3770068	24.4526146	1.378421813	3.125408981	17.81483119	34.65	
2016	32.16145136	29.73137005	1.436419769	2.923508127	16.66399632	35.05	3.561764706
2016	35.64171278	24.80544107	1.544006593	2.885106839	16.44510898	32.45	7.486117647
2016	30.93624975	36.82596204	0.844815662	2.856522658	16.28217915	33.84	5.381623529
2016	28.87797403	34.77561781	1.505418977	2.942849417	16.77424168	34	4.255058824
2016	32.1860807	26.66585544	1.930377093	2.751763474	15.6850518	32.25	3.509647059
2016	36.15759954	32.04970611	1.54816699	3.030183913	17.27204831	30.66	4.638117647
2016	34.90213265	25.84407056	1.495954816	2.612024276	14.88853838	30.1	3.064470588
2016	37.81333077	35.33473159	1.318473465	2.290286165	13.05463114	38.25	3.636941176
2016			1.383416182	2.594773437	14.79020859	32	2.660705882
2016	50.60009806	47.35605187	1.178187767	2.839334046	16.18420406	36.7	3.012705882
2016	30.44212822	28.29961161	1.252153817	2.491869329	14.20365517	34.8	6.280235294
2016	34.91874301	29.47344024	1.414907574	2.321765373	13.23406263	32.85	5.651670588
2016	32.57454261	23.34251122	1.653308242	1.962303888	11.18513216	32.6	4.556647059
2016	34.51359502	36.12543277	1.754729355	2.486820904	14.17487915	34.95	3.250823529
2016	41.94162488	49.63259153	1.269772382	3.214435172	18.32228048	33.46	3.754729412
2016	24.66802116	38.93698592	1.158905001	3.382998043	19.28308884	31.35	5.083294118
2016	28.27191562	24.85198143	2.054008658	2.362664057	13.46718512	32.25	

2016	34.95712621	28.30316249	1.529721389	3.278263594	18.68610249	31.1	5.041835294
2016	37.29907382	34.57884006	1.504222598	2.640817632	15.0526605	32.1	4.774517647
2016	31.60088812	28.1182623	1.084257976	2.563766352	14.61346821	35.3	5.251152941
2016	22.42262696	23.90249231	1.076354949	2.639486624	15.04507376	33.35	4.054258824
2016	34.5276897	21.48860317	2.100006947	2.743938682	15.64045049	30.05	6.281305882
2016	36.16791597	27.96609808	1.31424901	2.735286105	15.5911308	34.05	5.659294118
2016	34.31413966	36.58807328	1.366963607	2.331849213	13.29154051	30.65	5.039576471
2016	34.21848261	34.33413171	1.189062197	3.035751166	17.30378165	36.05	4.318964706
2016	33.37405587	20.14130625	1.142518729	2.486411464	14.17254534	34.85	2.575764706
2016	37.26113583	30.98820463	1.224189234	3.006763282	17.13855071	35.4	3.406117647
2016	37.95926978	46.67179666	1.484089546	1.961150384	11.17855719	36.2	3.023058824
2016	35.386557	38.34592593	1.098284299	2.521649142	14.37340011	39.55	3.308282353
2016	37.52697421	43.76327703	1.494953939	3.028764477	17.26395752	39.2	3.861647059
2016	31.03692039	30.52188873	1.319378891	3.431512125	19.55961911	34.95	2.909176471
2016	32.65310149	29.09784048	1.474757896	3.162892742	18.02848863	34.05	4.370117647
2016	32.02496567	33.33943008	0.914336693	2.863606905	16.32255936	37.35	3.965176471
2016	33.10276171	26.01842271	1.080031023	1.851218108	10.55194322	35.8	3.447529412
2016	22.08584	21.92375185	1.193605565	2.115729072	12.05965571	33.55	4.833176471
2016	39.23224014	27.19132879	1.680847876	3.331354228	18.9887191	33.2	5.949741176
2016	37.14269484	32.55379039	1.751659344	3.05058602	17.38834032	36.5	6.059929412
2016	48.71040041	40.03841319	1.646694571	3.134532767	17.86683677	38.25	5.518117647
2016	38.22868186	32.37820754	1.334467652	3.411619356	19.44623033	38.95	5.904
2016	45.82005884	42.04180953	1.546152293	2.862708577	16.31743889	38.55	4.4574
2016	26.85659251	29.50438213	1.648165916	2.338538457	13.32966921	36.8	6.886741176
2016	29.00633771	38.38174994	1.347568595	2.657226371	15.14619031	36.3	4.576
2016	12.63733715	16.72978435	1.545592761	1.926507695	10.98109386	37.3	3.525129412
2016	21.01116056	23.30338667	1.217283721	2.051206951	11.69187962	33.9	3.294823529
2016	34.01917006	31.58030964	1.673501795	2.314835682	13.19456339	32.1	
2016	35.45881084	29.67621674	2.370775324	3.042642606	17.34306285	33.64	3.230117647
2016			1.396810277	2.584746609	14.73305567	36.55	3.252705882
2016	10.15680085	19.39572622	1.233232528	3.281060474	18.7020447	35	3.872
2016	28.66703397	28.97375583	1.523627788	2.703597149	15.41050375		2.878117647
2016	37.09128579	29.11400058	1.317377202	3.318614549	18.91610293	39	4.125882353

2016	39.80474671	28.49162879	1.313557254	2.704398962	15.41507408	41	3.518776471
2016	33.02188555	39.3669907	1.470264413	3.469490291	19.77609466	40.68	4.141176471
2016	32.08995604	22.06805504	1.661403111	2.134685574	12.16770777	36.5	
2016	38.73216913	33.14863398	1.326624307	2.850454829	16.24759253	33.3	3.975858824
2016	36.68570166	30.46920895		3.115616943	17.75901657	35.64	3.054117647
2016	35.3710474	31.3523269	0.907929334	2.390513648	13.62592779	36.65	4.138588235
2016	33.12041424	30.64065204	1.629980774	2.112991085	12.04404919	36.2	4.448294118
2016	35.80192624	31.39449125	2.239872755	3.170255168	18.07045446	33.7	
2016	31.73093884	32.2207542	1.52711123	2.994622601	17.06934883	40.95	
2016	33.5699055	34.7221777	1.432188124	3.85894609	21.99599271	42.3	4.717411765
2016	34.67025313	27.61555907	1.373197099	2.204712823	12.56686309	38	4.155105882
2016	19.72979642	25.58238516	1.322697058	2.373299242	13.52780568	32.85	3.657047059
2016	28.31536018	30.58233703	1.670186943	2.522055105	14.3757141	34.5	4.151529412
2016	30.52132429	32.16859842	2.205937203	2.828907921	16.12477515	31.95	5.083294118
2016	34.54206348	27.52876808	1.415754254	2.872277411	16.37198125	35.2	5.116011765
2016	41.70909171	38.72899397	0.933160413	2.78011964	15.84668195	34.15	3.406117647
2016	44.45913251	41.48945941	1.115340503	2.595230216	14.79281223	33.55	6.008141176
2016	34.5222257	33.21484982	1.35246636	2.737761379	15.60523986	35.75	
2016	36.23333922	33.39906441	1.514045145	2.339822573	13.33698867	34.85	5.295435294
2016	30.11844829	20.926135	0.87866724	2.726427184	15.54063495	31.85	4.294470588
2016	42.39996203	33.45155475	1.409357433			28.75	5.034211765
2016	39.9171422	40.2161738	1.445893247	2.969341723	16.92524782	30.68	3.921317647
2016	29.69828967	23.61036307	1.586839905	2.575713003	14.68156412	32.9	3.085176471
2016			1.413301572	2.583370384	14.72521119	29.65	4.329223529
2016	35.45748844	31.76422936	1.384137158	2.87973583	16.41449423	34.7	5.1646
2016	27.60839246	24.70251553	1.634656832	2.931342527	16.7086524	34.45	3.902823529
2016	34.31437651	26.86724487	1.296078806	3.013889961	17.17917278	31.35	5.155623529
2016	43.59796066	33.80380912	1.564226592	3.031746879	17.28095721	31.8	3.002352941
2016	40.23982998	25.84095469	1.743509688	2.84062735	16.19157589		3.830588235
2016	34.82664044	28.83409189	1.335676296	2.854891593	16.27288208	32.25	5.113576471
2016	41.86896629	29.23191222	1.363641741	3.031862095	17.28161394	29.2	4.908341176
2016	36.28872613	27.35097487	1.212075563	2.803478003	15.97982462	31.6	3.654588235
2016		37.09040761	2.172837514	3.088593545	17.60498321	35.75	4.146352941

2016	30.03775175	33.35186941	1.070537413	2.770461845	15.79163252	34.48		2.913894118
2016	35.82763436	30.55068631	2.027083769	3.320559264	18.92718781	32.4		
2016	31.46017951	32.01153685	1.311513524	3.011777193	17.16713	32.85		4.487152941
2016	42.91173254	44.60353979	1.237570438	2.65845695	15.15320462	32.15		4.613929412
2016	47.39761051	29.58728698	1.412269158	3.275000076	18.66750043	35.1		5.158588235
2016	30.26987844	26.67550405	1.156473833	2.344820327	13.36547587	39.65		2.339764706
2016	40.66425233	37.40810229	1.357408308	3.239462006	18.46493343	34.05		2.702117647
2016	40.95108354	43.55538819	1.467664591	3.040601261	17.33142719	37.6		4.099764706
2016	64.74930194	54.96726575	1.285569579			33.5		4.92
2016	42.08220301	35.60659646	0.90302554	2.651497521	15.11353587	36.25		5.440729412
2016	33.54170498	28.72025624	1.879588027	3.457831096	19.70963725	35		3.965176471
2016	31.72894494	30.1267463	1.080166026	3.211000735	18.30270419	30.7		4.408705882
2016	39.84934468	28.25901885	1.747189954	2.871535936	16.36775483	31.35		
2016	28.80118464	28.35735725	1.484631732	2.433612066	13.87158877	32.3		3.189741176
2016	28.26556087	30.56139856	1.056073157	2.505721796	14.28261424	38.4		4.6116
2016	60.3543207	60.12607312	1.004641332	2.709728494	15.44545241	35.8		5.712164706
2016	33.24967787	31.47779564	1.253442533	2.368512469	13.50052107	35.45		7.16
2016	38.04691764	30.50563337	1.219935462	3.033165208	17.28904168	36.95		3.962164706
2016	43.48564453	38.70363594		0.184182977	1.049842968	32.6		6.129317647
2016	35.37734881	39.19983012	1.805269258	2.373328133	13.52797036	38.2		4.627764706
2016	25.57519564	17.76727035	1.815528851	2.777262911	15.83039859	26.35		3.919305882
2016	29.86994985	35.3889944	1.022474127	2.659134143	15.15706462	26.3		
2016	35.43130671	33.8415009	1.721602782			26		2.619294118
2016	41.18721843	37.44717705	1.28609956	2.863989245	16.3247387	30.2		
2016	26.45302159	23.7170768	1.743181123	3.012270673	17.16994283	31.1		4.068705882
2016	27.70193877	29.97447464	1.878671666	2.357309447	13.43666385	32.45		4.058352941
2016	35.11330535	25.02211722	2.322454921	2.327482673	13.26665123	32.75		7.698352941
2016	23.74014987	18.77126219	1.598330592	2.847511831	16.23081743	32.85		6.804494118
2016	28.3952181	35.26744923	1.417003609	2.820725066	16.07813288	34.65		4.5396
2016	36.25714228	33.70055185	1.661522102	2.391838747	13.63348086	35.75		6.187694118
2016	30.53720575	24.72168479	1.867401838	2.978369156	16.97670419	34.1		4.406411765
2016	37.10358154	34.81570363	1.335361498	2.889548703	16.4704276	31.3		4.845176471
2016	38.23330753	29.69186798	1.758737237			24.15		7.478941176

2016	39.95938772	28.82291836	1.751882373	2.698283519	15.38021606	29.8	3.536682353
2016	29.70570247	21.09846927	0.964182599	2.918393608	16.63484357	28.45	4.167494118
2016	34.583701	25.95141104	1.482553506	2.562127745	14.60412815	31.75	4.327529412
2016	36.48004375	26.58692847	1.231027765	2.614259077	14.90127674	31.1	5.573035294
2016	49.14029404	34.57331988	2.087601764	2.366353846	13.48821692	33.35	4.524235294
2016	26.26836613	23.85602301	1.571371551	2.802706042	15.97542444	28.55	4.299858824
2016	31.86696745	22.03880039	1.700236677	2.713121818	15.46479437	32.65	3.662764706
2016	25.08929015	27.78131807	1.779052674			32.25	6.087411765
2016	32.15121798	30.93175746	1.271745863	2.868429859	16.3500502	30.1	4.997176471
2016	35.74054594	28.61279066	1.427275635	2.372457802	13.52300947	31.5	3.064470588
2016	34.87004451	26.63759091	1.473945585	2.682840868	15.29219295	32.75	3.934117647
2016	47.85165466	30.59719747	1.892095817	2.784796571	15.87334045	31.3	4.066835294
2016	36.19555706	41.99005017	1.813571255	3.175726109	18.10163882	31.75	
2016	41.55655157	29.93759925	1.621684987	2.304805398	13.13739077	29.45	3.965176471
2016	40.82360297	34.24461818	1.449809822	2.19023227	12.48432394	31.7	3.830929412
2016	49.43361148	40.54824449	1.631783067	2.258665206	12.87439168	30.46	4.955011765
2016	36.82412159	32.35731615	1.544263723	3.095262128	17.64299413	28.2	4.244705882
2016	30.88813781	30.53995189	1.981589429	2.595901094	14.79663624	32.65	7.203011765
2016	45.80183947	41.4863209	1.592405177	2.427694235	13.83785714	31.9	5.546894118
2016	32.56529838	31.9920786	2.204815585	2.477114291	14.11955146		4.609129412
2016	26.71331382	23.94078919	1.665599659	2.923138971	16.66189213	29.4	6.392470588
2016	23.42020052	22.68204308	1.268943488	2.773875355	15.81108952	29.4	4.354729412
2016	33.63326667	38.87868662	1.793214337	2.761536981	15.74076079	30.3	5.083811765
2016	29.82249571	24.20006329	1.918778731	2.601708497	14.82973843	34.9	6.213705882
2016	31.27154674	18.86447161	1.445293569	2.364978632	13.4803782	34.45	7.413741176
2016	42.89668761	32.4751402	1.423670837	2.764115843	15.7554603	30.6	6.169976471
2016	24.34014325	27.76375723	1.516740974	2.971702765	16.93870576	32.1	2.766705882
2016	30.24723168	32.37066679	1.500499314			34.25	3.922941176
2016	33.81262855	38.1519851	1.934085472	2.884136636	16.43957882	35.1	2.971294118
2016	56.3241863	41.70271792	1.54057386	2.644343687	15.07275902	34.15	6.781882353
2016	33.19545305	28.04508718	1.045109823	2.572984916	14.66601402	36.2	4.119317647
2016	36.42225032	33.8775238	1.354959566	2.285177496	13.02551172	37.5	3.733458824
2016	26.94151199	24.68048684	0.904882124	2.382953276	13.58283367	33.6	5.966752941

2016	33.6525318	39.15096431	1.949374024	2.957946421	16.8602946	33.2	5.689905882
2016	37.8538472	23.50105476	1.431351576	2.600343761	14.82195944	33.8	4.988235294
2016	27.78324282	33.20381982	1.524057734	3.016208318	17.19238742	39.8	5.421647059
2016	35.87758191	40.77539986	2.131418314	3.269425966	18.635728	38.6	5.392705882
2016	33.62311123	30.94778345	0.745273119	2.585509793	14.73740582	39.65	6.366588235
2016	33.87680097	34.94036358	1.170507795	3.052159434	17.39730878	32.25	5.653176471
2016	26.53968344	29.59448307	1.873259263	2.644050065	15.07108537	32.8	5.167847059
2016	36.5240189	38.13242496	1.194631698	3.043108147	17.34571644	31.95	5.693294118
2016	36.14270593	29.93454531	1.430377028	3.482711411	19.85145505	29.95	4.987529412
2016	30.3780467	32.82651279	1.812258178	3.43378675	19.57258447	32.7	3.302588235
2016	32.55105708	31.6404598	1.657663003			31.8	
2016	39.77384018	36.54138447	0.853835806	2.40296491	13.69689999	37.45	5.159858824
2016	32.90116173	36.11738735	1.253973542	2.947112892	16.79854348	35.4	4.317176471
2016	27.68773109	29.45314725	1.1706146	2.684350147	15.30079584	33.46	5.381741176
2016	44.2195423	39.78614461	1.455336826	2.909089271	16.58180884	32.25	5.296235294
2016	35.05661005	38.72082908	1.116564679	2.924324319	16.66864862	38.35	3.147294118
2016	29.12679829	34.50492988	1.198033215	2.639897582	15.04741622	36.8	3.8148
2016			1.655794672	2.407729789	13.7240598	38.3	3.326305882
2016	32.87317163	34.43610891	1.840699526	2.584406295	14.73111588	35.6	3.209411765
2016	46.10237792	36.26634877	1.529797456	3.633469855	20.71077817	37.7	
2016	28.36107727	26.39740359	1.149039205	3.141699725	17.90768843	29.3	5.215435294
2016	35.87915407	32.86641943	1.469478093			29.35	4.016941176
2016	34.86779659	36.39694776	1.243497857	2.960240574	16.87337127	31.85	3.749282353
2016	45.98417131	36.85433546	1.305677813	3.137168056	17.88185792	36.25	5.662823529
2016	33.14469695	27.02037849	1.004643045	2.906101995	16.56478137	32.15	4.813882353
2016	30.65530468	27.9491237	1.304570457	3.026890722	17.25327712	33.4	5.653270588
2016	25.46298497	29.97120247	1.611008544	2.287780647	13.04034969	37.35	4.679529412
2016	34.44440304	23.62900195	1.574929176	2.651963954	15.11619454	36.6	5.207529412
2016	37.9536498	36.12818863	1.363709263	2.879826232	16.41500952	36.1	
2016	33.81356064	27.73548258	1.755139512	2.705887372	15.42355802	40.45	5.083929412
2016	30.02546996	24.51897498	1.034847136	2.704657545	15.416548	39.8	5.493529412
2016	36.24265951	28.64098249	1.778237115	2.548992614	14.5292579	36.7	5.358458824
2016	33.8799015	26.05622675	0.956055163	2.721287742	15.51134013	34.7	4.533105882

2016	39.34663696	27.92719315	0.916374318	3.007374699	17.14203578	30.55	7.537694118
2016	24.020866	19.97657676	1.553505149	2.68973814	15.3315074	31.45	4.058352941
2016	31.88333457	27.42104	1.433969258	2.14367065	12.21892271	30.25	7.008376471
2016	33.0953011	30.10752318	1.590867065	3.121713367	17.79376619	34.25	5.450705882
2016	30.30376903	39.55424754	1.195811214	2.888738787	16.46581109	33.46	7.093223529
2016	32.75634509	28.80070446	1.542219187	2.631426023	14.99912833	29.85	5.902094118
2016	32.60692966	27.01705427	1.444505238	2.789916422	15.90252361	31.65	3.033411765
2016						30.8	3.561411765
2016	35.75709073	32.30917601	1.773208773	2.102062109	11.98175402	33.9	5.875658824
2016	41.3775183	30.56510118	1.462836119	2.914330154	16.61168188	34.35	5.403635294
2016	41.56899906	35.90915642	0.862475558	3.485621205	19.86804087	31.35	5.385223529
2016	31.41621295	29.09260934	1.518383971	2.872938845	16.37575142	32.35	5.6406
2016	25.38615482	23.27808532	1.58954713	2.685790097	15.30900355	29.55	6.514635294
2016	39.16721005	42.02456175	1.448490835	2.842207738	16.20058411	27.05	4.694117647
2016	26.17103516	28.64919569	2.016331546	2.433105327	13.86870036	26.9	
2016	32.37146852	25.12193293	1.822718879	2.052130006	11.69714104	27.1	5.692235294
2016	29.86865728	23.42169297	1.61620466	2.875201379	16.38864786	30.65	3.602823529
2016	30.43606315	26.49179299	1.570513381	3.218924439	18.3478693	29.1	4.936658824
2016	32.95189485	29.71335219	1.270800287	2.458348745	14.01258785	30.64	3.354352941
2016	29.60470182	35.03696395	0.893940732	0.25228131	1.438003468	30.5	4.141176471
2016	34.47666861	26.51151379	1.1879514	2.748567025	15.66683204	33.95	4.408705882
2016	40.53474413	35.9920622	1.554669543	3.23673384	18.44938289	34.4	4.244705882
2016	34.49906073	30.11065739	0.792683729	3.014342786	17.18175388	36.45	6.463058824
2016			1.45596586	2.381150888	13.57256006	32.8	5.389741176
2016	49.73895447	30.17644589	1.366958102	2.801611274	15.96918426	31.84	3.938141176
2016	31.85234709	27.3896489	1.204932955	2.493701843	14.21410051	30.56	5.363682353
2016	32.02523074	26.8814515	0.94853626	2.493618462	14.21362523	35.85	
2016	38.40353967	37.18424427	1.305366403			30.4	4.335305882
2016	71.16176432	32.71600524	1.495051994	2.76008363	15.73247669	33.76	4.814117647
2016	37.44905328	29.56592435	1.239760291	2.22276809	12.66977811	31.25	5.474964706
2016	39.4017269	29.63009123	1.458534496	2.864383966	16.32698861	28.05	4.679529412
2016	36.73279822	37.23626202	1.339492206	2.44465135	13.93451269	31.8	5.9568
2016	31.85136006	24.66762776	1.561985662	2.922986209	16.66102139	33.95	4.299435294

2016	28.93041292	28.02545685	1.289151065	2.045645641	11.66018015	33.4	3.582352941
2016	34.55252091	37.52438544	1.858428839			32.3	3.623529412
2016	35.74149184	33.50422267	0.797604341	3.145950443	17.93191752	30.4	6.476141176
2016	31.58814119	25.99028197	1.428007916	2.644769523	15.07518628	32.2	3.778823529
2016	34.6214759	28.75917562	1.225937088	2.184330634	12.45068461	27.3	4.099764706
2016	39.76834933	27.92953813	1.918208798	2.768247975	15.77901346		2.980941176
2016	36.59225498	23.45707312	0.986337565	3.250941616	18.53036721		1.746117647
2016	32.17453116	35.51659951	1.375393496	3.003924437	17.12236929		3.366823529
2016	30.46519068	21.76109725	1.121192727	3.191535689	18.19175343		4.013176471
2016	43.28318352	49.30976986	1.350950063	3.063993982	17.4647657		3.791294118
2016	41.62014663	25.53267543	1.383786663	2.655041779	15.13373814		4.592
2016	32.47455185	32.34037945	0.794777043	2.672808156	15.23500649	35	4.720941176
2016	30.2909341	28.69677532	1.10338802	2.878491446	16.40740124	32.75	4.006588235
2016	29.02181067	28.92524179	1.06111053	2.461197186	14.02882396	32.7	
2016	26.10747114	23.8065404	1.29640063	2.363787873	13.47359087	32.35	4.141176471
2016	27.91914053	20.91919794	1.672959386	2.756319936	15.71102364	28.9	3.168
2016	27.98344733	23.88289453	1.031562495	2.957358219	16.85694185	32.1	2.764235294
2016	33.20364965	22.50497753	1.613781571	2.960858596	16.876894	30.75	4.939082353
2016	30.23833361	26.00328433	2.137664939	2.840702282	16.192003	31.25	3.107294118
2016	31.40976794	25.36344648	1.446105606	2.046784244	11.66667019	27.95	5.109458824
2016	27.4649318	29.04922101	1.461416457	2.287105721	13.03650261	34.15	4.379764706
2016	38.60990178	27.30854927	0.867888076	2.370133487	13.50976087	31.45	4.820823529
2016	35.97297934	31.88868483	1.498236282	2.883382048	16.43527768	33.55	4.777070588
2016	32.94760937	25.2872145	1.365728425			26.25	6.289882353
2016	34.68668236	29.21955823	1.540581332	2.492112209	14.20503959	34.05	3.622823529
2016	31.08125507	43.63215992	1.442413775	2.578824305	14.69929854	34.65	
2016	22.93411922	22.26951336	1.770769281	3.023201871	17.23225066	36.1	4.632647059
2016	33.44700292	19.41633472	0.848120407	2.701394376	15.39794794	34.6	3.426823529
2016	42.1543239	24.16100616	1.468780217	2.686246615	15.31160571	34.25	3.696
2016	37.60478348	32.10297102	1.316927903	3.458039321	19.71082413		3.222117647
2016	30.27511337	27.38566744	0.986646796	2.930190687	16.70208691		2.807294118
2016	39.75106664	38.86401204	1.346945739	2.46831498	14.06939539		2.392470588
2016	30.8864191	31.44488983	1.736005935			34.55	6.911964706

2016	24.99812837	21.37243188	1.7000994	2.046711073	11.66625312	39.45	4.358588235
2016	24.57999058	31.8605382	1.438960296	2.916760019	16.62553211		4.188964706
2016	21.8560643	17.24937233	0.959255018	2.720432655	15.50646613	32.05	
2016	32.37763405	28.79062613	1.406712133	3.575564754	20.3807191	31.9	3.7796
2016	34.27620381	28.43206879	1.514673849	2.577597515	14.69230583	31.76	3.478588235
2016	42.20250619	40.44150489	1.164474768	2.747957391	15.66335713	34.24	2.952
2016	35.84561681	28.60949747	1.275724784	3.237636404	18.4545275	33.95	3.416
2016	44.56491769	41.90926831	1.694993598	3.712966137	21.16390698	34.7	3.743058824
2016	23.05740033	22.69703229	1.215436248	3.030958592	17.27646398	35.6	5.897623529
2016	26.01048494	24.37884397	1.407560926	3.072809018	17.5150114	34.25	5.228388235
2016	38.06423134	38.24791092	1.673277909	2.569359252	14.64534773	30.8	4.172235294
2016	25.82657993	20.76894578	1.799476652	2.883240806	16.4344726	32.1	4.670352941
2016	30.91777201	29.67246611	1.52903085	2.540053828	14.47830682	31.65	6.174070588
2016	36.6994024	37.1779676	1.791873984	3.083978666	17.57867839	27.05	5.804047059
2016	37.17200396	25.67650038	1.362888658	3.295553506	18.78465498	32	4.168329412
2016	32.60800547	24.7155517	1.426890826	2.62277561	14.94982098	35.1	5.268811765
2016	43.06620075	36.91196599	1.831235144	3.038488133	17.31938236	32.75	3.230117647
2016	33.65144523	23.65415588	1.351040039	2.839730621	16.18646454	32.55	6.537882353
2016	35.95695245	33.43088663	1.54003089	2.465263114	14.05199975	30.55	3.727058824
2016	32.36942823	23.63627286	1.170183234	2.585822914	14.73919061	35.15	5.569882353
2016	23.34687617	23.1200317	1.340606311	2.923428108	16.66354021	26.8	5.344
2016	30.8562848	27.76693939	1.333874243	2.975418968	16.95988812	32.8	5.557764706
2016	34.87259897	33.63658578	1.053385278	3.05159976	17.39411863	30.4	6.056470588
2016	36.17373041	22.04495828	1.395739756	3.060142338	17.44281133	34.65	4.337882353
2016	36.58318696	24.10300759	1.096179318	2.850353811	16.24701672	32.35	3.799529412
2016	28.13197365	19.39719175	1.535428631	2.548775653	14.52802122	26.3	
2016	34.79291653	30.17349942	1.352716884	2.778889821	15.83967198	29.4	4.224858824
2016	30.08351983	25.80472805	1.711982092	3.585466047	20.43715647	35.3	3.360882353
2016	29.07916015	23.67955689	1.370715038	2.539126967	14.47302371	32.4	3.666023529
2016	35.69258602	27.41932282	1.759965223	2.863680704	16.32298002	25.5	4.780082353
2016	30.5091482	34.60173326	1.683857147	2.891691587	16.48264205	29.65	3.903058824
2016	46.1700465	35.9658371	1.506859102	2.911424574	16.59512007	31.75	4.436364706
2016	28.19879471	25.51372781	1.230489633	2.529809302	14.41991302	31.05	4.649035294

2016	35.63404688	23.70331128	1.7434868	2.451496115	13.97352786	36.5	5.579105882
2016	33.375201	29.53989056	1.672998671	2.352089633	13.40691091	29.5	4.725917647
2016	31.67983804	23.52200766	1.487330161	2.050917746	11.69023115	27.6	3.033411765
2016						27.4	3.913411765
2016	34.49606909	29.51694594	1.972025388	2.731731705	15.57087072	25.3	3.737411765
2016	30.81795425	30.88768495	1.95482304	2.476384486	14.11539157	35.65	5.898070588
2016	27.25861106	23.93657226	0.980558079	2.476239747	14.11456656	34.95	4.068705882
2016	33.19733777	26.27882081	2.045295454	2.863666589	16.32289956	32.6	4.462305882
2016	28.97888701	21.65578172	1.594992161	2.693921602	15.35535313	33.6	4.968235294
2016	35.28178601	30.69541155	1.602287875	2.214806039	12.62439442	32.55	3.716705882
2016	27.7725553	21.58077408	1.16592126	2.773542197	15.80919052	33.3	3.668894118
2016	26.44465079	30.38476861	1.599836871	2.685373383	15.30662828	28.2	5.083941176
2016	31.79720226	29.05257183	1.166335718	2.257030301	12.86507271	24.6	3.985882353
2016	26.89396495	23.38605291	1.247475526	3.059698849	17.44028344	30.8	4.089411765
2016	23.2348457	17.92270884	1.688995332	2.925372349	16.67462239	34	3.716705882
2016	31.03405714	26.10841276	2.699719923	2.149316758	12.25110552	28.85	
2016	29.88002781	28.43306345	2.069670842	2.223883448	12.67613565	33.4	3.758117647
2016	25.65532537	26.34060842	1.272047001	2.335617319	13.31301872	28.05	4.072670588
2016	34.07469305	33.01084585	1.494064604	3.237504403	18.4537751	24.15	2.826352941
2016	28.84089522	32.84931283	1.778808841	3.326482207	18.96094858	25.15	4.555294118
2016	28.57186069	33.3824127	1.532289422	2.679069011	15.27069336	30	5.282635294
2016	38.9877441	38.90079973	1.638884456			27.6	4.182588235
2016	30.28545596	38.68404679	1.402142099	2.923472641	16.66379405	28.65	3.116235294
2016	28.3569313	26.77052025	1.64003826	3.217587586	18.34024924	27.2	3.009882353
2016	28.50644389	32.28777163	1.629077069	3.194301667	18.2075195	28.15	3.312941176
2016	26.49035121	26.96631884	0.816565409			28.05	3.437176471
2016	30.44925979	31.12654501	1.070368909	3.000992721	17.10565851	29.6	
2016	34.97099904	33.56348717	1.643405361	2.922536224	16.65845648	24.9	3.292235294
2016	31.43902467	33.01972379	1.792123167	2.927053926	16.68420738	28.9	4.689882353
2016	32.78478549	33.98568102	1.623966577	2.73607386	15.595621	28.4	5.065176471
2016	30.03809871	27.33357356	1.209343232	2.913274096	16.60566234	27.9	6.011435294
2016	37.13379072	35.75414875	1.401772238			28.75	3.790964706
2016	27.95181631	55.31159211	1.641388126	3.214963027	18.32528925	31.2	2.577882353

2016	33.8280907	34.68842298	1.226773227	3.158416623	18.00297475	31.15	2.64
2016	32.00723193	38.23402376	1.783927217	3.972052433	22.64069887	28.6	3.903058824
2016	37.74110309	28.03255129	1.910308495	2.846971193	16.2277358	27.95	4.807905882
2016	31.06756127	27.56103799	1.813843142	3.123175383	17.80209968	30.5	4.076047059
2016	31.54914101	33.96445204	1.48814074	2.651544682	15.11380469	34.3	3.406117647
2016	32.95477519	31.25990029	2.013814769	3.016266656	17.19271994	30.7	4.431694118
2016						34.05	3.230117647
2016	42.07428352	29.45249202	1.587942068	3.361020722	19.15781811	30.45	4.410352941
2016	24.53604589	27.36369173	0.701027463	3.472096313	19.79094899	27.4	3.518988235
2016	37.37617328	28.58880731	1.163657235	3.249522404	18.5222777	24.4	3.7884
2016	33.51538126	31.21789261	1.378407213	3.105683487	17.70239587	29.65	3.815364706
2016	30.18608268	28.62216431	1.490013882	2.264564132	12.90801555	32.15	5.012247059
2016	34.37067055	34.09592177	1.027972462	2.660028314	15.16216139	29.8	5.456411765
2016	36.36081381	33.55877503	1.459970144	2.548637862	14.52723581	30.4	4.731294118
2016	26.69374632	30.79381401	1.736956455	2.606768246	14.858579	28.75	4.054764706
2016	31.47422078	27.63995362	1.364526798	2.451137833	13.97148565	30.3	5.145411765
2016	36.32186783	35.87292645	1.931568774	2.510288296	14.30864329	28.1	4.586352941
2016	33.52394875	26.77428238	1.065774087	2.978199306	16.97573604	32.7	4.062494118
2016	33.72179228	27.95687969	2.049486332	3.373070868	19.22650395	31.55	4.442070588
2016	39.71046432	35.66904428	1.431658563	3.055531664	17.41653049	26.45	4.586352941
2016	2.09	0.657	1.28743535	2.956636049	16.85282548	28.4	2.847058824
2016	28.26432855	20.2786413	1.176364552	2.013014923	11.47418506	30.9	3.292235294
2016	31.56184102	24.55715171	1.368079133	2.492516548	14.20734433	28.5	3.385411765
2016	32.5325781	31.32949609	1.512502988	2.535927396	14.45478616	35.45	2.045176471
2016	39.93691021	45.11714474	2.211286746	2.921741224	16.65392498	30	2.360470588
2016	34.23792063	33.62756429	1.913137135	3.176454278	18.10578938	32.7	3.148847059
2016	40.43481238	32.44615368	1.341346078	2.435694548	13.88345893	31.6	3.446470588
2016	27.31075794	31.34263188	1.321159129	3.320948775	18.92940802	32.4	4.016941176
2016	33.94376927	35.0673997	1.138224114	2.512126116	14.31911886	34.4	4.209247059
2016	38.89236412	29.13500722	0.966295074	2.311366294	13.17478788	37.45	2.964823529
2016	41.93014139	29.71945566	2.064852108	3.206083981	18.27467869	36.2	5.815588235
2016	39.2361931	34.2659148	2.053454403	2.925503349	16.67536909	33.45	4.752
2016	16.76417102	21.07292287	1.828185898	2.999326279	17.09615979	32.9	5.272517647

2016	36.7600983	32.75718708	1.547538946	3.017966378	17.20240836		
2016	45.74390873	37.41198747	1.434699781	3.339315384	19.03409769	31.45	4.016941176
2016	33.22084013	26.67038373	2.211476768	3.971752225	22.63898768	36.4	4.824164706
2016	33.81201199	29.34683781	0.675455743	3.280307034	18.69775009	34.5	2.940235294
2016	27.16831645	36.13944938	1.270193851	3.077145669	17.53973031	35.7	2.878117647
2016	31.30645199	26.58977052	1.318031052	2.816926998	16.05648389	31.65	5.518117647
2016	33.54477418	31.26461993	1.510847528	2.654661984	15.13157331	32.4	3.509647059
2016	48.23254121	56.48867118		2.764547366	15.75791999	33.35	3.954823529
2016	29.57191191	26.72277267	1.426325782	2.159532136	12.30933318	38.8	3.442788235
2016	36.52329112	37.62164144	1.599667525	2.840522843	16.1909802	32.15	5.5566
2016	27.96958031	26.88549812	1.832910474	2.911357587	16.59473825	36.8	5.521623529
2016	45.87203999	29.65970223	1.381506414	2.625520058	14.96546433	29.05	4.734941176
2016	27.2851829	28.48039095	1.022132131	2.482246238	14.14880356	34.8	5.704729412
2016			1.66503763	3.11933801	17.78022665	31.4	3.666941176
2016						36.85	
2016			1.880751968			34.6	1.666823529
2016	47.57073937	58.2012434	1.484394895	2.831699986	16.14068992	32.95	0.496941176
2016	29.17903417	26.31046978	1.234406729	2.670445226	15.22153779		3.457882353
2016			1.411348284	2.94202828	16.7695612	30.7	6.011435294
2016	34.02652323	27.39654683	0.825811705	2.401129107	13.68643591	30.2	3.354352941
2016	45.8915246	42.15401274	1.024634647	2.628001217	14.97960694	33.55	4.553788235
2016	43.12291559	35.77740894	0.687330673	3.211339957	18.30463775	28.95	5.870117647
2016	39.39658836	31.90125018	1.759135558	3.160339426	18.01393473	29.5	3.992823529
2016	33.235958	23.76825389	1.540653882	2.673129279	15.23683689	33.84	6.203058824
2016	32.90199069	28.17194371	1.611575965	1.620462572	9.236636663	30.6	
2016	40.5887327	34.79484058	1.639168164	3.112135759	17.73917382	35.95	4.251470588
2016	27.41709367	23.62907165	1.842292244	2.65856239	15.15380562	35.4	6.955529412
2016	34.60715331	30.45398147	1.399298621	2.58636609	14.74228672	29.95	3.002352941
2016	39.42390414	36.10540315	1.025633339	2.553287853	14.55374076	33.55	4.212094118
2016	41.85220374	24.5293833	1.221805438	1.976020273	11.26331556	35.15	6.029411765
2016	50.75462922	49.94474983	1.115229771	3.071847547	17.50953102	34.1	4.076552941
2016	38.44814624	35.75895207	1.197419636	2.624528864	14.95981453	34.22	5.064705882
2016	34.4170814	35.24531727	1.101752489	2.897015633	16.51298911	31.65	3.314729412

2016	49.33998089	50.17192327	1.590840302	2.966524179	16.90918782	30.65	4.120470588
2016	32.40206694	29.27185381	1.765113366	2.595486926	14.79427548	29.5	3.230117647
2016	21.09738813	26.56668123	1.533809271	2.514124005	14.33050683	36.1	4.857082353
2016	30.49282112	27.30488043	1.296265146	2.782386831	15.85960493	29.5	4.633588235
2016	34.17211783	35.46219527	1.407978876	2.65322275	15.12336968	34.6	5.425729412
2016	25.69725112	23.55597165	1.488859962	3.027732351	17.2580744	33.95	5.991670588
2016	33.54790443	32.23287203	1.330992789	2.813175569	16.03510074	29.55	4.596705882
2016	32.54994544	30.57584102	1.214233226	3.966154408	22.60708012	31.3	4.874258824
2016	28.80131302	29.91325367	1.44004553	2.046139759	11.66299663	31.25	5.170823529
2016	29.24638972	31.67883871	1.769734144	2.769059994	15.78364197	30.85	
2016	31.66107369	21.22828356	1.037367002	3.07198497	17.51031433	33.45	4.493176471
2016	30.64208099	24.23920253	1.423402188	2.52056272	14.3672075	32.85	5.713976471
2016	33.18220358	32.49844349		2.747988322	15.66353343	32	
2016	52.61061168	46.10328155	1.284768972	2.759998927	15.73199389	33.2	3.574117647
2016	34.71358887	30.49828605	1.476268103	2.822105352	16.0860005	35.25	3.502317647
2016	50.23558793	41.82170951	1.087623477	2.683335408	15.29501183	34.4	3.926352941
2016	33.75542967	31.44206977	1.113437926			32	4.229811765
2016	29.39861177	31.64704452	1.794110455	3.242071332	18.47980659	36.2	4.273647059
2016	41.64393384	38.4413994	1.756625398	3.074882073	17.52682781	34.3	
2016	40.03713283	28.35194276	1.147154389	1.050514014	5.987929878	32.45	2.981647059
2016	33.39869003	38.53658581	1.757402928	3.118315832	17.77440024	35.8	5.296235294
2016	47.09506163	41.1465437	1.302457308	2.485514441	14.16743231	38.15	3.78
2016	30.64346916	33.03078458	1.391898141	2.557683742	14.57879733	31.5	5.147258824
2016	32.83568678	35.08886274	1.825941574	2.742875816	15.63439215	36.8	
2016	26.85358137	27.74042442	1.780537785			32.4	3.364705882
2016	32.56536435	36.41155302	1.881824287	3.045100283	17.35707161	35.6	4.016941176
2016	33.24692008	29.29336959	1.070385339	2.442284735	13.92102299	35.4	4.534117647
2016	32.26031601	28.13791495	1.394503736	2.589356305	14.75933094	37.95	6.809470588
2016	38.62729104	29.02982501	2.023215125	3.467111022	19.76253283	33.25	5.616752941
2016	37.64906716	34.56020064	1.069215615	2.768066256	15.77797766	34.25	5.553823529
2016	40.46288984	37.54695261	1.081014303	2.408180391	13.72662823	37.2	6.086211765
2016	32.13382247	20.28455263	1.822351944	2.042080135	11.63985677	30.35	4.727647059
2016	33.17395767	28.70161404	0.874168199	1.969651004	11.22701072	32.35	5.626541176

2016	23.84449598	26.22643824	1.455510442	3.085550263	17.5876365	33.9	6.325082353
2016	39.4022027	31.76165127	1.202442031	3.096937018	17.65254101	30.4	5.065505882
2016	33.0002757	35.29385536	1.522360394	2.399642703	13.67796341	37.35	4.037647059
2016	38.00261465	46.25033908	1.521932405	3.143841986	17.91989932	31.7	4.793411765
2016	30.70616464	29.66231898	1.227908202	3.562146976	20.30423777	34.75	3.188705882
2016	30.78792745	25.80974711	1.44264677	2.988520058	17.03456433	32.45	6.726094118
2016	39.98151254	30.32187578	0.953690659	2.729081902	15.55576684	31.2	4.286117647
2016	29.06540707	20.94057226	0.862185795	1.943439679	11.07760617	29.65	7.630117647
2016	29.9250488	27.8187845	1.898413527	3.403314884	19.39889484	33.6	3.965176471
2016	33.0995463	25.79035628	1.440747189	3.04133252	17.33559536	36.95	4.343729412
2016	37.54377666	29.51971345	1.38980854	3.166934534	18.05152685	31.2	3.602823529
2016	37.22395273	31.61558919	1.470255605	3.006911979	17.13939828	33.3	4.296470588
2016	31.51822365	23.27440774	1.496386746	2.845118163	16.21717353	34.6	5.207529412
2016	30.86692813	29.09745714	1.870435252	3.119212868	17.77951335	30.95	5.4374
2016	40.80238074	39.71403204	1.348474934	3.077978126	17.54447532	32.1	3.923764706
2016	52.04594292	32.01657223	1.884049172	3.118493341	17.77541205	32.5	4.075470588
2016	30.81944049	25.90942704	1.266725004	3.157568357	17.99813963	32.1	
2016	30.73900569	27.52081722	1.690615023	2.912602573	16.60183467	34.95	3.116235294
2016	42.61040905	39.68933271	1.874602435	3.403993578	19.40276339	31.7	4.731294118
2016	38.14575125	35.63470732	1.390986668	3.325309556	18.95426447	37.1	4.555294118
2016						37.6	3.199058824
2016	34.09577585	24.68200294	2.922163613	3.224676376	18.38065535	39.3	3.8456
2016	49.02339752	51.04011721	1.232985241	3.243396025	18.48735734	35.65	3.126588235
2016	28.23517487	24.57705857	0.994967107	2.395539558	13.65457548	34.2	
2016	34.836167	26.36900459	1.740129981	4.178956115	23.82004985	34.95	3.644235294
2016	39.07546744	39.31982367	1.324468556	3.401388297	19.38791329	29.45	3.664941176
2016	34.30862747	28.16492026	1.719582613	2.408184396	13.72665106	33.75	2.888470588
2016	33.4256962	27.34056425	1.400349305	3.175014611	18.09758328	34.05	4.426270588
2016	30.96281914	28.00587498	1.568848234	3.041621193	17.3372408	29.15	3.406117647
2016	37.36324778	38.71915694	1.887671226			30.55	3.844094118
2016	35.69443865	28.80392167	1.484792204	3.165133009	18.04125815	31.25	
2016	39.63952121	30.91613867	1.210370484	2.934656466	16.72754186	31.4	3.333647059
2016	36.21447962	22.11634285	1.472832382	3.123776899	17.80552833	33.55	5.059764706

2016	50.40985942	49.8344732	1.464889017	3.184881358	18.15382374	32.8	4.037647059
2016	35.07706692	34.55177617	1.343120889	2.04175047	11.63797768	36.7	2.992
2016	33.61053188	28.77132042	1.425514333	2.78166752	15.85550486	34	5.412
2016	33.26443064	27.92995985	1.581103768	3.313106015	18.88470429	34.4	4.668988235
2016	31.72289365	27.9705934	1.313939691	2.607363639	14.86197274	35.55	4.119317647
2016	33.70062428	30.75856234		2.725306812	15.53424883	36.8	5.537270588
2016	36.45103489	30.71294224	1.755887546	2.464915477	14.05001822	34.45	4.386058824
2016	48.2731866	33.57111182	0.901722622	2.581124956	14.71241225	31.6	4.819952941
2016	42.70080024	31.47460159	1.227689856	3.273778473	18.6605373	30.5	4.695482353
2016	39.01692723	27.61315705	1.360859845	2.739414892	15.61466488	31.7	
2016	43.98999315	35.59167238	1.704176606	2.935058311	16.72983237	32.5	3.996235294
2016	29.28681794	23.25550385	1.173045422	3.181234632	18.1330374	35.5	3.209411765
2016	34.51410513	26.48261593	1.186536413	2.911918284	16.59793422	33.25	3.333647059
2016	35.10119571	33.6716299	1.728795723	3.14574073	17.93072216	33.8	4.126129412
2016	31.91878594	19.25472059	1.453160427	2.832080452	16.14285858	39.85	6.183823529
2016	35.60471206	33.98361906	1.761721461			36.85	3.934117647
2016	46.34961072	43.4201755	1.547083145	3.254949522	18.55321228	38.7	3.644235294
2016	32.14278477	23.88646537	1.116156534	2.999607437	17.09776239	33.05	2.194823529
2016			1.721756094			33.11	0.310588235
2016	42.25055342	46.28927031	2.03671057	2.762152868	15.74427135	32.2	0.424470588
2016	44.44878807	43.12383348	1.493552637	2.425826422	13.8272106	37.5	5.738917647
2016	40.40060068	23.94150829	1.026887439	2.899299556	16.52600747	39.9	3.095529412
2016	38.49536286	29.44893985	1.605758845	2.836676266	16.16905472	36.6	
2016	37.29999524	32.6333237	1.465525218	4.05993131	23.14160847	34.3	3.392682353
2016	47.10677668	43.6329845	2.043781613	2.021837989	11.52447654	35	3.457882353
2016	36.52762045	35.18495089	2.125815467	2.729144153	15.55612167	32.7	3.975529412
2016	27.59388764	26.73635793	1.680968923	3.064753744	17.46909634	34.65	3.975529412
2016	31.46284276	23.81196765	2.096306521	2.759138238	15.72708796	31.25	
2016	34.47665928	32.41016014	1.557302949	2.802559568	15.97458954	37.9	3.499294118
2016	33.86593869	30.46504247	1.455284991			34.1	2.6214
2016	35.51304951	24.76398516	1.649156201	3.167277933	18.05348422	37.75	3.778823529
2016	41.91815937	33.61113511	1.825005119	2.670155649	15.2198872	35.85	2.929882353
2016	42.72829688	27.09538824	1.740170061	2.722619375	15.51893044	33.2	6.170352941

2016	47.54442851	46.950906	1.558266426	0.862261727	4.914891846	30.9	3.054117647
2016	30.31843801	33.14463576	1.856393444	3.241114692	18.47435375	34.35	
2016	35.43445739	26.08409065	2.882547919	3.198994314	18.23426759	36.9	3.344
2016	33.85069404	31.66902854	2.112067489	3.66098824	20.86763297	34.45	
2016	38.34874117	28.7543609	1.892289119	2.268561457	12.9308003	36.5	4.224
2016	30.24183796	22.55072729	2.253837539	2.41149628	13.7455288	36.2	4.553411765
2016	35.76465786	25.93132936	1.354038924	3.114127442	17.75052642	35.3	6.231917647
2016	42.59154769	32.34691885	1.328596732	1.779262513	10.14179633	37.8	
2016	44.55516869	29.80415771	1.37544766	3.100702406	17.67400371	36.95	
2016	43.62837039	27.40102882	2.257843208	2.946330745	16.79408525	34.5	2.764235294
2016	36.36745235	31.77474162	1.629168334	2.713686271	15.46801175	33.45	3.271529412
2016	34.33061088	20.61057281	1.881655969	2.105601113	12.00192634	36.65	3.012752941
2016	35.60879441	31.80253777	1.28254954	2.651464255	15.11334625	37.5	3.582117647
2016	31.62329994	25.84008661	1.758441176	2.439263149	13.90379995	35.1	5.621647059
2016	40.86427735	30.8194643	1.448250951	2.31036939	13.16910552	38.2	
2016			1.325708911	3.009880791	17.15632051	40.2	6.015058824
2016	36.33695592	37.97378346	1.954730779	2.798817922	15.95326216	37.4	4.673117647
2016	39.88627881	41.44115398	1.938584157	2.504035314	14.27300129	38.15	5.185776471
2016	32.31974431	25.23910173	1.27578508	2.926152216	16.67906763	40.55	6.575435294
2016	30.8683532	28.65055252	1.680155371	2.611472964	14.88539589	44.15	3.354352941
2016	33.23680027	25.5221628	1.473575596	2.96750279	16.9147659	31.1	4.244470588
2016	30.60116281	24.43785198	1.243672621	3.097037056	17.65311122	34.75	5.247623529
2016	25.91219567	23.89219454	1.638498363	2.72969205	15.55924468	30.6	4.355529412
2016	40.69212483	34.24848325	1.24791389	2.827415414	16.11626786	39.75	4.987529412
2016	39.97475992	39.49810439	1.19480443	2.333726684	13.3022421	40.4	5.167176471
2016	38.79501826	36.73720451	1.569633876	2.438497462	13.89943554	38.2	
2016	31.51373021	27.23701885	1.446412041	2.823475476	16.09381022	35.15	4.855482353
2016	37.96932087	35.07624931	0.900804923	2.367848557	13.49673678	33.25	3.437176471
2016						35.05	3.456
2016	25.89863382	23.40448682	1.778739833	2.999088588	17.09480495	32.85	4.709317647
2016	31.77084316	38.29852031	1.397410328	2.826691811	16.11214332	38.35	5.476470588
2016	37.42404412	35.12524275	1.717595433	2.743645499	15.63877934	34.8	4.507588235
2016	30.47065453	24.69445499	1.979316703	2.523156715	14.38199327	33.95	4.115823529

2016	32.9064999	30.69826037	1.916586679	2.841138835	16.19449136	33.45	3.433270588
2016	30.68134488	31.11602409	0.935464369	2.256753396	12.86349436	34.45	4.368941176
2016	36.49591609	31.33411155		3.461185805	19.72875909	34.7	4.285035294
2016	37.05051659	38.0106644	1.881137699	3.163876122	18.0340939	36.15	4.276376471
2016	33.57553504	35.80329478	1.911149008	2.819668396	16.07210986	35.05	3.209411765
2016	37.61465842	36.6545681	1.541149927	2.778628284	15.83818122	33.7	2.913411765
2016	33.32900459	32.3534695	1.599500033	3.581610652	20.41518072	30.4	1.200941176
2016	27.13716772	43.01700408	1.653023226	3.23766565	18.45469421	31.8	3.406117647
2016	22.19873986	27.04872563	1.693479036	2.205496854	12.57133207	36.15	6.290647059
2016	34.94896196	33.84273217	1.243287824	2.378980519	13.56018896	33.7	5.586941176
2016	31.13865216	32.61662381	1.399129348	2.634906872	15.01896917	35.2	6.849411765
2016	37.33555662	30.91855559	1.807618091	2.426045411	13.82845884	37.1	3.426776471
2016	33.79783213	32.08568306	1.401879198	3.002673217	17.11523734	37.5	3.714117647
2016	38.04953546	30.5237483	1.54069308	2.838650059	16.18030534	34.5	3.457882353
2016	37.80533726	25.82516428	1.533641541	2.660781937	15.16645704	37.75	4.910352941
2016	15.00627079	25.40225257	2.180512515	3.210028613	18.2971631	37.15	3.830588235
2016	38.25012345	35.63935596	2.503085127			35.6	4.967470588
2016	38.31397009	34.00773884	1.48438766	2.31213163	13.17915029	38.3	3.281882353
2016	36.81588761	32.3544484	1.725451048	2.583175618	14.72410103	38.15	2.317270588
2016	33.05968213	30.29247797	1.702217653	2.814638178	16.04343761	33.25	3.996235294
2016	31.85265849	32.83701312	0.871262023	2.391524923	13.63169206	35.8	8.421882353
2016	30.43904519	29.03519479	1.196208749	2.757498101	15.71773918	38.25	6.198729412
2016	34.61873536	37.43973614	0.473550698	2.917549187	16.63003037	38.05	8.043576471
2016	28.64797887	21.71416672	1.483786923	3.166657233	18.04994623	34.25	5.315529412
2016	30.02314581	18.85870262	1.417332352	2.515237971	14.33685643	35.4	3.470352941
2016	42.65557095	40.89347156	1.37481786	2.410751164	13.74128164	34.8	4.281247059
2016	35.887243	31.20574807	1.561791191	2.852886586	16.26145354	37.1	
2016	41.13365077	35.63143625	1.737220518	2.873293111	16.37777074	36.9	4.727058824
2016	39.51602117	32.12451652	2.005452913	3.033142002	17.28890941	34.3	3.977882353
2016	26.32498452	27.91693803	1.13409014	2.773173653	15.80708982	39	6.470588235
2016	34.75242772	32.5457578	1.589848147	2.875998817	16.39319326	39.8	5.898529412
2016	22.50244512	26.11675785	1.87014637	2.78896797	15.89711743	36	5.0898
2016	27.27228354	29.42207721	2.109432183	4.883024511	27.83323971	36.5	4.446588235

2016	32.47949998	27.53377109	1.651883128	2.977830883	16.97363603	38.05	4.508576471
2016	30.06733279	27.64046681	1.422055047	2.183002203	12.44311256	37.25	3.676235294
2016	35.28311787	45.32036942	1.652626584	2.947744469	16.80214347	36.6	3.357176471
2016	30.17324489	37.83591227	1.556147371	2.071626641	11.80827185	40.05	3.752705882
2016	40.99704764	42.71935336	1.461599499	2.547408077	14.52022604	37.25	
2016	29.71457013	34.83471412	1.509957387	2.880687499	16.41991875	32.7	3.965176471
2016	34.05477049	37.8368225	1.4905214	2.824039694	16.09702626	35.5	3.821211765
2016	31.93048002	42.3240606	0.991820675	2.511129372	14.31343742	35	4.683388235
2016	39.22987265	34.78916386	1.291103281			37.2	6.174117647
2016	34.79903493	28.56770235	2.203839859	3.392832657	19.33914615	34.9	3.457882353
2016	37.77360388	33.75385292	1.086642483	3.125129503	17.81323817	38.45	5.062588235
2016	32.90969893	28.43609399	1.564024884	2.901397997	16.53796858	41.25	4.862117647
2016	42.79658225	40.90132298	1.3005896	2.063718531	11.76319563	39.5	5.307482353
2016	25.41268051	21.41541025	1.435200884	2.856654696	16.28293177	40.9	4.099764706
2016	38.37575718	25.89901129	1.487582056	2.815567249	16.04873332	37.1	4.280941176
2016	36.18295012	28.3290468	1.943887953	2.525673781	14.39634055	36.6	3.737411765
2016	41.33299617	32.71111031	1.23551487	2.157553091	12.29805262	34.7	3.838164706
2016	27.93840196	29.65639173		3.064068929	17.4651929	39.35	2.836705882
2016	33.76351615	35.61298934	1.20792213	2.781155832	15.85258824	37.64	
2016	45.84241802	39.49509395	1.763993975	2.979369722	16.98240741	35.6	4.989082353
2016	25.63405003	24.45751436	1.599573243	2.492090393	14.20491524	35.6	4.493823529
2016	30.68584523	25.22499482	1.804680297	2.143188143	12.21617241	32.85	4.500564706
2016	25.10825469	26.69182857	1.50504864	2.233748264	12.7323651	35.5	3.790894118
2016	33.77652984	30.90879206	1.18201867	2.873400743	16.37838423	39.35	4.637847059
2016	41.94994153	35.75951672	1.489168144	2.324880562	13.25181921	39.85	5.062588235
2016			1.58434368	2.87884524	16.40941787	39.4	5.332517647
2016	32.51881144	26.08365111		2.345764522	13.37085778	32.5	5.199764706
2016	28.07068853	25.94960122	1.085483465	2.826989902	16.11384244	34.3	5.530929412
2016	41.69790541	36.09493432	0.996320332	2.735722596	15.5936188	33.2	4.313235294
2016	31.12874636	23.22131393	1.162557695	1.971005841	11.2347333	40.1	7.283529412
2016	35.40409055	34.74723726	1.060593061	2.374620938	13.53533934	42.7	6.230894118
2016	30.73963086	28.61636609	1.651484266	2.018432012	11.50506247	38.15	3.930211765
2016	33.548641	26.1679284	1.494444439	2.458463162	14.01324002	40.9	4.6656

2016	37.46616507	31.79565352	1.228839089	2.133490923	12.16089826	40.2	6.530682353
2016	34.82191546	35.02657151	1.121045719	2.74736152	15.65996066	38.05	4.574870588
2016	26.74093651	23.79484058	1.112514085	2.653681321	15.12598353	35.2	3.185494118
2016	26.24760364	25.76266337	1.000558538	2.266463104	12.91883969	33.2	5.430941176
2016	33.39437503	29.06406125	1.488124737	2.695408541	15.36382868	30.3	4.741647059
2016	28.7666918	29.18139437	1.653299387			29.95	5.393294118
2016	39.97702412	32.02771538	1.205717893	2.595590917	14.79486823	30.6	6.766941176
2016	26.10010064	23.50348823	1.405107365	2.201969162	12.55122422	31.7	3.778823529
2016	20.16099918	18.39061774	1.289534478	2.719402645	15.50059508	33.35	3.181188235
2016	39.77398037	37.22447911	1.292179372	2.299703046	13.10830736	37.35	5.051258824
2016	29.26514498	30.41614833	0.977633627	2.531414518	14.42906275	35.7	5.850658824
2016	29.44683156	29.4973489	1.419049482	3.009911429	17.15649515	34.25	4.456058824
2016	27.21810373	30.1748004	1.35454387	2.944277356	16.78238093	38.25	5.131823529
2016	37.45731053	31.92442752	1.88406344	2.845845789	16.221321	32.25	4.503670588
2016	36.13282702	35.34230055	2.154245354	2.807820113	16.00457465	35.6	4.725552941
2016	44.61864559	38.59865045	1.576458458	2.380400983	13.56828561	34.5	4.457047059
2016	43.00186957	38.28515629	1.183283834	2.879297077	16.41199334	39.1	3.126588235
2016	32.76814128	21.95555488	2.173649481	3.100911304	17.67519444	35.9	5.525647059
2016	47.73868581	32.1921093	1.583058203	2.882460373	16.43002413	38.75	4.537058824
2016	25.42953198	21.99858964	2.099162506	2.362815345	13.46804747	35.55	3.882282353
2016	35.7389715	26.67326725	1.103133256	3.40889998	19.43072988	38.75	3.765882353
2016	35.25043027	32.20726203	1.551187323	2.817926568	16.06218144	36.75	4.543764706
2016	50.61238598	32.32911745	2.132520464	3.367203995	19.19306277	37.3	4.598752941
2016	52.51324682	28.10972178	1.449269389	2.99697619	17.08276428	34.95	4.526976471
2016	29.96146643	33.44813595	1.7633564	3.03321019	17.28929808	34.2	6.262941176
2016	39.0844571	30.15614131	2.125349751	2.776558792	15.82638511	33	4.759070588
2016	28.59847185	27.87139354	1.480000841	2.654967475	15.13331461	38.45	5.312470588
2016	32.35028063	27.25830914	1.183727696	2.971265944	16.93621588	36.95	5.592141176
2016	39.80853527	38.61169664	1.072364115	3.026418394	17.25058484	33.05	5.130894118
2016	34.24516384	25.94704487	1.654444127	2.584723857	14.73292598	37.05	5.553741176
2016	29.57136987	32.56187837	1.580324564	2.952025037	16.82654271	38.05	5.596282353
2016	41.13977604	30.91830661	0.701254151			35.55	3.633882353
2016	27.41851399	24.39008622	0.988450989	2.374388608	13.53401506	30.35	5.692729412

2016	35.5843876	23.94763923	1.498252048	2.979494095	16.98311634		29.35	6.646211765
2016	36.02020252	33.27957439	1.824082563	3.323232972	18.94242794		26.95	4.686482353
2016	40.73385219	37.03202155	1.586220457	3.1157491	17.75976987		37.25	5.024282353
2016	49.88367442	35.61312211	1.183497483	2.75083408	15.67975425		36.4	6.579294118
2016	37.01781495	29.14642042	1.307346706	3.04199188	17.33935371		35.8	3.422035294
2016	31.9154797	27.66768849	1.379309673	2.998392613	17.09083789		28.65	4.977882353
2016	31.36239476	24.1723047	1.607936867	2.192292707	12.49606843		30	4.566058824
2016	36.6299242	29.40722445	1.387720558	2.945107337	16.78711182			4.472847059
2016	47.92950463	40.56266643	1.167156574	2.762498823	15.74624329		34.05	6.386352941
2016	49.439508	33.79124342	1.473626141	2.867161101	16.34281827		32.4	4.750552941
2016	33.79378185	32.98415227	1.646030745	2.799490286	15.95709463		30.6	4.219058824
2016	32.28423848	31.45216032	1.167846473	3.093066671	17.63048002		33.25	3.202823529
2016	40.46321127	31.92620017	1.458781241	2.740499045	15.62084456		33.75	3.157647059
2016	45.32815422	39.28951236	0.125444303	3.340580434	19.04130847		31.45	4.070588235
2016	41.5845889	29.41407706	1.109849552	2.675901153	15.25263657		31.35	4.100611765
2016	31.8224159	29.45724955	1.569185594	2.588972545	14.75714351		33.35	3.602823529
2016	41.34033593	27.45697706	1.327148991	2.781784904	15.85617395		30.05	
2016	39.63358868	30.20277181	1.672423266	2.842208527	16.2005886		31.7	3.970729412
2016	56.14271293	52.0534131	1.605621165	2.510845965	14.311822		31.15	5.216564706
2016	30.64988264	26.66475751	1.489929642	3.233680564	18.43197922		33.3	3.219764706
2016	39.20819095	35.43276661	1.040648572	2.886979008	16.45578035		33.6	3.897411765
2016	62.40365568	46.50522958	1.463074598				31.8	
2016	56.79298309	46.49289793	7.492072483	2.903098691	16.54766254	*		3.551647059
2016	33.82064464	26.57280962	1.583048317	1.369429995	7.805750972		34.3	4.760282353
2016	37.16804791	32.87688706	1.147861798				33.15	4.943152941
2016	40.27167925	37.15388245	1.691767275	2.59292099	14.77964964		32.3	4.893458824
2016	26.65822663	27.64458246	1.224205595	2.797659122	15.946657		35.45	7.727294118
2016	39.83814732	30.14071112	1.587938841	2.747868031	15.66284778		30.4	6.687352941
2016	32.53189958	32.3974581	1.06323191	2.646482428	15.08494984		34.1	4.2024
2016	26.11778271	30.98091637	1.460564414	3.722479934	21.21813563		34.5	5.151
2016								4.824470588
2016	51.77349946	58.94654478	1.746409105	2.980853719	16.9908662		34.65	5.238588235
2016	26.69461312	23.80536365	1.026939459	3.076030111	17.53337163		31.9	4.389647059

2016	33.79570013	31.40859587	1.075693516	2.233295389	12.72978372		31.05	4.224
2016	35.98375416	34.82446406	1.238781562	3.038359598	17.31864971		26.75	
2016	28.03644155	25.50719319	1.910948254	2.487347094	14.17787844		32.46	3.494105882
2016			1.792226906	2.978271567	16.97614793		33.8	4.110117647
2016	35.19933444	37.80246598	1.524548644	3.321661388	18.93346991		35.4	3.047176471
2016	29.86263106	25.73765891	1.88655315	2.743841928	15.63989899		32.35	6.176564706
2016	40.28270186	39.75824199	1.355718888	3.035614719	17.3030039		32.6	6.104
2016	46.10450417	40.71113672	1.477424158				31.35	5.439364706
2016	28.1856769	23.09130798	1.789093323	2.718769445	15.49698584		26.5	4.937317647
2016	44.80666579	44.02615657	1.791728064				27.55	4.596705882
2016	38.1012703	35.32574371	1.615175842	2.395404324	13.65380465		27.25	3.954823529
2016	29.35999139	30.0784392	1.956346526	2.730316021	15.56280132		28.65	5.267294118
2016	38.32367101	40.31344405	2.386927609	2.771523051	15.79768139		29.4	3.375058824
2016	39.0523715	42.6749844	1.970970036	2.869001776	16.35331012		30.6	4.125552941
2016	33.65572462	33.14250631	1.246688762	3.346466144	19.07485702		27.9	5.666964706
2016	37.9536776	34.13634102	1.906077899				26.55	3.219764706
2016	40.28955383	51.44976157	1.963240012	2.479820174	14.13497499		27.6	5.160247059
2016	41.79725304	40.8884143	1.746131097	3.004432015	17.12526248		31.9	4.455235294
2016	47.04534305	41.65483383	1.469313312	2.652919049	15.12163858		28.35	3.747764706
2016	34.8485362	40.60819821	2.193044985	3.105516108	17.70144181		30.4	4.312776471
2016	43.85828096	37.94393693	1.978258542	3.344297285	19.06249453		30.5	4.632847059
2016	40.25516773	37.31035961	1.171302966	2.550044664	14.53525458		30.8	4.192941176
2016	39.73677087	34.25410502	1.265448612	2.63567867	15.02336842		31.9	
2016	35.84733032	40.5264724	1.225202276	3.343129006	19.05583534		29.85	4.310188235
2016	16.06443898	20.92776669	1.625732624	2.440925655	13.91327624		33.6	6.472870588
2016	31.06936298	32.33151858	1.561924127	3.166936781	18.05153965		36.5	5.257670588
2016	49.17575588	46.18898737	1.728510797	2.895389554	16.50372046		31.1	4.684470588
2016	36.14737764	34.45353285	2.140480474	2.567706325	14.63592605		30.15	5.521623529
2016	39.98624066	45.47597494	2.420428548				26.35	4.462117647
2016	33.41217836	29.4588204	1.504189972	0.986464438	5.622847295		26.55	3.604294118
2016	39.76682486	35.07132779	1.541083128	2.46476051	14.04913491		30.9	3.064470588
2016	40.07066001	41.9002686	1.365507687	3.131760655	17.85103573		32.55	3.758117647
2016	43.03834203	43.8209394	1.303780754	3.492978786	19.90997908	*		4.885552941

2016	26.04412288	26.92300427	2.004857051	2.60905637	14.87162131	27.7	3.281882353
2016	50.4927182	55.33192079	1.365801256			28.6	3.302588235
2016	32.1223972	33.11841414	0.76150406	2.685327227	15.3063652	36.1	6.227858824
2016	39.45268764	37.39494806	2.002023201	2.672974754	15.2359561	34.6	5.313976471
2016	35.6672995	30.49800016	1.418017147	1.306639235	7.447843642	32.65	5.354541176
2016	28.42662005	33.41826337	1.76257059	2.840615689	16.19150943	29.2	3.943529412
2016	35.25264602	31.60999316	0.995267679	2.695648888	15.36519866	29.6	4.058741176
2016	37.75819702	42.59279193	1.073675098	3.428564828	19.54281952	28.1	5.709482353
2016	34.19621742	30.67429098	2.265960788			22.15	3.809882353
2016	31.69400783	32.00903055	1.991752844	3.010747897	17.16126301	29.55	3.033411765
2016	33.40608728	38.46591494	1.720328338	3.313986172	18.88972118	29.4	2.805647059
2016	35.02271774	41.83818859			13.54	37.5	9.060042743
2016	37.36054229	40.81657577			14.74	36.53	7.288893708
2016	35.80740097	40.161969			13.68	39.4	7.875146999
2016	35.47814015	36.21433524			14.06	39.8	8.613691395
2016	32.87316513	37.49094928			13.18	34.8	8.565225372
2016	31.38469641	35.44649657			12.67	31.93	6.846973007
2016	35.79515295	46.28177977			14.1	39.03	6.66247631
2016	38.20943476	40.69594841			13.45	38.2	8.764540773
2016	37.1321421	42.94856846			13.77	40.8	7.548919454
2016	39.33093284	40.4900671			15.82	35.97	7.28434522
2016	29.92773217	35.6317443			13.65	35.9	8.31718195
2016	35.69779563	42.95239276			13.88	40.2	7.79670183
2016	35.74561446	41.34579769			14.26	39.9	6.500746062
2016	31.53051548	43.31550461			13.14	31.97	6.368361771
2016	36.01147727	45.17715225			13.83	35.47	6.541988637
2016	33.33863595	40.53155184			13.62	37.2	8.022664662
2016	39.03876049	38.62939597			13.54	39.1	8.564533693
2016					*	*	
2016	35.45536428	41.10013896			14.01	35.9	8.231761625
2016	33.4968204	37.42067795			13.3	36.3	7.367529391
2016	29.68831896	40.7663928			13.53	34.13	6.903548846
2016	40.72055042	48.72064044			15.98	36.3	6.480027659

2016	36.61238177	42.78713909	14.46	34.23	6.874943781
2016	37.99946327	46.20667156	15.19	35.3	7.895173926
2016	34.21693287	45.66192313	15.95	34.17	6.009410699
2016	38.4314248	48.88872355	14.4	36.33	7.003072215
2016	29.32208567	42.55741605	14.05	36.27	6.146153358
2016	29.2166392	37.79653812	12.87	36.33	6.764092338
2016	33.95328547	38.48680456	15.21	41.63	7.427383937
2016	26.96802929	37.75459789	14.33	41.57	8.205537381
2016	27.21488292	36.06394985	14.03	36.57	7.367199603
2016	29.13575364	41.4195698	14.2	35	5.437559832
2016	32.26468249	43.05427802	13.78	37.07	7.77346367
2016	29.98437649	36.20526567	15.08	39.43	8.091807363
2016	29.79829451	39.73976609	13.27	40.57	6.756056016
2016	33.53845562	42.57174942	13.96	35.63	7.017709739
2016	38.24166449	45.29615723	15.06	39.23	7.172271942
2016			*	*	
2016	34.59807036	44.6781286	14.14	44.03	8.016823748
2016	29.26596751	41.09585192	13.67	36.53	8.141712808
2016	34.0266607	42.73345319	14.24	34.87	6.501687787
2016	36.98698566	43.44465556	13.92	35.03	8.674647925
2016	29.72649956	44.75385692	14.37	35.37	8.042511988
2016	32.54420211	39.97731598	12.75	33.83	7.2783115
2016	33.1238412	40.37729485	13.89	34.63	7.62237577
2016	30.14811229	39.68904884	13.1	36.3	9.47086879
2016	28.29162778	38.15207154	13.63	32.47	7.025962924
2016	31.8752742	40.43998007	13.98	37.37	8.286042567
2016	34.84748504	41.30269551	13.74	34.83	8.066121654
2016	33.49503999	44.23528777	13.17	34.27	5.628550022
2016	29.75859255	44.50977272	13.37	33.27	7.157680818
2016	29.40560996	38.84229299	12.97	36.73	8.736242639
2016	34.03020296	43.07421775	14.13	39.4	5.351901708
2016	32.28067421	41.48767631	14	39.4	6.609996607
2016	30.64649917	38.1186843	12.78	40.47	6.891941488

2016	27.23055775	36.02535467	13.78	37.37	5.51638307
2016	29.7369769	36.61658053	12.82	33.63	7.451894306
2016	31.82577132	43.38349559	12.78	36.93	7.104707562
2016	32.56974248	40.64731191	14.62	38.03	5.073166063
2016	33.0457791	42.90459519	13.69	41.47	7.264093118
2016	33.32655393	39.43778334	13.95	36.47	6.217306607
2016	27.99598746	42.57250649	13.44	42.77	6.388930411
2016	28.87694834	37.74778726	12.67	40.33	7.683034142
2016	30.90984251	43.2598512	15.03	37.3	4.974846882
2016	26.36278513	37.67745277	12.88	40.3	7.82432777
2016	32.82894662	40.4753773	13.39	39.17	7.072729328
2016	33.74955272	37.92926581	12.6	38.07	9.662184483
2016	28.96890153	31.79355857	13.63	34.1	8.666550102
2016	31.11780419	38.18355762	13.94	32.73	6.943896132
2016	31.74968792	34.04787132	12.7	36.93	8.35579034
2016			*	*	
2016	30.16664614	33.77267281	13.05	35.5	8.233589701
2016	33.19830846	37.00453853	13.3	32.97	7.323271085
2016	33.45786586	37.05111764	13.44	39.33	8.072829395
2016	29.0737715	36.6517297	12.43	36.37	8.662422879
2016	31.74065024	38.9324485	12.91	30.23	7.301793888
2016	24.96656765	33.85370506	12.2	34.67	7.46926947
2016	29.57538851	37.4825462	12.92	35.9	6.92862276
2016	31.83970666	39.66838608	13.38	30	6.366988136
2016	30.03964876	38.69920387	13.76	33.07	6.683183737
2016	28.4818762	34.35543172	12.8	37.2	6.458850048
2016	32.87695254	36.94499927	13.48	33.9	6.503381887
2016	31.60411158	37.42454495	13.96	29.77	6.961360002
2016	34.01108463	43.74174871	13.6	32.77	6.20032
2016	35.65191754	41.54293608	14.54	31.1	6.823142356
2016	34.93354959	42.80727497	13.81	35	7.624989043
2016	27.97177106	33.37934064	14.19	31.6	6.417801911
2016	30.52976738	37.21483473	12.66	33.67	6.653731405

2016	29.24944297	35.90485534	13.24	31.63	6.61966753
2016	29.14432288	34.50496015	13.19	32.93	6.086534829
2016	29.98954445	36.598972	12.63	31.93	6.371499696
2016	30.25395298	40.79583539	14.67	32.55	6.672778341
2016	31.17914603	39.72973343	12.95	38.93	7.557125365
2016	36.66999561	43.96515843	14.05	39.1	6.866050096
2016	31.96445585	37.73323246	13.59	36.93	7.30246873
2016	33.28754681	37.49063747	13.64	36.67	7.344059378
2016	29.7028711	41.78052272	14.38	41.93	6.465397771
2016	27.56801483	36.62337473	11.98	37.6	8.329549701
2016	37.72661127	46.3114961	15.16	36.37	7.110809533
2016	31.19784771	38.67181891	13.56	34.8	7.548165185
2016	30.09863736	38.14767598	13.24	37.63	8.049878032
2016	30.10589924	41.09172466	14.63	31.57	6.400728296
2016	31.29437353	36.33053591	13.09	38.23	6.544411993
2016	31.63024502	34.52775044	13.34	37.77	8.104744752
2016	29.25518603	38.67520373	13.2	39.47	7.99742946
2016	30.65550538	42.01647974	14.36	37.03	6.773039713
2016	29.20405145	39.21331098	14.26	41	6.78223715
2016			*	*	
2016	29.73986746	35.117723	13.64	39.53	8.222508718
2016	29.4320652	41.07534934	13.82	37.67	8.305168126
2016	31.81741447	38.81389809	15.18	33.27	4.886448974
2016	29.54047579	35.46086148	13.85	35.13	8.026398912
2016	26.92974261	38.57980077	13.11	36.33	8.191190652
2016	33.83285797	40.40113125	14.72	36.07	8.499522623
2016	26.29027932	39.5580532	13.72	36.57	6.163447965
2016	27.43943158	34.85025695	13.02	37.43	7.640802944
2016	33.04554561	40.77264867	14.49	34.47	8.949206246
2016			*	*	
2016	35.98218145	41.24676871	13.69	33.2	7.689240458
2016	24.61710306	34.51171693	13	36.13	7.105849825
2016	30.78338482	38.46358251	12.22	35.23	7.933515953

2016	36.35563415	39.64162851	14.09	37	8.548544463
2016	36.43932078	45.77355287	13.96	38.73	7.17479807
2016	32.66608071	44.11955305	14.03	36.83	8.543452561
2016	30.5153359	38.49805558	12.54	39.67	9.755958405
2016	35.00632774	49.47393896	14.75	38.23	7.264041862
2016	34.06417685	39.21886365	13.63	35.67	8.36936018
2016	33.72816425	38.8035449	13.05	36.37	8.642794879
2016	29.6738632	37.6580624	14	34.23	7.330808911
2016	33.52466023	44.58209524	14.51	43.93	9.217489735
2016	28.76339152	39.13345171	14.17	35.63	7.048455576
2016	33.26199361	36.92224526	14.43	38	7.054352461
2016	32.14939622	39.0150203	14.33	37.27	7.726763861
2016	34.03313161	40.78543888	14.95	37.97	8.042773485
2016	36.21920672	40.59764308	14.96	38.73	7.224034965
2016	32.56845557	42.38011565	13.63	40.63	6.36091614
2016	31.14050887	41.43029527	14.52	39.03	8.161459507
2016	24.91646311	33.49295183	14.24	36.77	7.060865033
2016			*	*	
2016	31.72304086	44.04411224	14.75	41	6.835816154
2016	28.67992311	40.07323942	14.75	38.77	6.60464681
2016	31.15667843	38.39312605	12.86	41.53	7.117674116
2016			*	*	
2016	31.6241296	44.40166329	14.55	39.03	5.554685818
2016	35.51307088	37.81050378	14.18	41.13	7.159739098
2016	33.08508787	40.49307642	13.88	37.73	5.255202215
2016	36.73522692	42.52322238	14	38.63	6.049049466
2016	35.47572888	42.31388646	14.44	35	5.450851464
2016	28.44083489	38.4529623	14.08	37.93	5.559294689
2016	38.95518901	42.64150988	15.02	37.87	5.209503092
2016	36.2699238	38.15685438	14.04	35.97	5.975512624
2016	36.44759395	38.5603707	14.65	38.9	5.916015339
2016	29.59054054	38.59047499	13.71	36.8	5.249364523
2016	34.85778342	43.40682914	14.84	35.53	5.296414996

2016	31.30677614	36.83163238		13.69		39.33	5.631953042
2016	31.31295508	41.06008888		13.99		39.47	7.266338833
2016	34.06063529	40.82285632		14.6		42.23	7.074713532
2016	28.49991066	40.86947871		13.54		40.9	8.015246748
2016	28.73383602	34.19690786		13.43		38.63	7.867572814
2016	35.07322587	40.61246464		13.55		40.97	7.600575143
2016	24.93232037	34.13238576		13.36		42.13	7.414847589
2016	30.8142738	42.4468721		14.56		38.4	6.062254996
2016			*		*		
2016	31.10005786	38.17379549		14.57		41.53	6.397028828
2016	32.21163249	39.66357184		15.01		41.87	7.63569328
2016	31.30599959	43.54251911		15.08		42.37	7.346868763
2016	31.68980944	40.58838887		15.62		42.5	6.829720792
2016	26.39880789	37.52492673		13.74		39.83	6.653978931
2016	30.15997695	34.54640965		13.92		41.83	8.301202996
2016	30.62336756	40.32693305		13.74		42	6.811484058
2016	30.97223385	38.9210047		13.92		40.93	7.693144544
2016	34.71696057	43.87210609		14.49		40.23	7.810616937
2016	31.89853862	39.6221448		12.91		40.17	8.357899831
2016	30.89404134	39.02008167		15.38		37.77	6.815313398
2016	33.71020652	43.66075323		14.04		46.67	7.398394143
2016	33.3488542	41.49867882		15.22		39	6.155154486
2016	38.56007234	38.88435143		13.05		39.13	8.218906813
2016	31.06634511	38.23391352		13.53		40.97	8.047429441
2016	30.22333496	37.74543221		13.77		40.5	6.94903123
2016	28.06259289	38.04811861		13.86		37.23	7.251554643
2016	30.63133094	36.9110656		13.67		40.5	8.740080545
2016	31.09931534	40.93554871		13.91		44.03	7.569382948
2016	27.71680015	39.25873944		13.28		45.03	7.263018276
2016	30.56208729	40.25550608		14.02		45.47	8.479993419
2016	30.41554093	44.54245596		13.36		45.17	6.428771331
2016	28.69973571	42.29352808		14.18		43.23	7.889662256
2016			*		*		

2016	32.4145576	41.07875398	13.45	47.7	7.615779248
2016	33.26548047	41.53252539	14.15	41.3	6.271252847
2016	25.31056442	35.13722403	13.42	40.9	8.956691701
2016	34.34141281	39.06660852	13.73	44.23	8.545706278
2016	29.61359726	39.76404003	13.88	38.33	7.401566685
2016	30.42176553	38.58654327	13.63	42.7	8.450778279
2016	28.74915355	32.82548149	12.77	42.6	9.054468301
2016	31.03221595	35.6291988	13.56	44.6	9.006783417
2016	30.02512006	39.4662116	13.41	45.4	7.008082968
2016	28.97155948	35.84282037	12.78	39.57	7.592378776
2016	25.78893808	33.52188219	12.8	36.27	8.019468371
2016	28.57204088	32.77220383	12.87	38.6	7.687013968
2016	29.76657171	34.7366795	13.8	43.13	8.543623829
2016	33.56883217	37.42563921	14.13	39.73	6.945403145
2016	31.04198552	36.99720884	13.93	41.13	7.327963113
2016	31.25586586	35.48360332	14.19	40.5	7.813664307
2016	28.54490696	36.74534271	13.87	38.6	7.562013081
2016	29.41735376	35.34023063	14	37.07	9.046627183
2016	30.0682273	38.9527237	13.59	42	8.384925922
2016	29.67287118	33.74728023	13.85	44.47	8.560000624
2016	30.71096688	40.60705972	13.58	32.97	6.799704076
2016	37.76825883	43.42710793	15.28	32.57	6.043382523
2016	36.61962768	46.6446573	14.62	33.97	6.632329028
2016	33.23143959	41.69932724	14.25	34.2	7.004437909
2016	37.27379464	45.75188864	15.05	34.9	6.499970042
2016	29.39728833	37.50728739	13.39	35.47	6.831022218
2016			*	*	
2016	33.71973575	37.65613718	14.31	34.07	6.620235276
2016	36.10527525	41.76371567	14.67	38.07	7.182899728
2016	33.32908106	45.02735253	13.86	34.73	8.094689101
2016	30.05027755	39.91914466	13.51	34.23	8.051814042
2016	37.31025333	48.7205541	14.59	37.07	7.029504854
2016	35.54315096	39.2548673	14.68	36.63	7.344896048

2016	31.0878941	35.77335186	13.01	39.27	7.921627452
2016	27.13521143	31.63769121	12.15	37.37	7.44697225
2016	40.59320467	52.34133807	15.89	31.8	6.960435512
2016	31.48482641	40.11991747	13.64	36.37	6.278866319
2016	36.28480735	42.19402267	14.96	35.83	6.998138742
2016	32.9777124	39.23144151	14	37.93	7.579222228
2016	30.95491724	38.43299896	13.78	37.9	6.485082487
2016	33.92077239	34.09134592	13	38.03	8.987455272
2016	31.31629786	42.11484987	13.16	38	7.081025712
2016	43.23485894	49.20197937	15.74	38.6	6.914393879
2016	33.24894367	39.9169577	14.25	34.3	7.026049374
2016	33.78496621	40.31175679	13.62	36.5	6.227460983
2016			*	*	
2016	33.37804164	42.6722829	12.72	29.13	6.113295706
2016	30.38954522	40.9249143	13.33	33.97	6.935435703
2016	39.08276465	50.25855831	14.67	29	6.812563909
2016	33.29214449	42.6518418	14.53	31.83	6.965609606
2016	38.44768883	47.37695135	13.96	32	6.214098753
2016	35.20779086	43.77704613	14.97	33.4	8.160384611
2016	32.56841276	42.32973694	12.91	28.97	6.919285971
2016	33.76852766	43.47267206	13.27	37.8	7.16180976
2016	39.12333258	48.16066518	14.14	28.85	5.383262224
2016	34.93067066	42.31260914	14.21	36.1	8.041581981
2016	32.17958346	40.23976265	14.56	32.97	9.410861317
2016	34.52005291	39.56874733	14.21	28.4	5.719328515
