# Supplementary material for: Novel sources of variation in grain Zinc (Zn) concentration in bread wheat germplasm derived from Watkins landraces
Source: PLoS One. 2020 Feb 28;15(2):e0229107. doi: 10.1371/journal.pone.0229107 (PMC7048275; doi:10.1371/journal.pone.0229107)
Supplement: S3 Table — (PDF) [file pone.0229107.s003.pdf]

Table S3. Primary data of Zn and Fe concentrations (mg/kg) in wholegrain and white flour of 24 wheat lines grown at Nottingham and Rothamsted sites over

Sample ID!	Sr No!	Genotype!	Plot No!	Sample_grain wt_g	Sample Vol_ml	Replication!	Levels!	H_Zn-H_Fe!	H_Zn-L_Fe!
2	144	PxW566 - 20	12	0.306	15	R3	L_Zn-L_Fe		
3	141	PxW291 - 23	13	0.321	15	R3	L_Zn-L_Fe		
5	127	PxW7 - 60	34	0.301	15	R1	L_Zn-L_Fe		
6	136	PxW291 - 75	64	0.312	15	R2	L_Zn-L_Fe		
8	69	PxW291 - 23	102	0.321	15	R3	L_Zn-L_Fe		
11	131	PxW299 - 87	124	0.309	15	R1	L_Zn-L_Fe		
16	132	PxW566 - 20	212	0.308	15	R1	L_Zn-L_Fe		
18	67	PxW7 - 60	225	0.298	15	R3	L_Zn-L_Fe		
19	134	PxW273 - 21	227	0.308	15	R2	L_Zn-L_Fe		
31	140	PxW273 - 21	326	0.328	15	R3	L_Zn-L_Fe		
32	70	PxW291 - 75	356	0.301	15	R3	L_Zn-L_Fe		
37	62	PxW273 - 21	391	0.307	15	R2	L_Zn-L_Fe		
40	60	PxW566 - 20	412	0.305	15	R1	L_Zn-L_Fe		
50	143	PxW299 - 87	429	0.317	15	R3	L_Zn-L_Fe		
53	135	PxW291 - 23	441	0.31	15	R2	L_Zn-L_Fe		
61	56	PxW273 - 21	553	0.312	15	R1	L_Zn-L_Fe		
64	133	PxW7 - 60	564	0.313	15	R2	L_Zn-L_Fe		
65	137	PxW299 - 87	574	0.313	15	R2	L_Zn-L_Fe		
68	68	PxW273 - 21	603	0.307	15	R3	L_Zn-L_Fe		
70	129	PxW291 - 23	621	0.312	15	R1	L_Zn-L_Fe		
72	61	PxW7 - 60	633	0.305	15	R2	L_Zn-L_Fe		
73	128	PxW273 - 21	643	0.304	15	R1	L_Zn-L_Fe		
77	142	PxW291 - 75	661	0.317	15	R3	L_Zn-L_Fe		
78	59	PxW299 - 87	664	0.309	15	R1	L_Zn-L_Fe		
81	66	PxW566 - 20	681	0.305	15	R2	L_Zn-L_Fe		
83	63	PxW291 - 23	695	0.304	15	R2	L_Zn-L_Fe		
85	130	PxW291 - 75	714	0.312	15	R1	L_Zn-L_Fe		
86	71	PxW299 - 87	715	0.302	15	R3	L_Zn-L_Fe		
113	138	PxW566 - 20	826	0.318	15	R2	L_Zn-L_Fe		
114	72	PxW566 - 20	827	0.316	15	R3	L_Zn-L_Fe		
123	55	PxW7 - 60	915	0.319	15	R1	L_Zn-L_Fe		

124	139	PxW7 - 60	916	0.327	15	R3	L_Zn-L_Fe
130	58	PxW291 - 75	1004	0.316	15	R1	L_Zn-L_Fe
134	65	PxW299 - 87	1051	0.317	15	R2	L_Zn-L_Fe
135	64	PxW291 - 75	1058	0.317	15	R2	L_Zn-L_Fe
136	57	PxW291 - 23	1136	0.31	15	R1	L_Zn-L_Fe
146	144	PxW566 - 20	12	0.3	15	R3	L_Zn-L_Fe
147	141	PxW291 - 23	13	0.304	15	R3	L_Zn-L_Fe
149	127	PxW7 - 60	34	0.3	15	R1	L_Zn-L_Fe
150	136	PxW291 - 75	64	0.3	15	R2	L_Zn-L_Fe
152	69	PxW291 - 23	102	0.31	15	R3	L_Zn-L_Fe
155	131	PxW299 - 87	124	0.297	15	R1	L_Zn-L_Fe
160	132	PxW566 - 20	212	0.318	15	R1	L_Zn-L_Fe
162	67	PxW7 - 60	225	0.307	15	R3	L_Zn-L_Fe
163	134	PxW273 - 21	227	0.302	15	R2	L_Zn-L_Fe
175	140	PxW273 - 21	326	0.3	15	R3	L_Zn-L_Fe
176	70	PxW291 - 75	356	0.3	15	R3	L_Zn-L_Fe
181	62	PxW273 - 21	391	0.305	15	R2	L_Zn-L_Fe
184	60	PxW566 - 20	412	0.309	15	R1	L_Zn-L_Fe
194	143	PxW299 - 87	429	0.305	15	R3	L_Zn-L_Fe
197	135	PxW291 - 23	441	0.304	15	R2	L_Zn-L_Fe
205	56	PxW273 - 21	553	0.302	15	R1	L_Zn-L_Fe
208	133	PxW7 - 60	564	0.303	15	R2	L_Zn-L_Fe
209	137	PxW299 - 87	574	0.317	15	R2	L_Zn-L_Fe
212	68	PxW273 - 21	603	0.306	15	R3	L_Zn-L_Fe
214	129	PxW291 - 23	621	0.3	15	R1	L_Zn-L_Fe
216	61	PxW7 - 60	633	0.303	15	R2	L_Zn-L_Fe
217	128	PxW273 - 21	643	0.31	15	R1	L_Zn-L_Fe
221	142	PxW291 - 75	661	0.311	15	R3	L_Zn-L_Fe
222	59	PxW299 - 87	664	0.303	15	R1	L_Zn-L_Fe
225	66	PxW566 - 20	681	0.302	15	R2	L_Zn-L_Fe
227	63	PxW291 - 23	695	0.301	15	R2	L_Zn-L_Fe
229	130	PxW291 - 75	714	0.302	15	R1	L_Zn-L_Fe
230	71	PxW299 - 87	715	0.304	15	R3	L_Zn-L_Fe

257	138	PxW566 - 20	826	0.307	15	R2	L_Zn-L_Fe
258	72	PxW566 - 20	827	0.305	15	R3	L_Zn-L_Fe
267	55	PxW7 - 60	915	0.306	15	R1	L_Zn-L_Fe
268	139	PxW7 - 60	916	0.301	15	R3	L_Zn-L_Fe
274	58	PxW291 - 75	1004	0.303	15	R1	L_Zn-L_Fe
278	65	PxW299 - 87	1051	0.288	15	R2	L_Zn-L_Fe
279	64	PxW291 - 75	1058	0.303	15	R2	L_Zn-L_Fe
280	57	PxW291 - 23	1136	0.306	15	R1	L_Zn-L_Fe
5	52	PxW299 - 87	121	0.313	24	R1	L_Zn-L_Fe
8	46	PxW291 - 23	202	0.3	24	R1	L_Zn-L_Fe
13	40	PxW7 - 60	275	0.305	24	R1	L_Zn-L_Fe
15	43	PxW273 - 21	332	0.31	24	R1	L_Zn-L_Fe
16	49	PxW291 - 75	334	0.312	24	R1	L_Zn-L_Fe
17	55	PxW566 - 20	365	0.31	24	R1	L_Zn-L_Fe
24	53	PxW299 - 87	857	0.303	24	R2	L_Zn-L_Fe
28	47	PxW291 - 23	956	0.305	24	R2	L_Zn-L_Fe
29	41	PxW7 - 60	957	0.311	24	R2	L_Zn-L_Fe
33	50	PxW291 - 75	1033	0.321	24	R2	L_Zn-L_Fe
35	44	PxW273 - 21	1116	0.309	24	R2	L_Zn-L_Fe
38	56	PxW566 - 20	1191	0.313	24	R2	L_Zn-L_Fe
39	48	PxW291 - 23	1206	0.307	24	R3	L_Zn-L_Fe
43	42	PxW7 - 60	1397	0.311	24	R3	L_Zn-L_Fe
44	57	PxW566 - 20	1407	0.315	24	R3	L_Zn-L_Fe
52	51	PxW291 - 75	1529	0.321	24	R3	L_Zn-L_Fe
53	54	PxW299 - 87	1546	0.308	24	R3	L_Zn-L_Fe
56	45	PxW273 - 21	1550	0.31	24	R3	L_Zn-L_Fe
59	109	PxW299 - 87	30	0.309	24	R1	L_Zn-L_Fe
60	103	PxW291 - 23	37	0.301	24	R1	L_Zn-L_Fe
65	112	PxW566 - 20	191	0.302	24	R1	L_Zn-L_Fe
67	100	PxW273 - 21	228	0.307	24	R1	L_Zn-L_Fe
69	106	PxW291 - 75	292	0.311	24	R1	L_Zn-L_Fe
73	97	PxW7 - 60	368	0.317	24	R1	L_Zn-L_Fe
80	113	PxW566 - 20	546	0.301	24	R2	L_Zn-L_Fe

85	110	PxW299 - 87	731	0.304	24	R2	L_Zn-L_Fe
90	101	PxW273 - 21	769	0.309	24	R2	L_Zn-L_Fe
91	107	PxW291 - 75	770	0.302	24	R2	L_Zn-L_Fe
92	104	PxW291 - 23	798	0.309	24	R2	L_Zn-L_Fe
94	98	PxW7 - 60	805	0.309	24	R2	L_Zn-L_Fe
95	114	PxW566 - 20	871	0.321	24	R3	L_Zn-L_Fe
96	111	PxW299 - 87	889	0.303	24	R3	L_Zn-L_Fe
99	99	PxW7 - 60	920	0.314	24	R3	L_Zn-L_Fe
102	105	PxW291 - 23	968	0.304	24	R3	L_Zn-L_Fe
105	108	PxW291 - 75	1052	0.302	24	R3	L_Zn-L_Fe
110	102	PxW273 - 21	1099	0.311	24	R3	L_Zn-L_Fe
119	52	PxW299 - 87	121	0.313	24	R1	L_Zn-L_Fe
122	46	PxW291 - 23	202	0.313	24	R1	L_Zn-L_Fe
127	40	PxW7 - 60	275	0.311	24	R1	L_Zn-L_Fe
129	43	PxW273 - 21	332	0.318	24	R1	L_Zn-L_Fe
130	49	PxW291 - 75	334	0.318	24	R1	L_Zn-L_Fe
131	55	PxW566 - 20	365	0.31	24	R1	L_Zn-L_Fe
138	53	PxW299 - 87	857	0.303	24	R2	L_Zn-L_Fe
142	47	PxW291 - 23	956	0.312	24	R2	L_Zn-L_Fe
143	41	PxW7 - 60	957	0.31	24	R2	L_Zn-L_Fe
147	50	PxW291 - 75	1033	0.301	24	R2	L_Zn-L_Fe
149	44	PxW273 - 21	1116	0.312	24	R2	L_Zn-L_Fe
152	56	PxW566 - 20	1191	0.311	24	R2	L_Zn-L_Fe
153	48	PxW291 - 23	1206	0.315	24	R3	L_Zn-L_Fe
157	42	PxW7 - 60	1397	0.314	24	R3	L_Zn-L_Fe
158	57	PxW566 - 20	1407	0.311	24	R3	L_Zn-L_Fe
166	51	PxW291 - 75	1529	0.31	24	R3	L_Zn-L_Fe
167	54	PxW299 - 87	1546	0.3	24	R3	L_Zn-L_Fe
170	45	PxW273 - 21	1550	0.315	24	R3	L_Zn-L_Fe
173	109	PxW299 - 87	30	0.32	24	R1	L_Zn-L_Fe
174	103	PxW291 - 23	37	0.307	24	R1	L_Zn-L_Fe
179	112	PxW566 - 20	191	0.316	24	R1	L_Zn-L_Fe
181	100	PxW273 - 21	228	0.314	24	R1	L_Zn-L_Fe

183	106	PxW291 - 75	292	0.304	24	R1	L_Zn-L_Fe
187	97	PxW7 - 60	368	0.313	24	R1	L_Zn-L_Fe
194	113	PxW566 - 20	546	0.306	24	R2	L_Zn-L_Fe
199	110	PxW299 - 87	731	0.301	24	R2	L_Zn-L_Fe
204	101	PxW273 - 21	769	0.305	24	R2	L_Zn-L_Fe
205	107	PxW291 - 75	770	0.314	24	R2	L_Zn-L_Fe
206	104	PxW291 - 23	798	0.305	24	R2	L_Zn-L_Fe
208	98	PxW7 - 60	805	0.307	24	R2	L_Zn-L_Fe
209	114	PxW566 - 20	871	0.309	24	R3	L_Zn-L_Fe
210	111	PxW299 - 87	889	0.302	24	R3	L_Zn-L_Fe
213	99	PxW7 - 60	920	0.306	24	R3	L_Zn-L_Fe
216	105	PxW291 - 23	968	0.302	24	R3	L_Zn-L_Fe
219	108	PxW291 - 75	1052	0.303	24	R3	L_Zn-L_Fe
224	102	PxW273 - 21	1099	0.309	24	R3	L_Zn-L_Fe
9	50	PxW264 - 50	118	0.322	15	R3	L_Zn-H_Fe
13	54	PxW811 - 10	130	0.302	15	R3	L_Zn-H_Fe
27	40	PxW398 - 18	319	0.313	15	R1	L_Zn-H_Fe
33	110	PxW264 - 50	368	0.307	15	R1	L_Zn-H_Fe
36	46	PxW398 - 18	388	0.305	15	R2	L_Zn-H_Fe
39	37	PxW223 - 80	411	0.311	15	R1	L_Zn-H_Fe
52	39	PxW291 - 39	439	0.31	15	R1	L_Zn-H_Fe
58	41	PxW546 - 25	524	0.307	15	R1	L_Zn-H_Fe
63	43	PxW223 - 80	562	0.315	15	R2	L_Zn-H_Fe
67	52	PxW398 - 18	594	0.302	15	R3	L_Zn-H_Fe
71	45	PxW291 - 39	629	0.312	15	R2	L_Zn-H_Fe
76	38	PxW264 - 50	656	0.308	15	R1	L_Zn-H_Fe
89	49	PxW223 - 80	727	0.313	15	R3	L_Zn-H_Fe
99	48	PxW811 - 10	751	0.312	15	R2	L_Zn-H_Fe
103	122	PxW264 - 50	767	0.3	15	R3	L_Zn-H_Fe
104	42	PxW811 - 10	779	0.31	15	R1	L_Zn-H_Fe
109	116	PxW264 - 50	817	0.3	15	R2	L_Zn-H_Fe
112	51	PxW291 - 39	824	0.309	15	R3	L_Zn-H_Fe
119	53	PxW546 - 25	852	0.301	15	R3	L_Zn-H_Fe

125	47	PxW546 - 25	931	0.312	15	R2	L_Zn-H_Fe
133	44	PxW264 - 50	1047	0.313	15	R2	L_Zn-H_Fe
153	50	PxW264 - 50	118	0.306	15	R3	L_Zn-H_Fe
157	54	PxW811 - 10	130	0.309	15	R3	L_Zn-H_Fe
171	40	PxW398 - 18	319	0.309	15	R1	L_Zn-H_Fe
177	110	PxW264 - 50	368	0.302	15	R1	L_Zn-H_Fe
180	46	PxW398 - 18	388	0.301	15	R2	L_Zn-H_Fe
183	37	PxW223 - 80	411	0.306	15	R1	L_Zn-H_Fe
196	39	PxW291 - 39	439	0.302	15	R1	L_Zn-H_Fe
202	41	PxW546 - 25	524	0.302	15	R1	L_Zn-H_Fe
207	43	PxW223 - 80	562	0.301	15	R2	L_Zn-H_Fe
211	52	PxW398 - 18	594	0.302	15	R3	L_Zn-H_Fe
215	45	PxW291 - 39	629	0.304	15	R2	L_Zn-H_Fe
220	38	PxW264 - 50	656	0.279	15	R1	L_Zn-H_Fe
233	49	PxW223 - 80	727	0.3	15	R3	L_Zn-H_Fe
243	48	PxW811 - 10	751	0.299	15	R2	L_Zn-H_Fe
247	122	PxW264 - 50	767	0.3	15	R3	L_Zn-H_Fe
248	42	PxW811 - 10	779	0.292	15	R1	L_Zn-H_Fe
253	116	PxW264 - 50	817	0.3	15	R2	L_Zn-H_Fe
256	51	PxW291 - 39	824	0.304	15	R3	L_Zn-H_Fe
263	53	PxW546 - 25	852	0.311	15	R3	L_Zn-H_Fe
269	47	PxW546 - 25	931	0.307	15	R2	L_Zn-H_Fe
277	44	PxW264 - 50	1047	0.31	15	R2	L_Zn-H_Fe
14	37	PxW264 - 50	298	0.302	24	R1	L_Zn-H_Fe
36	38	PxW264 - 50	1148	0.32	24	R2	L_Zn-H_Fe
55	39	PxW264 - 50	1549	0.313	24	R3	L_Zn-H_Fe
63	94	PxW264 - 50	150	0.309	24	R1	L_Zn-H_Fe
82	95	PxW264 - 50	670	0.301	24	R2	L_Zn-H_Fe
112	96	PxW264 - 50	1150	0.312	24	R3	L_Zn-H_Fe
128	37	PxW264 - 50	298	0.315	24	R1	L_Zn-H_Fe
150	38	PxW264 - 50	1148	0.304	24	R2	L_Zn-H_Fe
169	39	PxW264 - 50	1549	0.304	24	R3	L_Zn-H_Fe
177	94	PxW264 - 50	150	0.309	24	R1	L_Zn-H_Fe

196	95	PxW264 - 50	670	0.307	24	R2	L_Zn-H_Fe	
226	96	PxW264 - 50	1150	0.308	24	R3	L_Zn-H_Fe	
1	105	PxW273 - 71	4	0.307	15	R3	H_Zn-L_Fe	H_Zn-L_Fe
4	31	PxW254 - 2	18	0.317	15	R3	H_Zn-L_Fe	H_Zn-L_Fe
7	108	PxW546 - 24	97	0.31	15	R3	H_Zn-L_Fe	H_Zn-L_Fe
20	34	PxW396 - 56	229	0.311	15	R3	H_Zn-L_Fe	H_Zn-L_Fe
24	101	PxW546 - 20	269	0.301	15	R2	H_Zn-L_Fe	H_Zn-L_Fe
26	23	PxW546 - 20	313	0.304	15	R1	H_Zn-L_Fe	H_Zn-L_Fe
29	24	PxW546 - 24	320	0.318	15	R1	H_Zn-L_Fe	H_Zn-L_Fe
30	30	PxW546 - 24	326	0.299	15	R2	H_Zn-L_Fe	H_Zn-L_Fe
34	93	PxW273 - 71	374	0.321	15	R1	H_Zn-L_Fe	H_Zn-L_Fe
42	107	PxW546 - 20	423	0.301	15	R3	H_Zn-L_Fe	H_Zn-L_Fe
51	104	PxW264 - 17	430	0.301	15	R3	H_Zn-L_Fe	H_Zn-L_Fe
54	32	PxW264 - 17	463	0.318	15	R3	H_Zn-L_Fe	H_Zn-L_Fe
55	99	PxW273 - 71	488	0.313	15	R2	H_Zn-L_Fe	H_Zn-L_Fe
56	103	PxW254 - 2	508	0.31	15	R3	H_Zn-L_Fe	H_Zn-L_Fe
57	26	PxW264 - 17	518	0.318	15	R2	H_Zn-L_Fe	H_Zn-L_Fe
69	36	PxW546 - 24	618	0.315	15	R3	H_Zn-L_Fe	H_Zn-L_Fe
74	21	PxW273 - 71	645	0.322	15	R1	H_Zn-L_Fe	H_Zn-L_Fe
79	19	PxW254 - 2	667	0.307	15	R1	H_Zn-L_Fe	H_Zn-L_Fe
84	96	PxW546 - 24	703	0.317	15	R1	H_Zn-L_Fe	H_Zn-L_Fe
87	92	PxW264 - 17	715	0.309	15	R1	H_Zn-L_Fe	H_Zn-L_Fe
88	35	PxW546 - 20	720	0.31	15	R3	H_Zn-L_Fe	H_Zn-L_Fe
92	97	PxW254 - 2	742	0.318	15	R2	H_Zn-L_Fe	H_Zn-L_Fe
93	29	PxW546 - 20	743	0.315	15	R2	H_Zn-L_Fe	H_Zn-L_Fe
97	25	PxW254 - 2	747	0.322	15	R2	H_Zn-L_Fe	H_Zn-L_Fe
105	22	PxW396 - 56	789	0.314	15	R1	H_Zn-L_Fe	H_Zn-L_Fe
107	28	PxW396 - 56	812	0.313	15	R2	H_Zn-L_Fe	H_Zn-L_Fe
108	100	PxW396 - 56	816	0.302	15	R2	H_Zn-L_Fe	H_Zn-L_Fe
111	102	PxW546 - 24	823	0.312	15	R2	H_Zn-L_Fe	H_Zn-L_Fe
115	33	PxW273 - 71	839	0.313	15	R3	H_Zn-L_Fe	H_Zn-L_Fe
118	106	PxW396 - 56	850	0.301	15	R3	H_Zn-L_Fe	H_Zn-L_Fe
121	91	PxW254 - 2	870	0.315	15	R1	H_Zn-L_Fe	H_Zn-L_Fe

122	95	PxW546 - 20	874	0.306	15	R1	H_Zn-L_Fe	H_Zn-L_Fe
128	98	PxW264 - 17	949	0.309	15	R2	H_Zn-L_Fe	H_Zn-L_Fe
129	94	PxW396 - 56	956	0.325	15	R1	H_Zn-L_Fe	H_Zn-L_Fe
131	20	PxW264 - 17	1007	0.307	15	R1	H_Zn-L_Fe	H_Zn-L_Fe
138	27	PxW273 - 71	1164	0.327	15	R2	H_Zn-L_Fe	H_Zn-L_Fe
145	105	PxW273 - 71	4	0.305	15	R3	H_Zn-L_Fe	H_Zn-L_Fe
148	31	PxW254 - 2	18	0.303	15	R3	H_Zn-L_Fe	H_Zn-L_Fe
151	108	PxW546 - 24	97	0.303	15	R3	H_Zn-L_Fe	H_Zn-L_Fe
164	34	PxW396 - 56	229	0.31	15	R3	H_Zn-L_Fe	H_Zn-L_Fe
168	101	PxW546 - 20	269	0.305	15	R2	H_Zn-L_Fe	H_Zn-L_Fe
170	23	PxW546 - 20	313	0.305	15	R1	H_Zn-L_Fe	H_Zn-L_Fe
173	24	PxW546 - 24	320	0.304	15	R1	H_Zn-L_Fe	H_Zn-L_Fe
174	30	PxW546 - 24	326	0.302	15	R2	H_Zn-L_Fe	H_Zn-L_Fe
178	93	PxW273 - 71	374	0.307	15	R1	H_Zn-L_Fe	H_Zn-L_Fe
186	107	PxW546 - 20	423	0.305	15	R3	H_Zn-L_Fe	H_Zn-L_Fe
195	104	PxW264 - 17	430	0.309	15	R3	H_Zn-L_Fe	H_Zn-L_Fe
198	32	PxW264 - 17	463	0.302	15	R3	H_Zn-L_Fe	H_Zn-L_Fe
199	99	PxW273 - 71	488	0.303	15	R2	H_Zn-L_Fe	H_Zn-L_Fe
200	103	PxW254 - 2	508	0.304	15	R3	H_Zn-L_Fe	H_Zn-L_Fe
201	26	PxW264 - 17	518	0.301	15	R2	H_Zn-L_Fe	H_Zn-L_Fe
213	36	PxW546 - 24	618	0.308	15	R3	H_Zn-L_Fe	H_Zn-L_Fe
218	21	PxW273 - 71	645	0.303	15	R1	H_Zn-L_Fe	H_Zn-L_Fe
223	19	PxW254 - 2	667	0.302	15	R1	H_Zn-L_Fe	H_Zn-L_Fe
228	96	PxW546 - 24	703	0.302	15	R1	H_Zn-L_Fe	H_Zn-L_Fe
231	92	PxW264 - 17	715	0.302	15	R1	H_Zn-L_Fe	H_Zn-L_Fe
232	35	PxW546 - 20	720	0.311	15	R3	H_Zn-L_Fe	H_Zn-L_Fe
236	97	PxW254 - 2	742	0.303	15	R2	H_Zn-L_Fe	H_Zn-L_Fe
237	29	PxW546 - 20	743	0.305	15	R2	H_Zn-L_Fe	H_Zn-L_Fe
241	25	PxW254 - 2	747	0.303	15	R2	H_Zn-L_Fe	H_Zn-L_Fe
249	22	PxW396 - 56	789	0.309	15	R1	H_Zn-L_Fe	H_Zn-L_Fe
251	28	PxW396 - 56	812	0.3	15	R2	H_Zn-L_Fe	H_Zn-L_Fe
252	100	PxW396 - 56	816	0.312	15	R2	H_Zn-L_Fe	H_Zn-L_Fe
255	102	PxW546 - 24	823	0.311	15	R2	H_Zn-L_Fe	H_Zn-L_Fe

259	33	PxW273 - 71	839	0.305	15	R3	H_Zn-L_Fe	H_Zn-L_Fe
262	106	PxW396 - 56	850	0.308	15	R3	H_Zn-L_Fe	H_Zn-L_Fe
265	91	PxW254 - 2	870	0.305	15	R1	H_Zn-L_Fe	H_Zn-L_Fe
266	95	PxW546 - 20	874	0.303	15	R1	H_Zn-L_Fe	H_Zn-L_Fe
272	98	PxW264 - 17	949	0.299	15	R2	H_Zn-L_Fe	H_Zn-L_Fe
273	94	PxW396 - 56	956	0.306	15	R1	H_Zn-L_Fe	H_Zn-L_Fe
275	20	PxW264 - 17	1007	0.3	15	R1	H_Zn-L_Fe	H_Zn-L_Fe
282	27	PxW273 - 71	1164	0.302	15	R2	H_Zn-L_Fe	H_Zn-L_Fe
1	25	PxW273 - 71	36	0.313	24	R1	H_Zn-L_Fe	H_Zn-L_Fe
2	22	PxW264 - 17	48	0.332	24	R1	H_Zn-L_Fe	H_Zn-L_Fe
6	31	PxW546 - 20	165	0.306	24	R1	H_Zn-L_Fe	H_Zn-L_Fe
9	28	PxW396 - 56	213	0.315	24	R1	H_Zn-L_Fe	H_Zn-L_Fe
11	19	PxW254 - 2	265	0.302	24	R1	H_Zn-L_Fe	H_Zn-L_Fe
12	34	PxW546 - 24	274	0.31	24	R1	H_Zn-L_Fe	H_Zn-L_Fe
20	20	PxW254 - 2	810	0.31	24	R2	H_Zn-L_Fe	H_Zn-L_Fe
21	29	PxW396 - 56	825	0.314	24	R2	H_Zn-L_Fe	H_Zn-L_Fe
25	23	PxW264 - 17	876	0.32	24	R2	H_Zn-L_Fe	H_Zn-L_Fe
26	26	PxW273 - 71	916	0.302	24	R2	H_Zn-L_Fe	H_Zn-L_Fe
31	32	PxW546 - 20	1007	0.312	24	R2	H_Zn-L_Fe	H_Zn-L_Fe
32	35	PxW546 - 24	1023	0.319	24	R2	H_Zn-L_Fe	H_Zn-L_Fe
40	21	PxW254 - 2	1254	0.309	24	R3	H_Zn-L_Fe	H_Zn-L_Fe
42	30	PxW396 - 56	1317	0.326	24	R3	H_Zn-L_Fe	H_Zn-L_Fe
45	24	PxW264 - 17	1452	0.316	24	R3	H_Zn-L_Fe	H_Zn-L_Fe
46	36	PxW546 - 24	1483	0.309	24	R3	H_Zn-L_Fe	H_Zn-L_Fe
48	33	PxW546 - 20	1486	0.32	24	R3	H_Zn-L_Fe	H_Zn-L_Fe
57	27	PxW273 - 71	1587	0.315	24	R3	H_Zn-L_Fe	H_Zn-L_Fe
58	82	PxW273 - 71	23	0.327	24	R1	H_Zn-L_Fe	H_Zn-L_Fe
62	76	PxW254 - 2	94	0.302	24	R1	H_Zn-L_Fe	H_Zn-L_Fe
64	88	PxW546 - 20	173	0.336	24	R1	H_Zn-L_Fe	H_Zn-L_Fe
70	91	PxW546 - 24	313	0.324	24	R1	H_Zn-L_Fe	H_Zn-L_Fe
71	79	PxW264 - 17	328	0.309	24	R1	H_Zn-L_Fe	H_Zn-L_Fe
75	85	PxW396 - 56	393	0.317	24	R1	H_Zn-L_Fe	H_Zn-L_Fe
78	92	PxW546 - 24	456	0.305	24	R2	H_Zn-L_Fe	H_Zn-L_Fe

79	80	PxW264 - 17	458	0.321	24	R2	H_Zn-L_Fe	H_Zn-L_Fe
83	77	PxW254 - 2	673	0.314	24	R2	H_Zn-L_Fe	H_Zn-L_Fe
86	89	PxW546 - 20	732	0.312	24	R2	H_Zn-L_Fe	H_Zn-L_Fe
88	86	PxW396 - 56	746	0.314	24	R2	H_Zn-L_Fe	H_Zn-L_Fe
93	83	PxW273 - 71	804	0.312	24	R2	H_Zn-L_Fe	H_Zn-L_Fe
97	84	PxW273 - 71	890	0.322	24	R3	H_Zn-L_Fe	H_Zn-L_Fe
98	81	PxW264 - 17	910	0.309	24	R3	H_Zn-L_Fe	H_Zn-L_Fe
100	90	PxW546 - 20	925	0.304	24	R3	H_Zn-L_Fe	H_Zn-L_Fe
101	93	PxW546 - 24	951	0.317	24	R3	H_Zn-L_Fe	H_Zn-L_Fe
104	87	PxW396 - 56	992	0.317	24	R3	H_Zn-L_Fe	H_Zn-L_Fe
109	78	PxW254 - 2	1072	0.303	24	R3	H_Zn-L_Fe	H_Zn-L_Fe
115	25	PxW273 - 71	36	0.309	24	R1	H_Zn-L_Fe	H_Zn-L_Fe
116	22	PxW264 - 17	48	0.312	24	R1	H_Zn-L_Fe	H_Zn-L_Fe
120	31	PxW546 - 20	165	0.314	24	R1	H_Zn-L_Fe	H_Zn-L_Fe
123	28	PxW396 - 56	213	0.31	24	R1	H_Zn-L_Fe	H_Zn-L_Fe
125	19	PxW254 - 2	265	0.305	24	R1	H_Zn-L_Fe	H_Zn-L_Fe
126	34	PxW546 - 24	274	0.309	24	R1	H_Zn-L_Fe	H_Zn-L_Fe
134	20	PxW254 - 2	810	0.313	24	R2	H_Zn-L_Fe	H_Zn-L_Fe
135	29	PxW396 - 56	825	0.313	24	R2	H_Zn-L_Fe	H_Zn-L_Fe
139	23	PxW264 - 17	876	0.301	24	R2	H_Zn-L_Fe	H_Zn-L_Fe
140	26	PxW273 - 71	916	0.312	24	R2	H_Zn-L_Fe	H_Zn-L_Fe
145	32	PxW546 - 20	1007	0.313	24	R2	H_Zn-L_Fe	H_Zn-L_Fe
146	35	PxW546 - 24	1023	0.307	24	R2	H_Zn-L_Fe	H_Zn-L_Fe
154	21	PxW254 - 2	1254	0.306	24	R3	H_Zn-L_Fe	H_Zn-L_Fe
156	30	PxW396 - 56	1317	0.314	24	R3	H_Zn-L_Fe	H_Zn-L_Fe
159	24	PxW264 - 17	1452	0.309	24	R3	H_Zn-L_Fe	H_Zn-L_Fe
160	36	PxW546 - 24	1483	0.225	24	R3	H_Zn-L_Fe	H_Zn-L_Fe
162	33	PxW546 - 20	1486	0.308	24	R3	H_Zn-L_Fe	H_Zn-L_Fe
171	27	PxW273 - 71	1587	0.32	24	R3	H_Zn-L_Fe	H_Zn-L_Fe
172	82	PxW273 - 71	23	0.306	24	R1	H_Zn-L_Fe	H_Zn-L_Fe
176	76	PxW254 - 2	94	0.307	24	R1	H_Zn-L_Fe	H_Zn-L_Fe
178	88	PxW546 - 20	173	0.309	24	R1	H_Zn-L_Fe	H_Zn-L_Fe
184	91	PxW546 - 24	313	0.316	24	R1	H_Zn-L_Fe	H_Zn-L_Fe

185	79	PxW264 - 17	328	0.318	24	R1	H_Zn-L_Fe		H_Zn-L_Fe
189	85	PxW396 - 56	393	0.307	24	R1	H_Zn-L_Fe		H_Zn-L_Fe
192	92	PxW546 - 24	456	0.306	24	R2	H_Zn-L_Fe		H_Zn-L_Fe
193	80	PxW264 - 17	458	0.307	24	R2	H_Zn-L_Fe		H_Zn-L_Fe
197	77	PxW254 - 2	673	0.308	24	R2	H_Zn-L_Fe		H_Zn-L_Fe
200	89	PxW546 - 20	732	0.305	24	R2	H_Zn-L_Fe		H_Zn-L_Fe
202	86	PxW396 - 56	746	0.305	24	R2	H_Zn-L_Fe		H_Zn-L_Fe
207	83	PxW273 - 71	804	0.304	24	R2	H_Zn-L_Fe		H_Zn-L_Fe
211	84	PxW273 - 71	890	0.306	24	R3	H_Zn-L_Fe		H_Zn-L_Fe
212	81	PxW264 - 17	910	0.305	24	R3	H_Zn-L_Fe		H_Zn-L_Fe
214	90	PxW546 - 20	925	0.308	24	R3	H_Zn-L_Fe		H_Zn-L_Fe
215	93	PxW546 - 24	951	0.306	24	R3	H_Zn-L_Fe		H_Zn-L_Fe
218	87	PxW396 - 56	992	0.314	24	R3	H_Zn-L_Fe		H_Zn-L_Fe
223	78	PxW254 - 2	1072	0.31	24	R3	H_Zn-L_Fe		H_Zn-L_Fe
10	75	PxW216 - 88	120	0.307	15	R1	H_Zn-H_Fe	H_Zn-H_Fe	
12	16	PxW685 - 36	126	0.325	15	R3	H_Zn-H_Fe	H_Zn-H_Fe	
14	17	PxW811 - 30	140	0.308	15	R3	H_Zn-H_Fe	H_Zn-H_Fe	
15	74	PxW7-76	206	0.333	15	R1	H_Zn-H_Fe	H_Zn-H_Fe	
17	76	PxW685 - 36	215	0.306	15	R1	H_Zn-H_Fe	H_Zn-H_Fe	
21	80	PxW7-76	231	0.305	15	R2	H_Zn-H_Fe	H_Zn-H_Fe	
22	13	PxW7 - 2	247	0.316	15	R3	H_Zn-H_Fe	H_Zn-H_Fe	
23	87	PxW216 - 88	266	0.309	15	R3	H_Zn-H_Fe	H_Zn-H_Fe	
25	78	PxW811 - 83	288	0.301	15	R1	H_Zn-H_Fe	H_Zn-H_Fe	
28	82	PxW685 - 36	319	0.317	15	R2	H_Zn-H_Fe	H_Zn-H_Fe	
35	84	PxW811 - 83	385	0.317	15	R2	H_Zn-H_Fe	H_Zn-H_Fe	
38	3	PxW216 - 88	402	0.308	15	R1	H_Zn-H_Fe	H_Zn-H_Fe	
41	85	PxW7 - 2	421	0.306	15	R3	H_Zn-H_Fe	H_Zn-H_Fe	
49	2	PxW7-76	425	0.315	15	R1	H_Zn-H_Fe	H_Zn-H_Fe	
59	81	PxW216 - 88	531	0.312	15	R2	H_Zn-H_Fe	H_Zn-H_Fe	
60	77	PxW811 - 30	535	0.311	15	R1	H_Zn-H_Fe	H_Zn-H_Fe	
62	5	PxW811 - 30	557	0.308	15	R1	H_Zn-H_Fe	H_Zn-H_Fe	
66	90	PxW811 - 83	588	0.307	15	R3	H_Zn-H_Fe	H_Zn-H_Fe	
75	4	PxW685 - 36	654	0.294	15	R1	H_Zn-H_Fe	H_Zn-H_Fe	

80	88	PxW685 - 36	667	0.306	15	R3	H_Zn-H_Fe	H_Zn-H_Fe
82	7	PxW7 - 2	684	0.306	15	R2	H_Zn-H_Fe	H_Zn-H_Fe
90	79	PxW7 - 2	729	0.302	15	R2	H_Zn-H_Fe	H_Zn-H_Fe
91	83	PxW811 - 30	737	0.3	15	R2	H_Zn-H_Fe	H_Zn-H_Fe
98	12	PxW811 - 83	749	0.296	15	R2	H_Zn-H_Fe	H_Zn-H_Fe
100	9	PxW216 - 88	754	0.293	15	R2	H_Zn-H_Fe	H_Zn-H_Fe
101	89	PxW811 - 30	758	0.305	15	R3	H_Zn-H_Fe	H_Zn-H_Fe
102	86	PxW7-76	765	0.3	15	R3	H_Zn-H_Fe	H_Zn-H_Fe
106	73	PxW7 - 2	811	0.32	15	R1	H_Zn-H_Fe	H_Zn-H_Fe
110	14	PxW7-76	822	0.311	15	R3	H_Zn-H_Fe	H_Zn-H_Fe
116	18	PxW811 - 83	840	0.315	15	R3	H_Zn-H_Fe	H_Zn-H_Fe
117	15	PxW216 - 88	843	0.313	15	R3	H_Zn-H_Fe	H_Zn-H_Fe
120	8	PxW7-76	869	0.312	15	R2	H_Zn-H_Fe	H_Zn-H_Fe
126	10	PxW685 - 36	932	0.315	15	R2	H_Zn-H_Fe	H_Zn-H_Fe
127	11	PxW811 - 30	940	0.316	15	R2	H_Zn-H_Fe	H_Zn-H_Fe
132	1	PxW7 - 2	1013	0.306	15	R1	H_Zn-H_Fe	H_Zn-H_Fe
137	6	PxW811 - 83	1145	0.316	15	R1	H_Zn-H_Fe	H_Zn-H_Fe
154	75	PxW216 - 88	120	0.302	15	R1	H_Zn-H_Fe	H_Zn-H_Fe
156	16	PxW685 - 36	126	0.301	15	R3	H_Zn-H_Fe	H_Zn-H_Fe
158	17	PxW811 - 30	140	0.301	15	R3	H_Zn-H_Fe	H_Zn-H_Fe
159	74	PxW7-76	206	0.303	15	R1	H_Zn-H_Fe	H_Zn-H_Fe
161	76	PxW685 - 36	215	0.301	15	R1	H_Zn-H_Fe	H_Zn-H_Fe
165	80	PxW7-76	231	0.302	15	R2	H_Zn-H_Fe	H_Zn-H_Fe
166	13	PxW7 - 2	247	0.304	15	R3	H_Zn-H_Fe	H_Zn-H_Fe
167	87	PxW216 - 88	266	0.303	15	R3	H_Zn-H_Fe	H_Zn-H_Fe
169	78	PxW811 - 83	288	0.3	15	R1	H_Zn-H_Fe	H_Zn-H_Fe
172	82	PxW685 - 36	319	0.305	15	R2	H_Zn-H_Fe	H_Zn-H_Fe
179	84	PxW811 - 83	385	0.287	15	R2	H_Zn-H_Fe	H_Zn-H_Fe
182	3	PxW216 - 88	402	0.302	15	R1	H_Zn-H_Fe	H_Zn-H_Fe
185	85	PxW7 - 2	421	0.303	15	R3	H_Zn-H_Fe	H_Zn-H_Fe
193	2	PxW7-76	425	0.301	15	R1	H_Zn-H_Fe	H_Zn-H_Fe
203	81	PxW216 - 88	531	0.3	15	R2	H_Zn-H_Fe	H_Zn-H_Fe
204	77	PxW811 - 30	535	0.302	15	R1	H_Zn-H_Fe	H_Zn-H_Fe

206	5	PxW811 - 30	557	0.308	15	R1	H_Zn-H_Fe	H_Zn-H_Fe
210	90	PxW811 - 83	588	0.303	15	R3	H_Zn-H_Fe	H_Zn-H_Fe
219	4	PxW685 - 36	654	0.301	15	R1	H_Zn-H_Fe	H_Zn-H_Fe
224	88	PxW685 - 36	667	0.306	15	R3	H_Zn-H_Fe	H_Zn-H_Fe
226	7	PxW7 - 2	684	0.312	15	R2	H_Zn-H_Fe	H_Zn-H_Fe
234	79	PxW7 - 2	729	0.304	15	R2	H_Zn-H_Fe	H_Zn-H_Fe
235	83	PxW811 - 30	737	0.309	15	R2	H_Zn-H_Fe	H_Zn-H_Fe
242	12	PxW811 - 83	749	0.297	15	R2	H_Zn-H_Fe	H_Zn-H_Fe
244	9	PxW216 - 88	754	0.303	15	R2	H_Zn-H_Fe	H_Zn-H_Fe
245	89	PxW811 - 30	758	0.302	15	R3	H_Zn-H_Fe	H_Zn-H_Fe
246	86	PxW7-76	765	0.304	15	R3	H_Zn-H_Fe	H_Zn-H_Fe
250	73	PxW7 - 2	811	0.307	15	R1	H_Zn-H_Fe	H_Zn-H_Fe
254	14	PxW7-76	822	0.307	15	R3	H_Zn-H_Fe	H_Zn-H_Fe
260	18	PxW811 - 83	840	0.302	15	R3	H_Zn-H_Fe	H_Zn-H_Fe
261	15	PxW216 - 88	843	0.305	15	R3	H_Zn-H_Fe	H_Zn-H_Fe
264	8	PxW7-76	869	0.305	15	R2	H_Zn-H_Fe	H_Zn-H_Fe
270	10	PxW685 - 36	932	0.305	15	R2	H_Zn-H_Fe	H_Zn-H_Fe
271	11	PxW811 - 30	940	0.302	15	R2	H_Zn-H_Fe	H_Zn-H_Fe
276	1	PxW7 - 2	1013	0.304	15	R1	H_Zn-H_Fe	H_Zn-H_Fe
281	6	PxW811 - 83	1145	0.301	15	R1	H_Zn-H_Fe	H_Zn-H_Fe
3	1	PxW7 - 2	82	0.322	24	R1	H_Zn-H_Fe	H_Zn-H_Fe
4	7	PxW216 - 88	111	0.316	24	R1	H_Zn-H_Fe	H_Zn-H_Fe
7	13	PxW811 - 30	180	0.31	24	R1	H_Zn-H_Fe	H_Zn-H_Fe
10	4	PxW7-76	238	0.305	24	R1	H_Zn-H_Fe	H_Zn-H_Fe
18	10	PxW685 - 36	396	0.309	24	R1	H_Zn-H_Fe	H_Zn-H_Fe
19	16	PxW811 - 83	397	0.305	24	R1	H_Zn-H_Fe	H_Zn-H_Fe
22	8	PxW216 - 88	826	0.32	24	R2	H_Zn-H_Fe	H_Zn-H_Fe
23	14	PxW811 - 30	854	0.316	24	R2	H_Zn-H_Fe	H_Zn-H_Fe
27	11	PxW685 - 36	925	0.3	24	R2	H_Zn-H_Fe	H_Zn-H_Fe
30	2	PxW7 - 2	987	0.308	24	R2	H_Zn-H_Fe	H_Zn-H_Fe
34	5	PxW7-76	1075	0.31	24	R2	H_Zn-H_Fe	H_Zn-H_Fe
37	17	PxW811 - 83	1156	0.308	24	R2	H_Zn-H_Fe	H_Zn-H_Fe
41	9	PxW216 - 88	1274	0.318	24	R3	H_Zn-H_Fe	H_Zn-H_Fe

47	3	PxW7 - 2	1484	0.32	24	R3	H_Zn-H_Fe	H_Zn-H_Fe
49	6	PxW7-76	1494	0.324	24	R3	H_Zn-H_Fe	H_Zn-H_Fe
50	15	PxW811 - 30	1511	0.304	24	R3	H_Zn-H_Fe	H_Zn-H_Fe
51	12	PxW685 - 36	1528	0.317	24	R3	H_Zn-H_Fe	H_Zn-H_Fe
54	18	PxW811 - 83	1548	0.302	24	R3	H_Zn-H_Fe	H_Zn-H_Fe
61	70	PxW811 - 30	42	0.322	24	R1	H_Zn-H_Fe	H_Zn-H_Fe
66	58	PxW7 - 2	212	0.315	24	R1	H_Zn-H_Fe	H_Zn-H_Fe
68	73	PxW811 - 83	273	0.308	24	R1	H_Zn-H_Fe	H_Zn-H_Fe
72	61	PxW7-76	348	0.321	24	R1	H_Zn-H_Fe	H_Zn-H_Fe
74	67	PxW685 - 36	373	0.301	24	R1	H_Zn-H_Fe	H_Zn-H_Fe
76	64	PxW216 - 88	400	0.317	24	R1	H_Zn-H_Fe	H_Zn-H_Fe
77	62	PxW7-76	415	0.306	24	R2	H_Zn-H_Fe	H_Zn-H_Fe
81	68	PxW685 - 36	626	0.323	24	R2	H_Zn-H_Fe	H_Zn-H_Fe
84	59	PxW7 - 2	721	0.317	24	R2	H_Zn-H_Fe	H_Zn-H_Fe
87	74	PxW811 - 83	733	0.308	24	R2	H_Zn-H_Fe	H_Zn-H_Fe
89	65	PxW216 - 88	762	0.309	24	R2	H_Zn-H_Fe	H_Zn-H_Fe
103	75	PxW811 - 83	974	0.309	24	R3	H_Zn-H_Fe	H_Zn-H_Fe
106	66	PxW216 - 88	1055	0.311	24	R3	H_Zn-H_Fe	H_Zn-H_Fe
107	69	PxW685 - 36	1060	0.313	24	R3	H_Zn-H_Fe	H_Zn-H_Fe
108	63	PxW7-76	1070	0.296	24	R3	H_Zn-H_Fe	H_Zn-H_Fe
111	60	PxW7 - 2	1149	0.3	24	R3	H_Zn-H_Fe	H_Zn-H_Fe
113	71	PxW811 - 30	1161	0.315	24	R2	H_Zn-H_Fe	H_Zn-H_Fe
114	72	PxW811 - 30		0.315	24	R3	H_Zn-H_Fe	H_Zn-H_Fe
117	1	PxW7 - 2	82	0.316	24	R1	H_Zn-H_Fe	H_Zn-H_Fe
118	7	PxW216 - 88	111	0.31	24	R1	H_Zn-H_Fe	H_Zn-H_Fe
121	13	PxW811 - 30	180	0.31	24	R1	H_Zn-H_Fe	H_Zn-H_Fe
124	4	PxW7-76	238	0.313	24	R1	H_Zn-H_Fe	H_Zn-H_Fe
132	10	PxW685 - 36	396	0.311	24	R1	H_Zn-H_Fe	H_Zn-H_Fe
133	16	PxW811 - 83	397	0.286	24	R1	H_Zn-H_Fe	H_Zn-H_Fe
136	8	PxW216 - 88	826	0.307	24	R2	H_Zn-H_Fe	H_Zn-H_Fe
137	14	PxW811 - 30	854	0.313	24	R2	H_Zn-H_Fe	H_Zn-H_Fe
141	11	PxW685 - 36	925	0.301	24	R2	H_Zn-H_Fe	H_Zn-H_Fe
144	2	PxW7 - 2	987	0.31	24	R2	H_Zn-H_Fe	H_Zn-H_Fe

148	5	PxW7-76	1075	0.305	24	R2	H_Zn-H_Fe	H_Zn-H_Fe
151	17	PxW811 - 83	1156	0.314	24	R2	H_Zn-H_Fe	H_Zn-H_Fe
155	9	PxW216 - 88	1274	0.312	24	R3	H_Zn-H_Fe	H_Zn-H_Fe
161	3	PxW7 - 2	1484	0.301	24	R3	H_Zn-H_Fe	H_Zn-H_Fe
163	6	PxW7-76	1494	0.3	24	R3	H_Zn-H_Fe	H_Zn-H_Fe
164	15	PxW811 - 30	1511	0.305	24	R3	H_Zn-H_Fe	H_Zn-H_Fe
165	12	PxW685 - 36	1528	0.306	24	R3	H_Zn-H_Fe	H_Zn-H_Fe
168	18	PxW811 - 83	1548	0.304	24	R3	H_Zn-H_Fe	H_Zn-H_Fe
175	70	PxW811 - 30	42	0.306	24	R1	H_Zn-H_Fe	H_Zn-H_Fe
180	58	PxW7 - 2	212	0.309	24	R1	H_Zn-H_Fe	H_Zn-H_Fe
182	73	PxW811 - 83	273	0.304	24	R1	H_Zn-H_Fe	H_Zn-H_Fe
186	61	PxW7-76	348	0.317	24	R1	H_Zn-H_Fe	H_Zn-H_Fe
188	67	PxW685 - 36	373	0.307	24	R1	H_Zn-H_Fe	H_Zn-H_Fe
190	64	PxW216 - 88	400	0.304	24	R1	H_Zn-H_Fe	H_Zn-H_Fe
191	62	PxW7-76	415	0.307	24	R2	H_Zn-H_Fe	H_Zn-H_Fe
195	68	PxW685 - 36	626	0.308	24	R2	H_Zn-H_Fe	H_Zn-H_Fe
198	59	PxW7 - 2	721	0.304	24	R2	H_Zn-H_Fe	H_Zn-H_Fe
201	74	PxW811 - 83	733	0.306	24	R2	H_Zn-H_Fe	H_Zn-H_Fe
203	65	PxW216 - 88	762	0.314	24	R2	H_Zn-H_Fe	H_Zn-H_Fe
217	75	PxW811 - 83	974	0.304	24	R3	H_Zn-H_Fe	H_Zn-H_Fe
220	66	PxW216 - 88	1055	0.313	24	R3	H_Zn-H_Fe	H_Zn-H_Fe
221	69	PxW685 - 36	1060	0.308	24	R3	H_Zn-H_Fe	H_Zn-H_Fe
222	63	PxW7-76	1070	0.316	24	R3	H_Zn-H_Fe	H_Zn-H_Fe
225	60	PxW7 - 2	1149	0.309	24	R3	H_Zn-H_Fe	H_Zn-H_Fe
227	71	PxW811 - 30	1161	0.306	24	R2	H_Zn-H_Fe	H_Zn-H_Fe
228	72	PxW811 - 30		0.309	24	R3	H_Zn-H_Fe	H_Zn-H_Fe

r two years, 2015-19.

L_Zn-H_Fe!	L_Zn-L_Fe!	Site!	UoN!	Rres!	Year!	Fraction!	Grain!	Endo!	Year_2016!	Year_2017!	Fe_mg_kg_Grain	Zn_mg_kg_Grain
	L_Zn-L_Fe	Rres		Rres	2016	Grain	Grain		2016		22.69522109	29.48632052
	L_Zn-L_Fe	Rres		Rres	2016	Grain	Grain		2016		25.67474052	28.44821471
	L_Zn-L_Fe	Rres		Rres	2016	Grain	Grain		2016		22.67057334	29.69343876
	L_Zn-L_Fe	Rres		Rres	2016	Grain	Grain		2016		23.88256118	32.67052589
	L_Zn-L_Fe	UoN	UoN		2016	Grain	Grain		2016		21.28275389	25.06232415
	L_Zn-L_Fe	Rres		Rres	2016	Grain	Grain		2016		26.99365805	34.91280812
	L_Zn-L_Fe	Rres		Rres	2016	Grain	Grain		2016		23.7530743	31.89595909
	L_Zn-L_Fe	UoN	UoN		2016	Grain	Grain		2016		24.94879445	23.81109339
	L_Zn-L_Fe	Rres		Rres	2016	Grain	Grain		2016		25.26153719	27.85839456
	L_Zn-L_Fe	Rres		Rres	2016	Grain	Grain		2016		27.36109399	40.68573739
	L_Zn-L_Fe	UoN	UoN		2016	Grain	Grain		2016		22.20248246	24.04954278
	L_Zn-L_Fe	UoN	UoN		2016	Grain	Grain		2016		30.06769449	28.05085556
	L_Zn-L_Fe	UoN	UoN		2016	Grain	Grain		2016		29.02084879	31.50887762
	L_Zn-L_Fe	Rres		Rres	2016	Grain	Grain		2016		24.92012176	27.65929684
	L_Zn-L_Fe	Rres		Rres	2016	Grain	Grain		2016		36.31528891	26.46445222
	L_Zn-L_Fe	UoN	UoN		2016	Grain	Grain		2016		29.04542043	21.38031744
	L_Zn-L_Fe	Rres		Rres	2016	Grain	Grain		2016		36.39970084	26.76455519
	L_Zn-L_Fe	Rres		Rres	2016	Grain	Grain		2016		24.81701978	27.74806985
	L_Zn-L_Fe	UoN	UoN		2016	Grain	Grain		2016		23.84999665	19.09536271
	L_Zn-L_Fe	Rres		Rres	2016	Grain	Grain		2016		24.39593985	30.14362966
	L_Zn-L_Fe	UoN	UoN		2016	Grain	Grain		2016		31.78993225	25.82745549
	L_Zn-L_Fe	Rres		Rres	2016	Grain	Grain		2016		23.02805908	31.40666746
	L_Zn-L_Fe	Rres		Rres	2016	Grain	Grain		2016		24.72586409	30.42349948
	L_Zn-L_Fe	UoN	UoN		2016	Grain	Grain		2016			
	L_Zn-L_Fe	UoN	UoN		2016	Grain	Grain		2016		32.29938747	27.94149198
	L_Zn-L_Fe	UoN	UoN		2016	Grain	Grain		2016		24.64443832	22.41583377
	L_Zn-L_Fe	Rres		Rres	2016	Grain	Grain		2016		25.68955948	27.79200971
	L_Zn-L_Fe	UoN	UoN		2016	Grain	Grain		2016		27.20373157	23.46492484
	L_Zn-L_Fe	Rres		Rres	2016	Grain	Grain		2016		21.12827131	31.74933483
	L_Zn-L_Fe	UoN	UoN		2016	Grain	Grain		2016		22.46301686	20.97923365
	L_Zn-L_Fe	UoN	UoN		2016	Grain	Grain		2016		32.06948482	21.44134261

L_Zn-L_Fe	Rres		Rres	2016	Grain	Grain		2016	39.89334981	39.67551237
L_Zn-L_Fe	UoN	UoN		2016	Grain	Grain		2016	26.70942851	24.66196949
L_Zn-L_Fe	UoN	UoN		2016	Grain	Grain		2016	28.30037725	22.69896571
L_Zn-L_Fe	UoN	UoN		2016	Grain	Grain		2016	24.16031367	23.19772888
L_Zn-L_Fe	UoN	UoN		2016	Grain	Grain		2016	27.8256625	27.22579805
L_Zn-L_Fe	Rres		Rres	2016	Endo		Endo	2016		
L_Zn-L_Fe	Rres		Rres	2016	Endo		Endo	2016		
L_Zn-L_Fe	Rres		Rres	2016	Endo		Endo	2016		
L_Zn-L_Fe	Rres		Rres	2016	Endo		Endo	2016		
L_Zn-L_Fe	UoN	UoN		2016	Endo		Endo	2016		
L_Zn-L_Fe	Rres		Rres	2016	Endo		Endo	2016		
L_Zn-L_Fe	Rres		Rres	2016	Endo		Endo	2016		
L_Zn-L_Fe	UoN	UoN		2016	Endo		Endo	2016		
L_Zn-L_Fe	Rres		Rres	2016	Endo		Endo	2016		
L_Zn-L_Fe	Rres		Rres	2016	Endo		Endo	2016		
L_Zn-L_Fe	UoN	UoN		2016	Endo		Endo	2016		
L_Zn-L_Fe	UoN	UoN		2016	Endo		Endo	2016		
L_Zn-L_Fe	UoN	UoN		2016	Endo		Endo	2016		
L_Zn-L_Fe	Rres		Rres	2016	Endo		Endo	2016		
L_Zn-L_Fe	Rres		Rres	2016	Endo		Endo	2016		
L_Zn-L_Fe	UoN	UoN		2016	Endo		Endo	2016		
L_Zn-L_Fe	Rres		Rres	2016	Endo		Endo	2016		
L_Zn-L_Fe	Rres		Rres	2016	Endo		Endo	2016		
L_Zn-L_Fe	UoN	UoN		2016	Endo		Endo	2016		
L_Zn-L_Fe	Rres		Rres	2016	Endo		Endo	2016		
L_Zn-L_Fe	UoN	UoN		2016	Endo		Endo	2016		
L_Zn-L_Fe	Rres		Rres	2016	Endo		Endo	2016		
L_Zn-L_Fe	Rres		Rres	2016	Endo		Endo	2016		
L_Zn-L_Fe	UoN	UoN		2016	Endo		Endo	2016		
L_Zn-L_Fe	UoN	UoN		2016	Endo		Endo	2016		
L_Zn-L_Fe	UoN	UoN		2016	Endo		Endo	2016		
L_Zn-L_Fe	Rres		Rres	2016	Endo		Endo	2016		
L_Zn-L_Fe	UoN	UoN		2016	Endo		Endo	2016		

L_Zn-L_Fe	Rres		Rres	2016	Endo		Endo	2016				
L_Zn-L_Fe	UoN	UoN		2016	Endo		Endo	2016				
L_Zn-L_Fe	UoN	UoN		2016	Endo		Endo	2016				
L_Zn-L_Fe	Rres		Rres	2016	Endo		Endo	2016				
L_Zn-L_Fe	UoN	UoN		2016	Endo		Endo	2016				
L_Zn-L_Fe	UoN	UoN		2016	Endo		Endo	2016				
L_Zn-L_Fe	UoN	UoN		2016	Endo		Endo	2016				
L_Zn-L_Fe	UoN	UoN		2016	Endo		Endo	2016				
L_Zn-L_Fe	UoN	UoN		2017	Grain	Grain			2017	30.07417109	17.79979346	
L_Zn-L_Fe	UoN	UoN		2017	Grain	Grain			2017	40.31442228	25.89472986	
L_Zn-L_Fe	UoN	UoN		2017	Grain	Grain			2017	32.07397779	15.32084553	
L_Zn-L_Fe	UoN	UoN		2017	Grain	Grain			2017	33.56625384	17.36792378	
L_Zn-L_Fe	UoN	UoN		2017	Grain	Grain			2017	32.78359911	17.90209758	
L_Zn-L_Fe	UoN	UoN		2017	Grain	Grain			2017	25.94437028	18.89560489	
L_Zn-L_Fe	UoN	UoN		2017	Grain	Grain			2017	37.66732945	21.93565246	
L_Zn-L_Fe	UoN	UoN		2017	Grain	Grain			2017	38.18221135	21.81557995	
L_Zn-L_Fe	UoN	UoN		2017	Grain	Grain			2017	46.32969182	19.21172553	
L_Zn-L_Fe	UoN	UoN		2017	Grain	Grain			2017	29.33958348	15.21501112	
L_Zn-L_Fe	UoN	UoN		2017	Grain	Grain			2017	31.82190453	20.14626818	
L_Zn-L_Fe	UoN	UoN		2017	Grain	Grain			2017	22.49417944	22.19522251	
L_Zn-L_Fe	UoN	UoN		2017	Grain	Grain			2017	37.18190388	20.29280738	
L_Zn-L_Fe	UoN	UoN		2017	Grain	Grain			2017	35.25669272	19.08021398	
L_Zn-L_Fe	UoN	UoN		2017	Grain	Grain			2017	35.48098029	22.88372256	
L_Zn-L_Fe	UoN	UoN		2017	Grain	Grain			2017	27.9663525	15.94407546	
L_Zn-L_Fe	UoN	UoN		2017	Grain	Grain			2017	30.99375469	16.77033315	
L_Zn-L_Fe	UoN	UoN		2017	Grain	Grain			2017	28.68602928	17.07834081	
L_Zn-L_Fe	Rres		Rres	2017	Grain	Grain			2017	31.97848145	25.67834961	
L_Zn-L_Fe	Rres		Rres	2017	Grain	Grain			2017	46.66612352	44.29021463	
L_Zn-L_Fe	Rres		Rres	2017	Grain	Grain			2017	36.93632664	31.30822265	
L_Zn-L_Fe	Rres		Rres	2017	Grain	Grain			2017	53.56069917	48.34763171	
L_Zn-L_Fe	Rres		Rres	2017	Grain	Grain			2017	41.63117053	41.48569624	
L_Zn-L_Fe	Rres		Rres	2017	Grain	Grain			2017	47.09011959	34.47957517	
L_Zn-L_Fe	Rres		Rres	2017	Grain	Grain			2017	41.95440663	41.30098688	

L_Zn-L_Fe	Rres		Rres	2017	Grain	Grain		2017	37.71714401	33.81685217
L_Zn-L_Fe	Rres		Rres	2017	Grain	Grain		2017	48.32257052	43.71340373
L_Zn-L_Fe	Rres		Rres	2017	Grain	Grain		2017	36.75068397	32.74869469
L_Zn-L_Fe	Rres		Rres	2017	Grain	Grain		2017	40.5598706	33.77779611
L_Zn-L_Fe	Rres		Rres	2017	Grain	Grain		2017	41.33888576	27.13063337
L_Zn-L_Fe	Rres		Rres	2017	Grain	Grain		2017	43.34895359	47.82135316
L_Zn-L_Fe	Rres		Rres	2017	Grain	Grain		2017	35.38868528	27.28408078
L_Zn-L_Fe	Rres		Rres	2017	Grain	Grain		2017	49.42641646	35.85519323
L_Zn-L_Fe	Rres		Rres	2017	Grain	Grain		2017	42.60351942	35.76517442
L_Zn-L_Fe	Rres		Rres	2017	Grain	Grain		2017	37.11474754	32.40550231
L_Zn-L_Fe	Rres		Rres	2017	Grain	Grain		2017	48.00819792	38.41378172
L_Zn-L_Fe	UoN	UoN		2017	Endo		Endo	2017		
L_Zn-L_Fe	UoN	UoN		2017	Endo		Endo	2017		
L_Zn-L_Fe	UoN	UoN		2017	Endo		Endo	2017		
L_Zn-L_Fe	UoN	UoN		2017	Endo		Endo	2017		
L_Zn-L_Fe	UoN	UoN		2017	Endo		Endo	2017		
L_Zn-L_Fe	UoN	UoN		2017	Endo		Endo	2017		
L_Zn-L_Fe	UoN	UoN		2017	Endo		Endo	2017		
L_Zn-L_Fe	UoN	UoN		2017	Endo		Endo	2017		
L_Zn-L_Fe	UoN	UoN		2017	Endo		Endo	2017		
L_Zn-L_Fe	UoN	UoN		2017	Endo		Endo	2017		
L_Zn-L_Fe	UoN	UoN		2017	Endo		Endo	2017		
L_Zn-L_Fe	UoN	UoN		2017	Endo		Endo	2017		
L_Zn-L_Fe	UoN	UoN		2017	Endo		Endo	2017		
L_Zn-L_Fe	UoN	UoN		2017	Endo		Endo	2017		
L_Zn-L_Fe	UoN	UoN		2017	Endo		Endo	2017		
L_Zn-L_Fe	UoN	UoN		2017	Endo		Endo	2017		
L_Zn-L_Fe	UoN	UoN		2017	Endo		Endo	2017		
L_Zn-L_Fe	UoN	UoN		2017	Endo		Endo	2017		
L_Zn-L_Fe	Rres		Rres	2017	Endo		Endo	2017		
L_Zn-L_Fe	Rres		Rres	2017	Endo		Endo	2017		
L_Zn-L_Fe	Rres		Rres	2017	Endo		Endo	2017		
L_Zn-L_Fe	Rres		Rres	2017	Endo		Endo	2017		

	L_Zn-L_Fe	Rres		Rres	2017	Endo		Endo		2017		
	L_Zn-L_Fe	Rres		Rres	2017	Endo		Endo		2017		
	L_Zn-L_Fe	Rres		Rres	2017	Endo		Endo		2017		
	L_Zn-L_Fe	Rres		Rres	2017	Endo		Endo		2017		
	L_Zn-L_Fe	Rres		Rres	2017	Endo		Endo		2017		
	L_Zn-L_Fe	Rres		Rres	2017	Endo		Endo		2017		
	L_Zn-L_Fe	Rres		Rres	2017	Endo		Endo		2017		
	L_Zn-L_Fe	Rres		Rres	2017	Endo		Endo		2017		
	L_Zn-L_Fe	Rres		Rres	2017	Endo		Endo		2017		
	L_Zn-L_Fe	Rres		Rres	2017	Endo		Endo		2017		
	L_Zn-L_Fe	Rres		Rres	2017	Endo		Endo		2017		
	L_Zn-L_Fe	Rres		Rres	2017	Endo		Endo		2017		
	L_Zn-L_Fe	Rres		Rres	2017	Endo		Endo		2017		
	L_Zn-L_Fe	Rres		Rres	2017	Endo		Endo		2017		
L_Zn-H_Fe		UoN	UoN		2016	Grain	Grain		2016		32.08460269	28.22305068
L_Zn-H_Fe		UoN	UoN		2016	Grain	Grain		2016		34.13814742	25.50909861
L_Zn-H_Fe		UoN	UoN		2016	Grain	Grain		2016		41.97029387	27.93451671
L_Zn-H_Fe		Rres		Rres	2016	Grain	Grain		2016		33.91916986	44.64234667
L_Zn-H_Fe		UoN	UoN		2016	Grain	Grain		2016		35.67666641	23.02870461
L_Zn-H_Fe		UoN	UoN		2016	Grain	Grain		2016		33.63213477	28.08600497
L_Zn-H_Fe		UoN	UoN		2016	Grain	Grain		2016		32.78871128	28.03828919
L_Zn-H_Fe		UoN	UoN		2016	Grain	Grain		2016		34.03422446	32.33524224
L_Zn-H_Fe		UoN	UoN		2016	Grain	Grain		2016		36.42826738	28.80351312
L_Zn-H_Fe		UoN	UoN		2016	Grain	Grain		2016		30.84383293	24.16213607
L_Zn-H_Fe		UoN	UoN		2016	Grain	Grain		2016		27.00089197	31.79845529
L_Zn-H_Fe		UoN	UoN		2016	Grain	Grain		2016		28.7424174	28.993622
L_Zn-H_Fe		UoN	UoN		2016	Grain	Grain		2016		34.98020846	24.65186142
L_Zn-H_Fe		UoN	UoN		2016	Grain	Grain		2016		30.74019963	33.21926904
L_Zn-H_Fe		Rres		Rres	2016	Grain	Grain		2016		32.71767186	40.74513414
L_Zn-H_Fe		UoN	UoN		2016	Grain	Grain		2016		29.72495566	30.51868412
L_Zn-H_Fe		Rres		Rres	2016	Grain	Grain		2016		29.71783565	40.5861331
L_Zn-H_Fe		UoN	UoN		2016	Grain	Grain		2016		27.28379021	23.7866808
L_Zn-H_Fe		UoN	UoN		2016	Grain	Grain		2016		37.73128162	33.10536945

L_Zn-H_Fe	UoN	UoN		2016	Grain	Grain		2016		27.37598537	24.24917888
L_Zn-H_Fe	UoN	UoN		2016	Grain	Grain		2016		32.04853161	28.81454927
L_Zn-H_Fe	UoN	UoN		2016	Endo		Endo	2016			
L_Zn-H_Fe	UoN	UoN		2016	Endo		Endo	2016			
L_Zn-H_Fe	UoN	UoN		2016	Endo		Endo	2016			
L_Zn-H_Fe	Rres		Rres	2016	Endo		Endo	2016			
L_Zn-H_Fe	UoN	UoN		2016	Endo		Endo	2016			
L_Zn-H_Fe	UoN	UoN		2016	Endo		Endo	2016			
L_Zn-H_Fe	UoN	UoN		2016	Endo		Endo	2016			
L_Zn-H_Fe	UoN	UoN		2016	Endo		Endo	2016			
L_Zn-H_Fe	UoN	UoN		2016	Endo		Endo	2016			
L_Zn-H_Fe	UoN	UoN		2016	Endo		Endo	2016			
L_Zn-H_Fe	UoN	UoN		2016	Endo		Endo	2016			
L_Zn-H_Fe	UoN	UoN		2016	Endo		Endo	2016			
L_Zn-H_Fe	UoN	UoN		2016	Endo		Endo	2016			
L_Zn-H_Fe	UoN	UoN		2016	Endo		Endo	2016			
L_Zn-H_Fe	Rres		Rres	2016	Endo		Endo	2016			
L_Zn-H_Fe	UoN	UoN		2016	Endo		Endo	2016			
L_Zn-H_Fe	Rres		Rres	2016	Endo		Endo	2016			
L_Zn-H_Fe	UoN	UoN		2016	Endo		Endo	2016			
L_Zn-H_Fe	UoN	UoN		2016	Endo		Endo	2016			
L_Zn-H_Fe	UoN	UoN		2016	Endo		Endo	2016			
L_Zn-H_Fe	UoN	UoN		2016	Endo		Endo	2016			
L_Zn-H_Fe	UoN	UoN		2017	Grain	Grain			2017	33.46908976	20.53158639
L_Zn-H_Fe	UoN	UoN		2017	Grain	Grain			2017	37.560348	20.72390267
L_Zn-H_Fe	UoN	UoN		2017	Grain	Grain			2017	35.17823846	18.00326162
L_Zn-H_Fe	Rres		Rres	2017	Grain	Grain			2017	50.96782353	43.4586841
L_Zn-H_Fe	Rres		Rres	2017	Grain	Grain			2017	43.04281915	33.17588814
L_Zn-H_Fe	Rres		Rres	2017	Grain	Grain			2017	38.37471985	39.7511835
L_Zn-H_Fe	UoN	UoN		2017	Endo		Endo		2017		
L_Zn-H_Fe	UoN	UoN		2017	Endo		Endo		2017		
L_Zn-H_Fe	UoN	UoN		2017	Endo		Endo		2017		
L_Zn-H_Fe	Rres		Rres	2017	Endo		Endo		2017		

L_Zn-H_Fe	Rres		Rres	2017	Endo		Endo		2017		
L_Zn-H_Fe	Rres		Rres	2017	Endo		Endo		2017		
	Rres		Rres	2016	Grain	Grain		2016		25.84113829	37.57793347
	UoN	UoN		2016	Grain	Grain		2016		26.29923104	22.51965675
	Rres		Rres	2016	Grain	Grain		2016		24.55963283	39.81864457
	UoN	UoN		2016	Grain	Grain		2016		27.8983113	38.0579565
	Rres		Rres	2016	Grain	Grain		2016		26.62409137	34.76247408
	UoN	UoN		2016	Grain	Grain		2016		41.32127341	52.42744366
	UoN	UoN		2016	Grain	Grain		2016		26.05968284	27.60640241
	UoN	UoN		2016	Grain	Grain		2016		30.25958004	38.38385169
	Rres		Rres	2016	Grain	Grain		2016		21.38032982	37.52398015
	Rres		Rres	2016	Grain	Grain		2016		27.88172225	43.23305844
	Rres		Rres	2016	Grain	Grain		2016		24.6344677	38.17690432
	UoN	UoN		2016	Grain	Grain		2016		24.95913088	30.96771019
	Rres		Rres	2016	Grain	Grain		2016		25.30930009	38.10240387
	Rres		Rres	2016	Grain	Grain		2016		31.23323036	39.08229754
	UoN	UoN		2016	Grain	Grain		2016		26.20239252	23.35698759
	UoN	UoN		2016	Grain	Grain		2016		27.1336884	32.92972359
	UoN	UoN		2016	Grain	Grain		2016		32.86476577	34.74039917
	UoN	UoN		2016	Grain	Grain		2016		36.59008779	43.16811582
	Rres		Rres	2016	Grain	Grain		2016		27.11834384	38.3022376
	Rres		Rres	2016	Grain	Grain		2016		26.01336917	40.29513114
	UoN	UoN		2016	Grain	Grain		2016			
	Rres		Rres	2016	Grain	Grain		2016		27.58970575	42.23585281
	UoN	UoN		2016	Grain	Grain		2016		30.45321884	36.35255219
	UoN	UoN		2016	Grain	Grain		2016		25.7334148	32.48343651
	UoN	UoN		2016	Grain	Grain		2016		30.9825871	24.41986135
	UoN	UoN		2016	Grain	Grain		2016		44.66847598	34.41475637
	Rres		Rres	2016	Grain	Grain		2016		27.92277498	46.46762583
	Rres		Rres	2016	Grain	Grain		2016		29.49734488	46.09654774
	UoN	UoN		2016	Grain	Grain		2016		25.42385732	21.19172106
	Rres		Rres	2016	Grain	Grain		2016		37.21171516	48.58414795
	Rres		Rres	2016	Grain	Grain		2016		22.92387603	39.35932354

Rres		Rres	2016	Grain	Grain		2016	26.49930798	36.74587027
Rres		Rres	2016	Grain	Grain		2016	26.98075469	41.9311521
Rres		Rres	2016	Grain	Grain		2016	35.07731097	64.75374197
UoN	UoN		2016	Grain	Grain		2016	31.1107635	30.5429708
UoN	UoN		2016	Grain	Grain		2016	23.53577053	28.57662565
Rres		Rres	2016	Endo		Endo	2016		
UoN	UoN		2016	Endo		Endo	2016		
Rres		Rres	2016	Endo		Endo	2016		
UoN	UoN		2016	Endo		Endo	2016		
Rres		Rres	2016	Endo		Endo	2016		
UoN	UoN		2016	Endo		Endo	2016		
UoN	UoN		2016	Endo		Endo	2016		
UoN	UoN		2016	Endo		Endo	2016		
Rres		Rres	2016	Endo		Endo	2016		
Rres		Rres	2016	Endo		Endo	2016		
Rres		Rres	2016	Endo		Endo	2016		
UoN	UoN		2016	Endo		Endo	2016		
Rres		Rres	2016	Endo		Endo	2016		
Rres		Rres	2016	Endo		Endo	2016		
UoN	UoN		2016	Endo		Endo	2016		
UoN	UoN		2016	Endo		Endo	2016		
UoN	UoN		2016	Endo		Endo	2016		
UoN	UoN		2016	Endo		Endo	2016		
Rres		Rres	2016	Endo		Endo	2016		
Rres		Rres	2016	Endo		Endo	2016		
UoN	UoN		2016	Endo		Endo	2016		
Rres		Rres	2016	Endo		Endo	2016		
UoN	UoN		2016	Endo		Endo	2016		
UoN	UoN		2016	Endo		Endo	2016		
UoN	UoN		2016	Endo		Endo	2016		
UoN	UoN		2016	Endo		Endo	2016		
Rres		Rres	2016	Endo		Endo	2016		
Rres		Rres	2016	Endo		Endo	2016		

UoN	UoN		2016	Endo		Endo	2016			
Rres		Rres	2016	Endo		Endo	2016			
Rres		Rres	2016	Endo		Endo	2016			
Rres		Rres	2016	Endo		Endo	2016			
Rres		Rres	2016	Endo		Endo	2016			
Rres		Rres	2016	Endo		Endo	2016			
UoN	UoN		2016	Endo		Endo	2016			
UoN	UoN		2016	Endo		Endo	2016			
UoN	UoN		2017	Grain	Grain			2017	33.73487116	29.64745613
UoN	UoN		2017	Grain	Grain			2017	33.17642043	21.16932725
UoN	UoN		2017	Grain	Grain			2017	34.34132504	23.48886267
UoN	UoN		2017	Grain	Grain			2017	41.0897638	29.99860912
UoN	UoN		2017	Grain	Grain			2017	33.58171624	23.79084193
UoN	UoN		2017	Grain	Grain			2017	34.71502254	18.88778065
UoN	UoN		2017	Grain	Grain			2017	38.42442154	24.02061496
UoN	UoN		2017	Grain	Grain			2017	39.81695851	25.34268113
UoN	UoN		2017	Grain	Grain			2017	32.79024859	25.75589478
UoN	UoN		2017	Grain	Grain			2017	31.6767245	20.56576045
UoN	UoN		2017	Grain	Grain			2017	36.37311591	23.60354348
UoN	UoN		2017	Grain	Grain			2017	33.24781746	30.33717216
UoN	UoN		2017	Grain	Grain			2017	39.60489736	25.74237198
UoN	UoN		2017	Grain	Grain			2017	37.97616585	24.5916331
UoN	UoN		2017	Grain	Grain			2017	29.95098214	21.24352221
UoN	UoN		2017	Grain	Grain			2017	33.16202163	26.52460513
UoN	UoN		2017	Grain	Grain			2017	33.60666083	29.17759188
UoN	UoN		2017	Grain	Grain			2017	34.83426559	22.4392036
Rres		Rres	2017	Grain	Grain			2017	40.56124029	35.02342518
Rres		Rres	2017	Grain	Grain			2017	38.8896309	35.1532042
Rres		Rres	2017	Grain	Grain			2017	51.43920322	49.00016066
Rres		Rres	2017	Grain	Grain			2017	46.7728925	55.59784742
Rres		Rres	2017	Grain	Grain			2017	52.74156456	52.37690601
Rres		Rres	2017	Grain	Grain			2017	53.35675317	52.38916027
Rres		Rres	2017	Grain	Grain			2017	43.9371665	44.18362759

Rres		Rres	2017	Grain	Grain		2017	46.259078	53.52474888
Rres		Rres	2017	Grain	Grain		2017	36.35537518	29.29501716
Rres		Rres	2017	Grain	Grain		2017	42.89839332	43.35869844
Rres		Rres	2017	Grain	Grain		2017	51.42195309	50.94219241
Rres		Rres	2017	Grain	Grain		2017	42.3258847	37.07967466
Rres		Rres	2017	Grain	Grain		2017	53.1377236	44.23481046
Rres		Rres	2017	Grain	Grain		2017	49.38734494	54.66475545
Rres		Rres	2017	Grain	Grain		2017	47.24649639	45.91703038
Rres		Rres	2017	Grain	Grain		2017	43.54318686	56.58179579
Rres		Rres	2017	Grain	Grain		2017	48.08040761	41.37447388
Rres		Rres	2017	Grain	Grain		2017	37.98259574	33.10061153
UoN	UoN		2017	Endo		Endo	2017		
UoN	UoN		2017	Endo		Endo	2017		
UoN	UoN		2017	Endo		Endo	2017		
UoN	UoN		2017	Endo		Endo	2017		
UoN	UoN		2017	Endo		Endo	2017		
UoN	UoN		2017	Endo		Endo	2017		
UoN	UoN		2017	Endo		Endo	2017		
UoN	UoN		2017	Endo		Endo	2017		
UoN	UoN		2017	Endo		Endo	2017		
UoN	UoN		2017	Endo		Endo	2017		
UoN	UoN		2017	Endo		Endo	2017		
UoN	UoN		2017	Endo		Endo	2017		
UoN	UoN		2017	Endo		Endo	2017		
UoN	UoN		2017	Endo		Endo	2017		
UoN	UoN		2017	Endo		Endo	2017		
UoN	UoN		2017	Endo		Endo	2017		
UoN	UoN		2017	Endo		Endo	2017		
UoN	UoN		2017	Endo		Endo	2017		
Rres		Rres	2017	Endo		Endo	2017		
Rres		Rres	2017	Endo		Endo	2017		
Rres		Rres	2017	Endo		Endo	2017		
Rres		Rres	2017	Endo		Endo	2017		

Rres		Rres	2017	Endo		Endo		2017		
Rres		Rres	2017	Endo		Endo		2017		
Rres		Rres	2017	Endo		Endo		2017		
Rres		Rres	2017	Endo		Endo		2017		
Rres		Rres	2017	Endo		Endo		2017		
Rres		Rres	2017	Endo		Endo		2017		
Rres		Rres	2017	Endo		Endo		2017		
Rres		Rres	2017	Endo		Endo		2017		
Rres		Rres	2017	Endo		Endo		2017		
Rres		Rres	2017	Endo		Endo		2017		
Rres		Rres	2017	Endo		Endo		2017		
Rres		Rres	2017	Endo		Endo		2017		
Rres		Rres	2017	Endo		Endo		2017		
Rres		Rres	2017	Endo		Endo		2017		
Rres		Rres	2016	Grain	Grain		2016		37.33784202	41.45905292
UoN	UoN		2016	Grain	Grain		2016		32.96919521	40.54319284
UoN	UoN		2016	Grain	Grain		2016		30.3652289	32.23564072
Rres		Rres	2016	Grain	Grain		2016		33.82310262	40.1472331
Rres		Rres	2016	Grain	Grain		2016		33.29999049	42.67260412
Rres		Rres	2016	Grain	Grain		2016		39.30910423	42.03152807
UoN	UoN		2016	Grain	Grain		2016		37.39661012	30.97041122
Rres		Rres	2016	Grain	Grain		2016		37.22027105	42.22066881
Rres		Rres	2016	Grain	Grain		2016		32.41633231	38.18360967
Rres		Rres	2016	Grain	Grain		2016		27.78398618	36.45807493
Rres		Rres	2016	Grain	Grain		2016		27.37496546	34.89491411
UoN	UoN		2016	Grain	Grain		2016		45.65535779	30.25274658
Rres		Rres	2016	Grain	Grain		2016		36.94717065	56.88488118
UoN	UoN		2016	Grain	Grain		2016		43.16135877	37.23686034
Rres		Rres	2016	Grain	Grain		2016		43.13737847	43.68343958
Rres		Rres	2016	Grain	Grain		2016		37.7158799	40.78876395
UoN	UoN		2016	Grain	Grain		2016		37.14871695	35.86342225
Rres		Rres	2016	Grain	Grain		2016		31.95059131	43.08533873
UoN	UoN		2016	Grain	Grain		2016		37.96222384	36.42643117

Rres		Rres	2016	Grain	Grain		2016	31.21448931	38.45793755
UoN	UoN		2016	Grain	Grain		2016	39.3162108	32.87269397
Rres		Rres	2016	Grain	Grain		2016	41.96131366	49.61469838
Rres		Rres	2016	Grain	Grain		2016	28.29095498	41.65436642
UoN	UoN		2016	Grain	Grain		2016	27.55308249	30.19698257
UoN	UoN		2016	Grain	Grain		2016	32.44823359	27.80849538
Rres		Rres	2016	Grain	Grain		2016	31.51282731	44.20358158
Rres		Rres	2016	Grain	Grain		2016	45.68349736	47.53324198
Rres		Rres	2016	Grain	Grain		2016	34.81881784	52.47731938
UoN	UoN		2016	Grain	Grain		2016	34.81014677	31.60145662
UoN	UoN		2016	Grain	Grain		2016	25.80571887	24.59533737
UoN	UoN		2016	Grain	Grain		2016	31.13006199	25.90015942
UoN	UoN		2016	Grain	Grain		2016	43.69653021	50.09922286
UoN	UoN		2016	Grain	Grain		2016	36.85089437	32.37636048
UoN	UoN		2016	Grain	Grain		2016	37.51083745	44.61063428
UoN	UoN		2016	Grain	Grain		2016	38.43151541	33.72200827
UoN	UoN		2016	Grain	Grain		2016	47.21187309	55.73576348
Rres		Rres	2016	Endo		Endo	2016		
UoN	UoN		2016	Endo		Endo	2016		
UoN	UoN		2016	Endo		Endo	2016		
Rres		Rres	2016	Endo		Endo	2016		
Rres		Rres	2016	Endo		Endo	2016		
Rres		Rres	2016	Endo		Endo	2016		
UoN	UoN		2016	Endo		Endo	2016		
Rres		Rres	2016	Endo		Endo	2016		
Rres		Rres	2016	Endo		Endo	2016		
Rres		Rres	2016	Endo		Endo	2016		
Rres		Rres	2016	Endo		Endo	2016		
UoN	UoN		2016	Endo		Endo	2016		
Rres		Rres	2016	Endo		Endo	2016		
UoN	UoN		2016	Endo		Endo	2016		
Rres		Rres	2016	Endo		Endo	2016		
Rres		Rres	2016	Endo		Endo	2016		

UoN	UoN		2016	Endo		Endo	2016		
Rres		Rres	2016	Endo		Endo	2016		
UoN	UoN		2016	Endo		Endo	2016		
Rres		Rres	2016	Endo		Endo	2016		
UoN	UoN		2016	Endo		Endo	2016		
Rres		Rres	2016	Endo		Endo	2016		
Rres		Rres	2016	Endo		Endo	2016		
UoN	UoN		2016	Endo		Endo	2016		
UoN	UoN		2016	Endo		Endo	2016		
Rres		Rres	2016	Endo		Endo	2016		
Rres		Rres	2016	Endo		Endo	2016		
Rres		Rres	2016	Endo		Endo	2016		
UoN	UoN		2016	Endo		Endo	2016		
UoN	UoN		2016	Endo		Endo	2016		
UoN	UoN		2016	Endo		Endo	2016		
UoN	UoN		2016	Endo		Endo	2016		
UoN	UoN		2016	Endo		Endo	2016		
UoN	UoN		2016	Endo		Endo	2016		
UoN	UoN		2016	Endo		Endo	2016		
UoN	UoN		2016	Endo		Endo	2016		
UoN	UoN		2017	Grain	Grain			2017	45.06476724	24.69580933
UoN	UoN		2017	Grain	Grain			2017	49.12105927	23.94392355
UoN	UoN		2017	Grain	Grain			2017	41.66491901	30.70082467
UoN	UoN		2017	Grain	Grain			2017	46.13040096	22.92305167
UoN	UoN		2017	Grain	Grain			2017	37.73373912	28.93154234
UoN	UoN		2017	Grain	Grain			2017	37.87816492	27.07841797
UoN	UoN		2017	Grain	Grain			2017	41.43099285	21.0767056
UoN	UoN		2017	Grain	Grain			2017	36.43632436	26.84963041
UoN	UoN		2017	Grain	Grain			2017	51.3825825	38.44229632
UoN	UoN		2017	Grain	Grain			2017	38.65194017	25.27481853
UoN	UoN		2017	Grain	Grain			2017	43.37630689	24.03823879
UoN	UoN		2017	Grain	Grain			2017	30.51920857	23.95821044
UoN	UoN		2017	Grain	Grain			2017	41.3707587	24.01184987

UoN	UoN		2017	Grain	Grain		2017	41.18153642	23.81965213
UoN	UoN		2017	Grain	Grain		2017	37.15928168	26.16284981
UoN	UoN		2017	Grain	Grain		2017	43.04406256	31.18571493
UoN	UoN		2017	Grain	Grain		2017	34.55200623	23.20712844
UoN	UoN		2017	Grain	Grain		2017	30.79953746	21.83880784
Rres		Rres	2017	Grain	Grain		2017	37.90393447	38.3297525
Rres		Rres	2017	Grain	Grain		2017	49.61549284	43.31096588
Rres		Rres	2017	Grain	Grain		2017	33.52209955	32.69343135
Rres		Rres	2017	Grain	Grain		2017	50.89774395	37.59672981
Rres		Rres	2017	Grain	Grain		2017	48.9485813	41.30094294
Rres		Rres	2017	Grain	Grain		2017	50.73052322	42.13857178
Rres		Rres	2017	Grain	Grain		2017	52.8805686	42.00637408
Rres		Rres	2017	Grain	Grain		2017	49.29453936	44.21207023
Rres		Rres	2017	Grain	Grain		2017	45.63028739	38.60783927
Rres		Rres	2017	Grain	Grain		2017	37.31962574	33.61888392
Rres		Rres	2017	Grain	Grain		2017	54.92237774	41.03233723
Rres		Rres	2017	Grain	Grain		2017	38.48596715	34.73107692
Rres		Rres	2017	Grain	Grain		2017	51.75782	37.88735832
Rres		Rres	2017	Grain	Grain		2017	43.81045202	37.36588511
Rres		Rres	2017	Grain	Grain		2017	49.14906678	35.19763988
Rres		Rres	2017	Grain	Grain		2017	45.51054422	43.46794432
Rres		Rres	2017	Grain	Grain		2017	46.10656765	51.59375001
Rres		Rres	2017	Grain	Grain		2017	50.74985616	49.7760569
UoN	UoN		2017	Endo		Endo	2017		
UoN	UoN		2017	Endo		Endo	2017		
UoN	UoN		2017	Endo		Endo	2017		
UoN	UoN		2017	Endo		Endo	2017		
UoN	UoN		2017	Endo		Endo	2017		
UoN	UoN		2017	Endo		Endo	2017		
UoN	UoN		2017	Endo		Endo	2017		
UoN	UoN		2017	Endo		Endo	2017		
UoN	UoN		2017	Endo		Endo	2017		
UoN	UoN		2017	Endo		Endo	2017		

UoN	UoN		2017	Endo	Endo	2017
UoN	UoN		2017	Endo	Endo	2017
UoN	UoN		2017	Endo	Endo	2017
UoN	UoN		2017	Endo	Endo	2017
UoN	UoN		2017	Endo	Endo	2017
UoN	UoN		2017	Endo	Endo	2017
UoN	UoN		2017	Endo	Endo	2017
UoN	UoN		2017	Endo	Endo	2017
Rres		Rres	2017	Endo	Endo	2017
Rres		Rres	2017	Endo	Endo	2017
Rres		Rres	2017	Endo	Endo	2017
Rres		Rres	2017	Endo	Endo	2017
Rres		Rres	2017	Endo	Endo	2017
Rres		Rres	2017	Endo	Endo	2017
Rres		Rres	2017	Endo	Endo	2017
Rres		Rres	2017	Endo	Endo	2017
Rres		Rres	2017	Endo	Endo	2017
Rres		Rres	2017	Endo	Endo	2017
Rres		Rres	2017	Endo	Endo	2017
Rres		Rres	2017	Endo	Endo	2017
Rres		Rres	2017	Endo	Endo	2017
Rres		Rres	2017	Endo	Endo	2017
Rres		Rres	2017	Endo	Endo	2017
Rres		Rres	2017	Endo	Endo	2017
Rres		Rres	2017	Endo	Endo	2017
Rres		Rres	2017	Endo	Endo	2017

Fe_mg_kg_Endo	Zn_mg_kg_Endo

21.16488245	11.25884388
12.10141449	9.832373121
15.72439615	9.806831067
22.83751776	9.038350456
22.78428752	9.729011578
11.1783285	7.367286905
21.55736094	10.7017942
26.07078648	10.3892241
20.0879467	12.02881462
27.39916053	13.41223825
16.2946181	8.641459632
24.46522911	9.282429699
27.22284994	11.89107471
9.731362144	8.558681258
13.20186766	9.797557507
30.80148839	6.761374849
19.37841484	9.605263527
11.68698963	9.449316376
23.62460343	7.07869843
10.62733525	10.58506112
28.02964484	8.948329233
16.12206819	10.49305769
7.747854828	8.001439231
14.37163935	9.475495024
13.31491125	9.923109559
6.762017861	6.891887961

12.73747349	10.75066825
19.04206741	9.209121889
15.87587949	8.878726819
14.49365728	11.45649925
9.052209183	8.285766351
13.12365059	7.545536738
7.189140018	7.235534167
10.69652526	12.62726452

12.11604347	5.719726009
11.61814284	7.587039982
11.30954816	4.995041156
9.915528776	5.035135557
9.12606285	4.676886859
9.058319292	5.634945585
10.12190567	6.09920413
11.00147297	7.960180387
11.51102184	5.970226435
11.39308196	5.120325591
10.3338478	6.389730041
8.136174391	7.156997065
7.95938211	6.686372469
15.03456681	7.28451979
13.79523851	9.358251881
5.353371848	4.411371479
8.724101633	4.90160387
9.231081907	5.009059144
11.07419802	8.678441488
11.64902657	17.22840813
11.8199795	13.63529262
10.79556327	13.72367117

11.50655066	11.42835904
11.84815485	11.65778901
9.387359016	11.24126073
9.974494682	9.986360339
10.71681572	15.20297294
9.465391871	11.37472291
9.179063998	12.39526304
8.710867097	7.539515479
11.72081613	13.79970874
10.24137439	9.912597416
7.871687202	7.427920209
10.06408421	11.50887137
9.903209929	11.96157861
11.73192879	13.94765936

15.27834907	8.030236356
12.40971589	8.309257143
19.91882462	10.87242402
14.92546501	13.25951355
25.64641848	10.21706911
24.20243744	11.8250155
8.956070746	7.707927319
25.77223143	12.75184913
20.95541214	9.532846914
21.69537154	9.18281208
9.167151054	9.510794993
6.720697309	7.578493573
15.22327083	10.06142033
6.984165617	8.587024239
7.398745127	12.00669721
7.975201009	8.79503009
6.635747206	11.24200444
11.6592459	8.359552028
18.39539981	14.458367
18.26393144	12.6050477
9.027503359	7.695607266

10.3719767	5.600450532
9.746164432	5.776265404
6.705081477	4.511327681
9.98134751	11.51780792

9.29088152	10.66686925
8.536125111	11.38883537

22.00302225	14.20330781
22.18197201	10.15651537
13.25905171	11.09626242
30.86259865	13.21984399
17.83602922	16.54381695
18.19421732	11.51164848
25.48774294	18.41459704
14.55600059	11.97740289
18.85014306	19.27290444
10.11847983	12.64853841
16.83356535	9.498006439
12.02751654	13.58861276
19.71005431	14.38439706
12.96627477	8.017359856
14.88713668	13.0607271
15.3517117	12.41925437
19.47626023	13.57133172
13.38031993	14.26216874
15.35289301	15.8496733
12.72034777	13.13765058
17.85949464	16.94861426
16.15411365	11.09324711
13.37138665	11.22271135
27.28684814	13.96600285
13.33462164	16.34080119
10.13124709	13.33226765

14.54446612	6.41839088
14.62164445	14.59292668
12.01899974	14.83305624
9.090691245	13.91985594
14.70497135	15.37216849
12.74498198	9.229959096

8.663842038	8.369659472
8.124405103	6.915173518
15.14158693	9.558078065
15.08006396	10.41923828
13.73708232	8.820668074
10.65629362	7.152873994
11.01815714	8.764506985
14.66955001	8.85864469
9.679312363	6.357262901
8.820656514	6.947174128
13.80233842	10.08717188
	12.09652852
16.63718579	10.39755557
18.02978949	10.6540638
9.585454601	7.45213782
12.19179135	9.606176755
17.26124616	11.9393723
9.701511674	7.717953816
10.4278507	11.3744598
8.313915799	11.14767001
11.15765755	14.51024545
11.03691255	16.20461902

9.086813629	14.30373571
12.41446938	16.28800366
13.10157131	14.38543954
16.44777017	19.55403279
8.865803904	12.50190415
11.03312187	14.45433445
14.82568068	18.62425947
9.571132185	10.53631781
9.268775702	12.28285759
11.51837808	17.91246645
10.98468635	15.31815674
12.63122198	17.36820158
11.57411835	15.35189807
7.214248315	9.387403098

17.33609981	11.60619047
12.99402934	11.84221487
15.64168844	11.79944394
18.34774902	12.68838441
	13.7035112
17.6264843	13.72875201
34.12697206	15.09887333
21.36476791	14.37952292
13.83959362	13.10526915
17.41273896	15.11646265
15.26108488	10.61655591
28.10580725	11.76395422
17.76970592	14.9236168
31.21902197	13.50645664
13.55760972	11.73843539

9.04731907	9.266902498
12.50145592	11.95552337
14.5475188	14.99641299
15.35241804	13.55189965
14.76769213	14.2066521
14.09207274	17.14474119
11.15557124	13.93286174
10.90619139	10.14222203
14.86804098	11.81243037
18.93001178	13.93260921
10.35920513	11.8110017
14.90723367	16.82945722
11.91255913	10.58067139
10.77050819	8.991073117
18.70145553	12.07388017
16.58986192	13.04856494
14.85957285	13.07528349
7.725453763	12.19726228
19.39430713	11.99773902
14.97264973	11.46834672

12.37947758	8.907129968
13.17999337	7.446588919
10.0072258	8.481945033
12.79726987	8.899039159
10.60599602	9.048551571
13.61155885	8.384263283
13.74142207	8.062566962
9.659835236	7.062775088
13.39781328	14.71723536
12.4365132	8.08757844

10.00383792	6.790086416
12.18403226	7.765981603
15.19548	7.755896423
12.85995526	7.297994013
8.390891661	6.655896416
7.943630649	8.105895594
11.38896539	10.36070615
7.38455258	6.409665828
8.772928721	14.55586651
9.558762865	14.70650207
8.419588766	9.026334059
11.16066359	13.5487767
9.363299929	12.48286202
13.5392506	13.34059427
10.25178027	11.70109376
11.77199002	14.4677604
10.95282164	14.2328239
10.06197166	10.90845769
14.20514515	12.53942675
8.71161753	10.44185019
12.04501733	12.93044964
8.774165126	11.56785763
11.55490723	12.6227369
10.26837555	14.02589726
9.947425115	14.80183089
9.916192636	15.16381983
